# Supplementary material for: Catalytic disconnection of C–O bonds in epoxy resins and composites
Source: Nature. 2023 Apr 26;617(7962):730–7. doi: 10.1038/s41586-023-05944-6 (PMC10208972; doi:10.1038/s41586-023-05944-6)
Supplement: Supplementary file 1 — Supplementary Information [file 41586_2023_5944_MOESM1_ESM.pdf]

nature portfolio

https://doi.org/10.1038/s41586-023-05944-6

Supplementary information

Catalytic disconnection of C–O bonds in epoxy resins and composites

In the format provided by the authors and unedited

Supplementary Information

Catalytic Disconnection of C–O Bonds in Epoxy Resins and Composites

Alexander Ahrens[1]*, Andreas Bonde[1], Hongwei Sun[1], Nina Kølln Wittig[1], Hans Christian D. Hammershøj[1], Gabriel Martins Ferreira Batista[1], Andreas Sommerfeldt[2], Simon Frølich[2], Henrik Birkedal[1], Troels Skrydstrup[1]*

[1]Department of Chemistry and Interdisciplinary Nanoscience Center (iNANO), Aarhus University, Gustav Wieds Vej 14, 8000 Aarhus C, Denmark.

[2]Danish Technological Institute, Kongsvang Allé 29, 8000 Aarhus C, Denmark.

*Corresponding author. Email: aahrens@inano.au.dk, ts@chem.au.dk

Contents

1. General Information ... 3
   1.1. Polymer/Composites samples ... 5
2. Optimization Tables ... 11
   2.1 Methodologies Reported for Lignin Valorization ... 11
   2.2 Screening of Ligands ... 13
   2.3 Screening of Hydrogen Source ... 14
   2.4 Screening of *i*PrOH Amount ... 14
   2.5 Screening of Solvents ... 15
   2.6 Screening of Structurally Related Catalysts and Acid Co-Catalysts ... 15
   2.7 Variation of Conditions on Airstone 76E/766H and Analysis of Powders ... 16
3. Mechanistic Investigation ... 19
   3.1 Kinetic Study in Model 1 ... 19
   3.2 Detection of Acetone as Corresponding Disconnection Product ... 23
   3.3 Activation of Precatalyst - Operando Monitoring *via* NMR Spectroscopy ... 24
   3.6 Identification of triphos-Ru-H_2-CO in Reaction Mixture ... 25
   3.4 Hydride-Bridged Binuclear Ruthenium(I) Complex ... 27
4. Synthesis of Compounds ... 28
   4.1 Complexes ... 28
   4.2 Model Substrates ... 30
5. Catalytic Deconstruction ... 38
   5.1 On Model Substrates ... 38
   5.2 On Amine-Cured Epoxy Resins ... 40
   5.2 On Fiber Reinforced Epoxy Composites ... 41
   5.3 Control Experiment on Wind Turbine blade Composite ... 43
6. Analytical Data ... 44
   6.1 NMR Spectra of Synthesised Compounds ... 44
   6.2 Spectra of Rest Fraction ... 61
   6.3 Characterisation of Recovered Fibers ... 63

6.3.1 Microscopic Images 63
6.3.2 IR spectra 66
6.3.3 X-ray photoelectron spectroscopy 67
6.3.4 SEM images 68
6.3.5 Tensile Strength Testing 69
6.4 X-Ray Crystallographic Data 71
6.5 Density Functional Theory (DFT) Study 73
7. References 75

1. General Information

Unless otherwise stated, all reactions were set-up and worked up in a glovebox under an atmosphere of argon. All chemicals were purchased from Sigma-Aldrich, Tokyo Chemical Industry (TCI) or Strem Chemicals and used as received. THF, toluene, CH_2Cl_2 and MeCN were retrieved from a MBraun SP-800 purification system, degassed using argon and stored over 3 Å molecular sieves. The remaining solvents were purchased from Sigma-Aldrich degassed using argon, stored over 3 Å molecular sieves and used without further purification.

Thin layer chromatography (TLC) was carried out on pre-coated aluminium sheets ALUGRAM® Xtra SIL G/UV254 purchased by Macherey-Nagel. Visualisation of the products was achieved by UV-light irradiation (366 nm) and / or staining with a potassium permanganate in water.

Flash column chromatography was carried out using Silica gel (0.040 – 0.063 mm/ 230 – 400 mesh) ASTM purchased from Macherey-Nagel. Automated flash column chromatography (AFCC) was carried out with Interchim PuriFlash XS520Plus with 30 µm prepacked columns. Celite®545, coarse, was used for filtration.

Gas chromatography - mass spectrometry (GC-MS) were measured with an Agilent 8890 gas chromatograph coupled with an Agilent 5977B mass selective detector.

High resolution mass spectrometry (HRMS): ESI(+) spectral analysis were measured with a Bruker Maxis Impact Spectrometer. MALDI spectral analysis were measured on a Bruker Autoflex maX MALDI-TOF MS spectrometer using a MTP 384 target plate polished steel BC.

Infrared Spectroscopy: IR spectra were measured with a Bruker ALPHA II FT-IR spectrometer and processed with OPUS (release 8.7).

Nuclear Magnetic Resonance spectroscopy: ^{1}H NMR, ^{13}C NMR and ^{31}P NMR spectra were recorded on a Bruker 400 MHz Ascend spectrometers. Chemical shifts were given as δ value (ppm) with reference to residual solvent signal of the deuterated solvent. The peak patterns are indicated as follows: s, singlet; d, doublet; t, triplet; m, multiplet; q, quartet. Multiplicities reported for ^{13}C NMR spectra were assigned using DEPT-90 and/or DEPT-135 spectra. The coupling constants, *J*, are reported in Hertz (Hz). The spectra were calibrated to the residual solvent signals[1]. NMR spectra were processed with MestReNova Version 14.2.1-27684.

Particle size distributions were determined using a Malvern Mastersizer 2000 instrument with a Hydro S dispersion unit. The measurements were performed by means of laser diffraction and particles in the size interval from 0.02-2000 µm were measured. The sample was measured with constant stirring to avoid sedimentation according to ISO13320:2020 using a stirring rate of 3500 rpm and laser wavelengths of 633 nm and 466 nm. A refractive index of 1.5 and an absorption of 0.1 was assumed for the size distribution modelling of a sample of spherical particles. The result is reported as an average of triplicate measurement.

X-ray micro-computed tomography (µ-CT) was measured with an Xradia 620 Versa (ZEISS, Germany) to assess fiber organization and diameter; X-ray µ-CT being very helpful in analyzing hierarchical or multiscale materials[2]. The X-ray energy and power were adjusted between 40-50 kV and 3-4.5 W, respectively, with little to no attenuation to obtain optimal imaging conditions for these types of materials. The exposure times were then adjusted to reach ~ 5000 counts per pixel in the recorded projections. For each sample, an overview tomogram with 3.8 µm isotropic voxel size was collected with a 0.4× objective and 1601 projections/360°. This was used to select a region for high-resolution scanning in local tomography with a 4× objective, using 3201 projections/360° for glass fiber samples and 4801 projections/360° for the carbon fiber sample to result in isotropic voxel sizes of 1.0 µm and 0.3 µm, respectively. The data were analysed using Dragonfly Version 2021.3 (Object Research Systems Inc, Montreal, Canada) and MATLAB Version R2017b (MathWorks Inc., Massachusetts, USA).

X-ray photoelectron spectroscopy (Kratos Axis UltraDLD) was used with a monochromated Al X-ray source (1486.7 eV) operated at 225 W and residual pressure in the 10^{-9} torr range. High resolution scans were recorded at 20 eV pass energy and 0.1 eV step size while survey scans were collected with 160 eV analyzer pass energy and 1.0 step size. CasaXPS software–version 2.3.16 (Casa Software Ltd, Wilmslow, Cheshire, UK) was used to process the data and spectral calibration was achieved by using the C 1*s* peak (284.8 eV) of adventitious carbon.

Scanning Electron Microscopy (SEM) images were acquired with a TESCAN CLARA microscope (TESCAN Brno, CzechRepublic) operated in Depth mode. Secondary electron (SE) contrast images were taken at 5 keV, 131-442 pA, and a working distance of 5 mm using an Everhart-Thornley (E-T) detector. Backscattered electron (BSE) contrast images were taken at 15 keV, 1.19 nA, and a working distance of 9.8 mm using a low-energy four-quadrant BSE detector. Glass fibers were put on SEM stubs with carbon tape and coated with 8 nm Pt (Leica EM SCD500, Ballerup, DK) prior to SEM imaging.

Reaction set up: Unless stated otherwise, all catalytic deconstruction reactions were set up in an Argon charged glovebox using a 10 ml or 40 ml COtubes sealed with PTFE/silicon seals purchased from SyTracks as reaction vessel, a Teflon-coated stirring bar with dried and degassed solvents. Reactions were stirred in metal heating blocks at 650 rpm. **Warning: Glassware under pressure.**

- glass equipment should always be examined for damages to its surface, which may weaken its strength
- one must abide to all laboratory safety procedures and always work behind a shield when working with glass equipment under pressure
- COware is pressure tested to 224 psi but should under no circumstances be operated above 60 psi (5 bar)

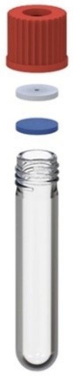

Fig. S1. 10 ml COtube with screw cap, Teflon disc and septum.

1.1. Polymer/Composites samples

The sources for the commercial samples reported in the manuscript are listed hereafter. Furthermore, for the cured epoxy resins the BPA contents were calculated based on the components listed in the safety data sheets available online and taking ^{1}H NMR spectra of the epoxy component. The determined BPA contents are approximations made to the best of our knowledge and analytical tools available, but do not present absolute values.

1) The infusion resin for wind systems **Airstone 760E/766H** (Fig. 3 a)) was provided by Olin Corporation and prepared by mixing 760E (100 g) with 766H (32.0 g), which was then degassed for 20 min under vacuum, poured into preheated silicone moulds (50 °C) and cured at 50 °C for 1 h and 80 °C for 3 h to give the hardened resin.

According to the corresponding safety data sheets, 760E contains

- > 75.0% bis-[4-(2,3-epoxipropoxi)phenyl]propane (DGEBA)
- < 25.0% 1,4-bis(2,3-epoxypropoxy)butane (BDDE)

while 766H contains

- ≤ 50.0 < 75.0% poly(oxypropylene) diamine
- ≤ 25.0 < 50.0% 3-aminomethyl-3,5,5-trimethylcyclohexylamine

^{1}H NMR spectra of 760E in $CDCl_3$:

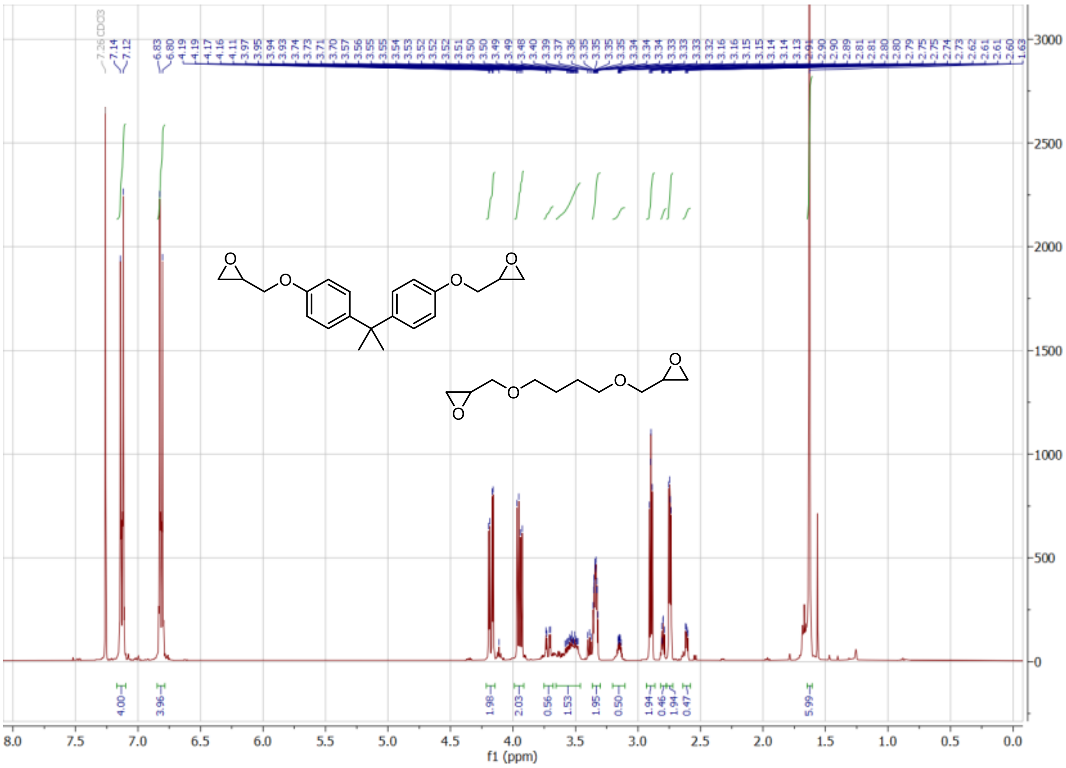

- according to the spectra 760E consisting of approximately 80% DGEBA and 20% BDDE, which corresponds to 87 wt% DGEBA and 13 wt% BDDE
- DGEBA itself contains 67 wt% BPA
- cured Airstone 760E/766H contains 75 wt% of 760E
  - ➢ approx. BPA content = 75 wt% * 87 wt% * 67 wt% = **43 wt%**

2) The two-component adhesive (**UHU plus endfest** 2-K-Epoxidharzkleber 45670) (Fig. 3 b)) produced by UHU GmbH & Co. KG was prepared by mixing both components 1 g to 1 g and then letting it harden over-night at room temperature.

According to the corresponding safety data sheets the epoxy contains

- bisphenol-A-(epichlorhydrin); epoxy resin (average molecular weight ≤ 700)

while the hardener contains

- 50 - 100% fatty acids, C_{18}-unsatd., dimers, polymers with oleic acid and triethylenetetramine, polyamide resin
- 25 - 50% amines, polyethylenepoly-, tetraethylenepentamine fraction
- 2.5 - 10% tetraethylenepentamine, linear, cyclic and branched
- < 1% triethylentetramine

^{1}H NMR spectra of UHU epoxy component in $CDCl_3$:

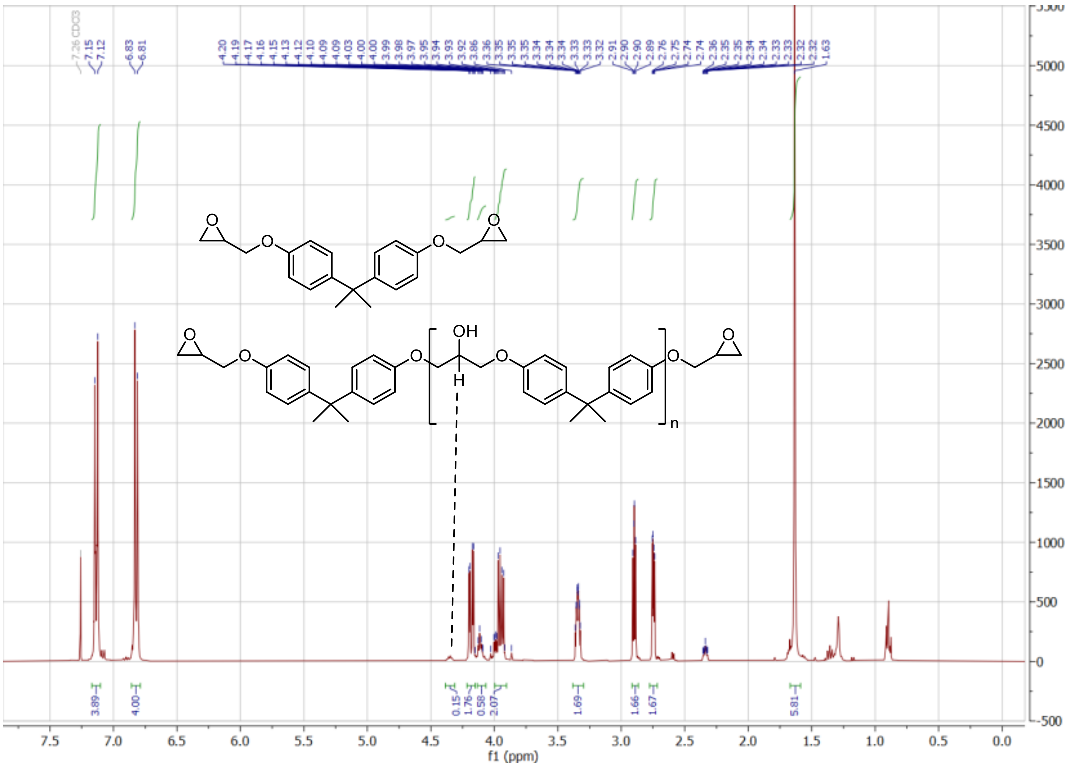

- according to the spectra the epoxy component consists of approximately 85% DGEBA and 15% higher oligomers
- DGEBA itself contains 67 wt% BPA, while the oligomers contain approx. ~74 wt% BPA
- the cured adhesive contains 50 wt% of epoxy component
    - ➢ approx. BPA content = 50 wt% * (67 wt% * 85% + 74 wt% * 15%) = **34 wt%**

3) The clear cast resin for handicraft (**Roizefar Epoxy Resin**) (Fig. 3 c)) produced by Shenzhen Fengao Technology Co., Ltd. was prepared by mixing both components in a 1 g to 1 g ratio and hardened overnight at room temperature. For this resin, no safety data sheets could be found online.

A GCMS of Roizefar epoxy component showed the masses of DGEBA (m/z 340.1) and 2-((tolyloxy)methyl)oxirane (m/z 164.1, cresol based epoxy).

^{1}H NMR spectra of Roizefar epoxy component in $CDCl_3$:

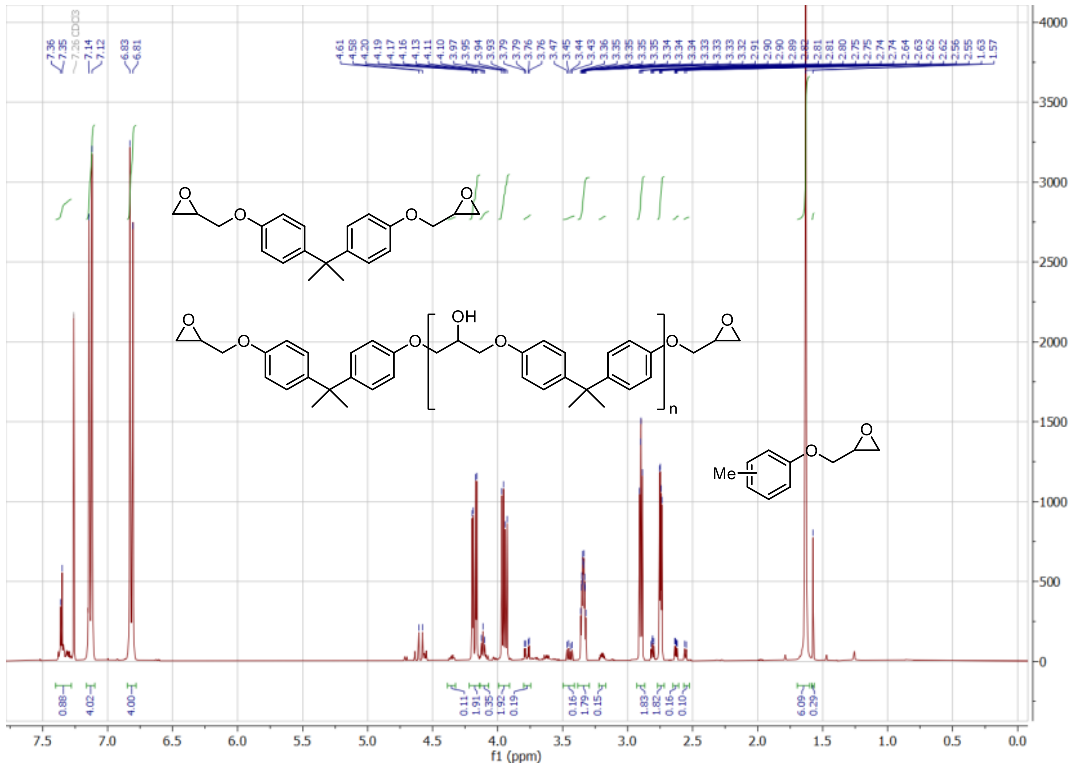

- according to the spectra the epoxy component consists of approximately 80% DGEBA and 10% higher oligomers
- DGEBA itself contains 67 wt% BPA, while the oligomers contain approx. ~74 wt% BPA
- the cured adhesive contains 50 wt% of epoxy component
    - ➢ approx. BPA content = 50 wt% * (67 wt% * 80% + 74 wt% * 10%) = **30 wt%**

4) The infusion resin for maritime systems **Sicomin SR infugreen 810/SD8822** (Fig. 3 d)) produced by Sicomin Epoxy Systems was prepared by mixing SR infugreen 810 (100 g) with SD8822 (32.0 g), which was then degassed for 20 min under vacuum, poured into preheated silicone moulds (50 °C) and cured at 50 °C for 1 h and 80 °C for 3 h to give the hardened resin.

According to the corresponding safety data sheets the epoxy contains

- ○ 50 ≤ x% < 100 bisphenol-A-(epichlorhydrin); epoxy resin (number average molecular weight ≤ 700)
- ○ 10 ≤ x% < 25 1,4-bis(2,3-epoxypropoxy)butane (BDDE)
- ○ 2.5 ≤ x% < 10 bisphenol-F-(epichlorhydrin); epoxy resin (number average molecular weight ≤ 700)

while the hardener contains

- ○ 50 ≤ x% < 100 3-aminomethyl-3,5,5-trimethylcyclohexylamine
- ○ 25 ≤ x% < 50 poly(oxypropylene) diamine
- ○ 2.5 ≤ x% < 10 trimethylolpropane tris[poly(propylene glycol), amine terminated] ether

^{1}H NMR spectra of SR infugreen 810 in $CDCl_3$:

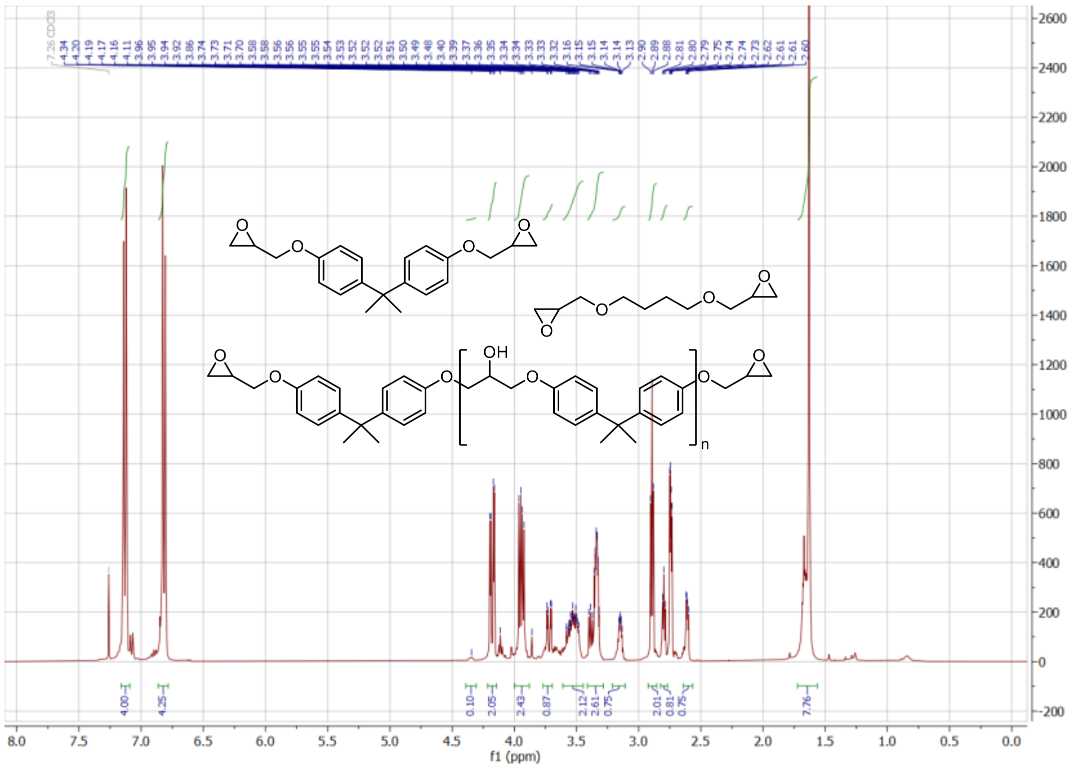

- the spectra reveals that the epoxy component consists of approximately 61% DGEBA, 10% higher oligomers and 29% BDDE
- DGEBA itself contains 67 wt% BPA, while the oligomers contain approx. ~74 wt% BPA
- the cured adhesive contains 75 wt% of epoxy component
    - approx. BPA content = 75 wt% * (67 wt% * 61% + 74 wt% * 10%) = **36 wt%**

5) The anhydride cured pultrusion resin Lightstone 3100E/3102H was provided as a cured clear cast sample by Olin Corporation. As a cured sample was provided, no NMR spectroscopy or GC-MS could be measured pre-curing. The BPA content is approximately **33 wt%** according to Olin Corporation.

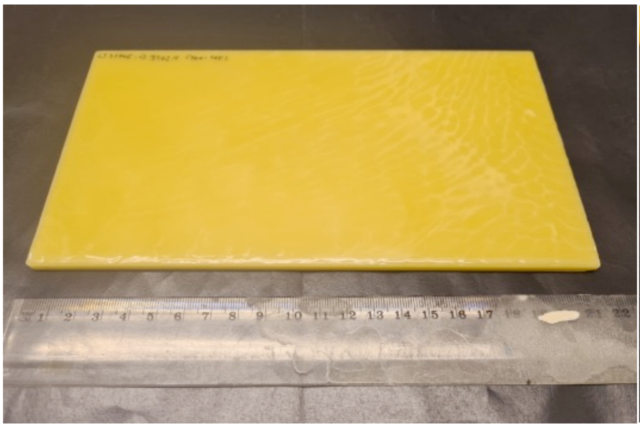

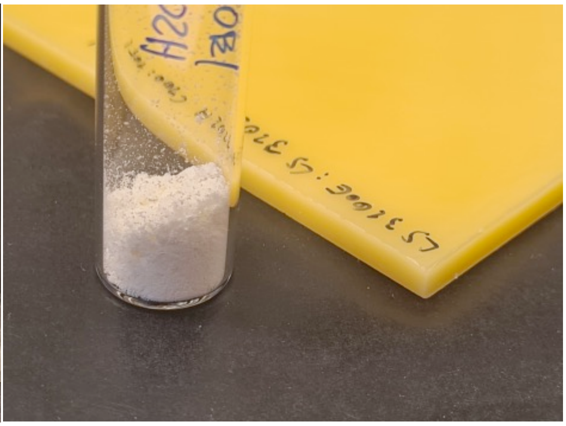

According to the corresponding safety data sheets the epoxy (Lightstone 3100E) contains
- 100% 1,4-bis(2,3-epoxypropoxy)butane (BDDE)

while the hardener (Lightstone 3102H) contains
- 50 ≤ x% < 60 Tetrahydro-4-methylphthalicanhydride
- 20 ≤ x% < 30 1,2,3,6-tetrahydro-3-methylphthalic anhydride
- 10 ≤ x% < 20 polypropylene glycol
- 1 ≤ x% < 5 1,2,3,6-Tetrahydrophthalic anhydride
- x% < 5 Benzyltriethylammonium chloride

6) A **carbon fiber-based epoxy composite** (Fig. 4A, a)) in form of a black plate was recovered **from a landfill** in Aarhus, Denmark. Unfortunately, it was not possible to determine what that material had been a part of originally. The composite was cut to size with a hacksaw and used without any other prior treatment.

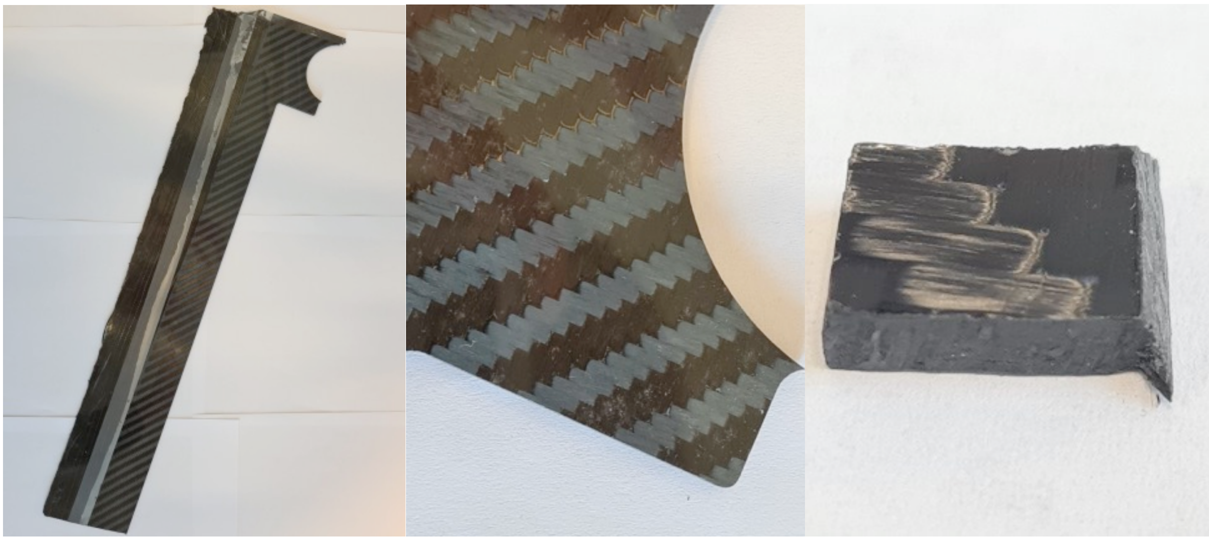

Fig. S2. Carbon fiber-based epoxy composite recovered from a landfill and a piece of it cut to size that was used in the deconstruction. The disassembled piece on the right is between 1 cm to 1.5 cm in length and width and weighted 187 mg.

7) A **product sample** of a **glass fiber-based composite** (Fig. 4A, b)) was provided by Olin Corporation presenting a commercialised material and was cut to size with a hacksaw and used without any other prior treatment.

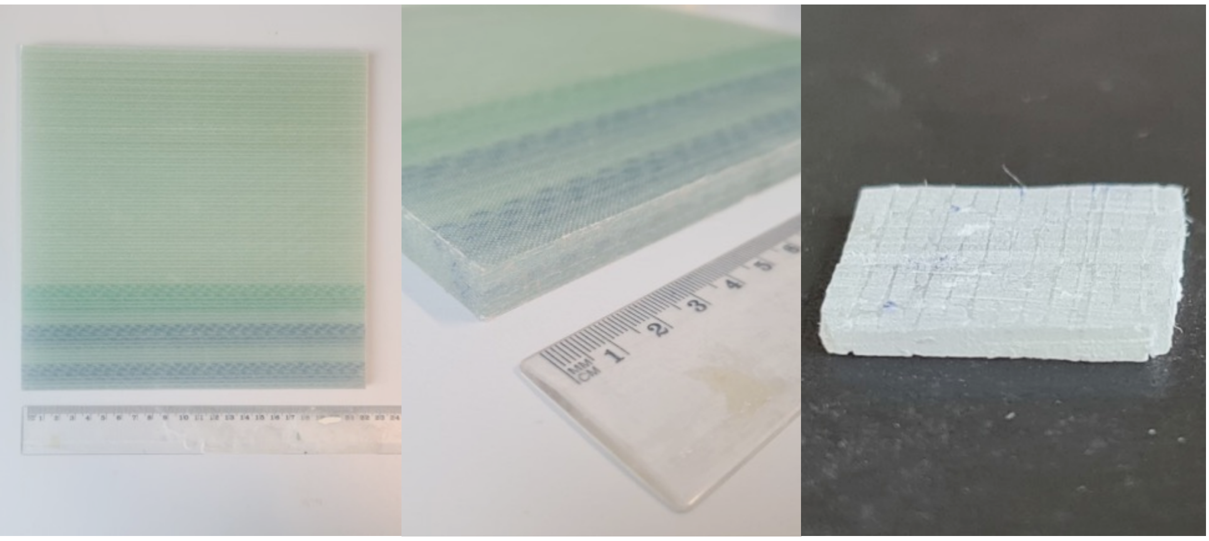

Fig. S3. A product sample of a fiber glass and a piece of it cut to size that was used in the deconstruction. The disassembled piece on the right is between 1 cm to 1.5 cm in length and width and weighted 390 mg.

8) A piece of a **decommissioned wind turbine blade** (Fig. 4A, c) and Fig. 4B) was provided by Vestas Wind Systems A/S. The glass fiber-based material had been part of the outer shell of the blade and was cut to size with a hacksaw and used without any other prior treatment.

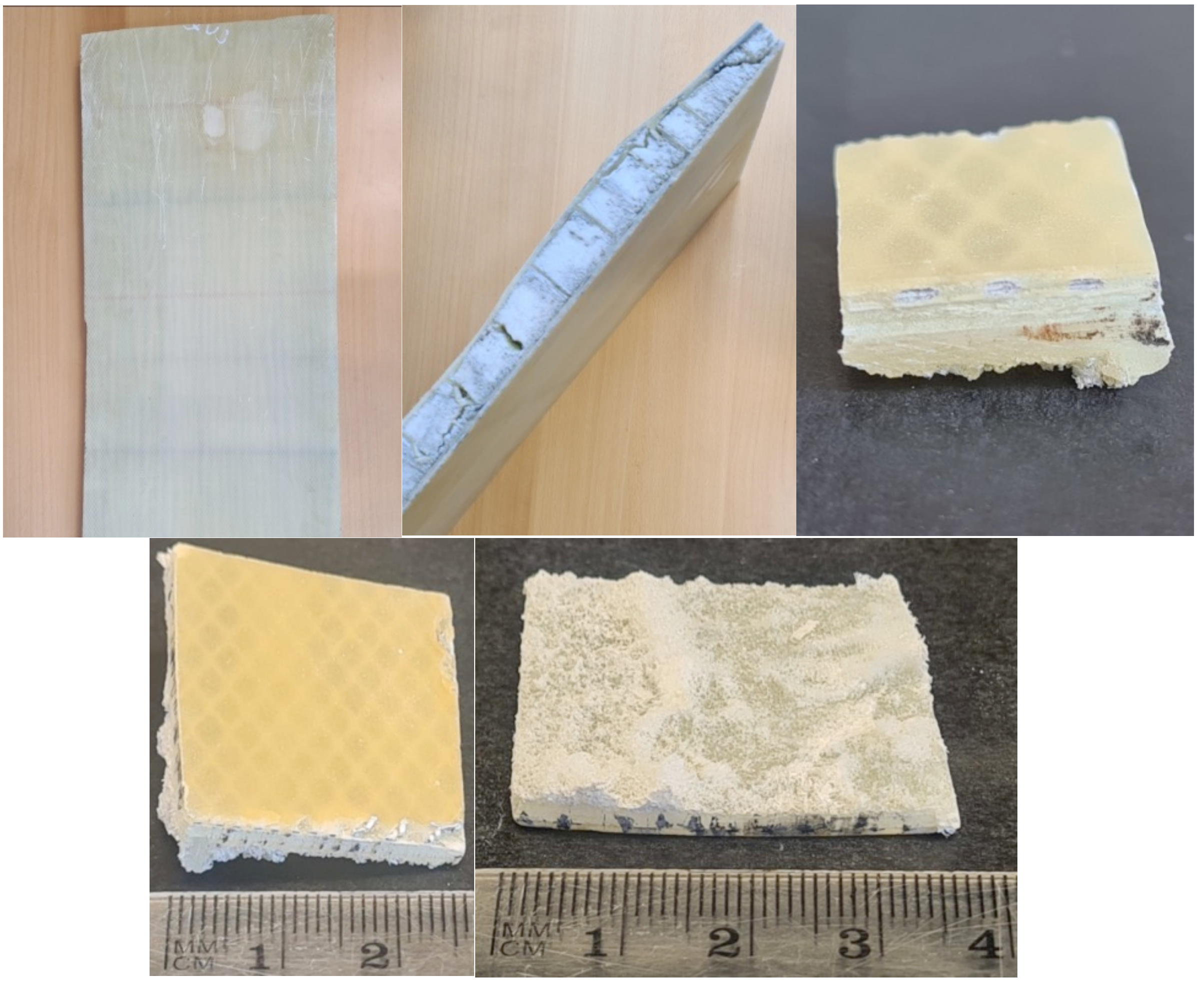

Fig. S4. A piece of a decommissioned wind turbine blade that was cut to size for the deconstruction. The disassembled piece on the right (Fig. 4A, c)) is between 1 cm to 1.5 cm in length and width and weighted 216 mg.

2. Optimisation Tables

For optimisation reactions 43.3 mg (0.08 mmol, 1 equiv) of the substrate were dissolved in 0.2 ml of solvent, then catalyst/metal salt/ligand were added. After sealing the reaction vessel, the mixtures were stirred outside of the glovebox in aluminium heating blocks. After the given reaction time, 1,3,5-trimethoxybenzene was added to the reaction mixture under air. Yields were determined by ^{1}H NMR spectroscopy of the crude mixture with 1,3,5-trimethoxybenzene as internal standard. GC-MS was used to confirm the products detected *via* ^{1}H NMR spectroscopy for all entries.

2.1 Methodologies Reported for Lignin Valorisation

For initial investigations of C–O bond cleavage on epoxy models, methodologies reported for the β-O-4 motif in lignin[3, 4] were considered (Fig. S5). The mechanism of the cleavage is assumed to go through acceptorless dehydrogenation of the alcohol moiety. Afterwards, the C–O bond adjunct to the ketone can be activated by the Ruthenium catalyst. The liberated hydrogen is then consumed, liberating acetophenone, phenol and the active catalyst[3].

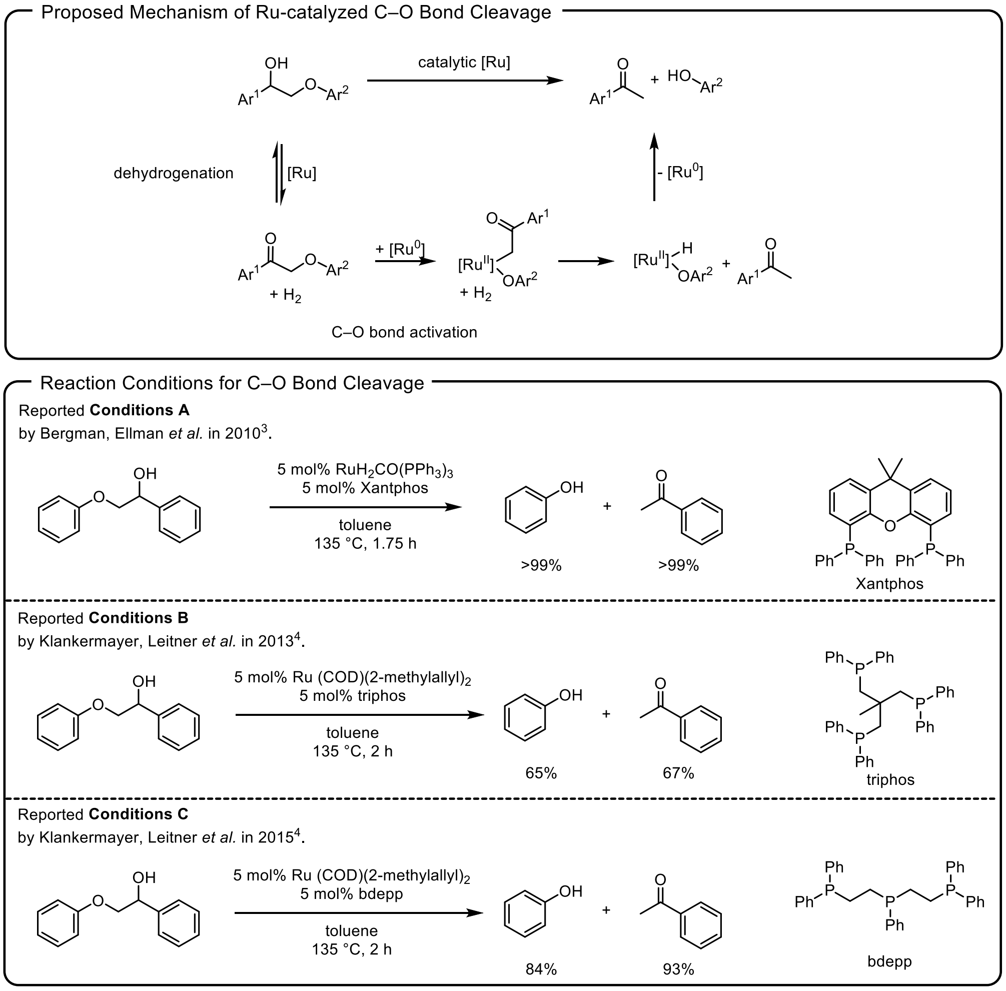

Fig. S5. Ruthenium-catalysed C–O bond cleavage reported for lignin models.

Table S1. Initial attempts to transfer the reported procedures to epoxy **model 1** including some variations:

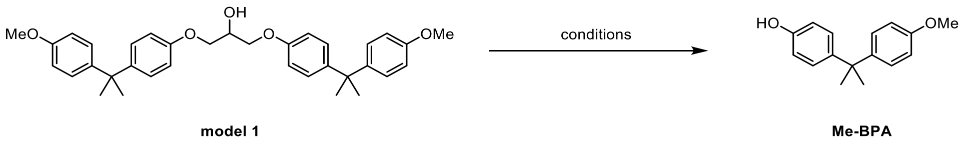

Entry	Conditions / Variations	Consumption	Me-BPA
	**Conditions A**		
**1**	no variations	>0%	not detected
**2**	160 °C, 16 h	13%	traces
**3**	200 °C, 16 h, mesitylene as solvent	8%	15%
	**Conditions B**		
**4**	no variations	>0%	not detected
**5**	160 °C, 16 h	8%	traces
**6**	160 °C, 16 h, triphos-Ru-TMM[a)]	23%	4%
	**Conditions C**		
**7**	no variations	>0%	traces
**8**	160 °C, 16 h	13%	11%
**9**	160 °C, 16 h, bdepp-Ru-TMM[a)]	47%	27%

a) 5 mol% of isolated complex were used as catalyst instead of *in situ* forming the corresponding complex.

It can be concluded that despite superficial similarities, epoxy model 1 and β-O-4 lignin models are not interchangeable in their chemical behavior. It must be considered, that the β-O-4 motif is based on a benzylic alcohol, while the epoxy motif is based on a secondary alkyl alcohol. The dehydrogenation of benzylic alcohols leads to conjugated ketones, which is not true for the epoxy motif. Another major difference is that the epoxy **model 1** contains two aryl ether C–O bonds adjacent the alcohol, calling for the presence of an additional hydrogen source.

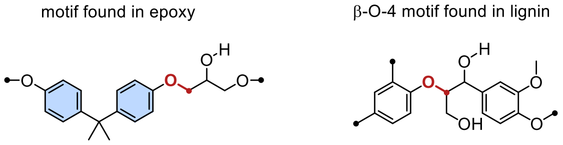

It is also noteworthy that on epoxy models, isopropanol deactivates bdepp-Ru-TMM while it activates triphos-Ru-RMM (see table S1 entry 6 and 8 vs. table S2 entry 3 and 4).

2.2 Screening of Ligands

Using epoxy **model 1**, a screening of different phosphine ligands using (COD)Ru(2-methylallyl)$_2$ as Ruthenium source in toluene as solvent was conducted. 3 mol% of catalyst loading, a reaction temperature of 160 °C and a reaction time of 16 h were chosen. In order to account for the two aryl ether C–O bonds that need to be cleaved, 1 equiv isopropanol was added as additional hydrogen source.

Table S2. Ligand screening.

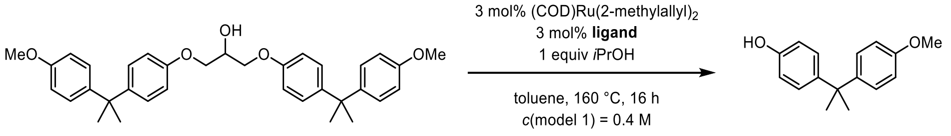

model 1 **Me-BPA**

Entry	Ligand (Variation)	Consumption	Me-BPA
**1**	triphos[a)]	24%	2%
**2**	triphos[b)]	87%	63%
**3**	triphos-Ru-TMM[c)]	94%	85%
**4**	bdepp[b)]	14%	traces
**5**	tBuXPhos (6 mol%)[b)]	10%	not detected
**6**	dppe[b)]	22%	traces
**7**	Xantphos[b)]	19%	traces
**8**	rac-BINAP[b)]	26%	3%
**9**	BiPhePhos[b)]	24%	not detected
**10**	PNP[b)]	23%	not detected
**11**	MACHO (6 mol% KOtBu)[b)]	11%	not detected

a) Ligand, [Ru] source and model 1 were dissolved in *i*PrOH/toluene and then heated to 160 °C for 16 h. b) Ligand and [Ru] source in toluene were heated to 130 °C for two hours then cooled to room temperature. Model 1 and *i*PrOH were added under argon, the mixture was then heated to 160 °C for 16 h. c) Instead of ligand and [Ru] source, triphos-Ru-TMM was used as catalyst.

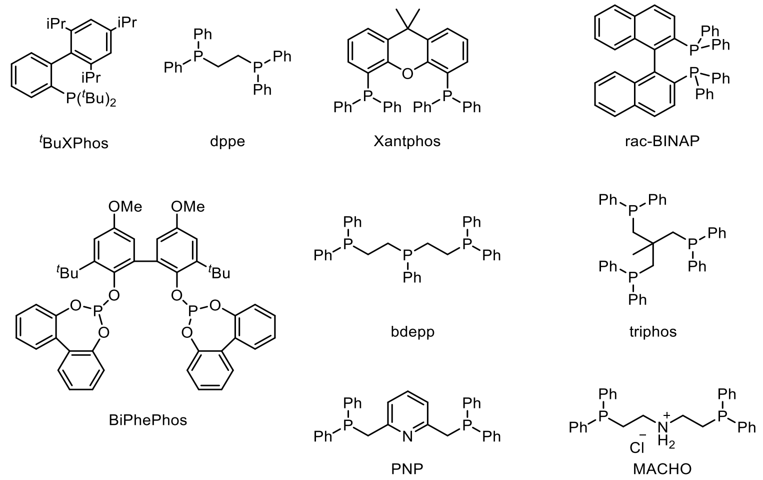

As the comparison between entry 1 and 2 shows, mixing model 1 with the ligand, ruthenium source and substrate did not lead to the formation of an active catalyst. However, pre-forming the ruthenium complex by heating ligand and ruthenium source for 2 hours at 130 °C before adding substrate and isopropanol led to moderate conversion with triphos as ligand. All other ligands were therefore tested using this protocol. Interestingly, the presence of isopropanol activated the triphos based complex, while deactivating the bdepp based ligand (as compared to table shown on 2.1).

2.3 Screening of Hydrogen Source

Using triphos-Ru-TMM as catalyst, other hydrogen sources than isopropanol were tested.

Table S3. Hydrogen source screening.

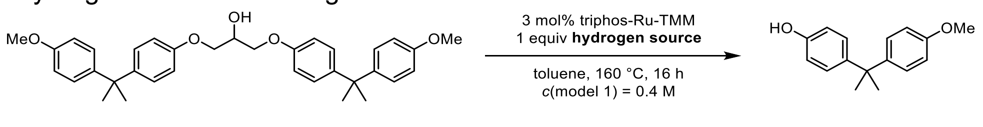

Entry	Hydrogen Source	Consumption	Me-BPA
**1 (ref)**	*i*PrOH	94%	85%
**2**	MeOH	34%	12%
**3**	EtOH	39%	16%
**4**	2-Phenylethanol	85%	86%
**5**	3 equiv H_2 (*ex situ* from Zn/HCl)[a)]	20%	0%

a) The hydrogen equivalents were released *ex situ* in a two-chamber set up with reflux condenser.

Although 2-phenylethanol as hydrogen source led to a slight increase in yield, isopropanol was considered a better reagent, due to its availability and easier separation from reaction products.

2.4 Screening of *i*PrOH Amount

Table ST4. Isopropanol equivalents screening.

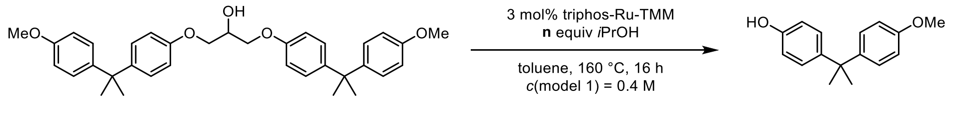

Entry	equiv iPrOH	Consumption	Me-BPA
**1 (ref)**	1	94%	85%
**2**	2	96%	83%
**3**	3	87%	85%
**4**	4	89%	73%
**5**	5	95%	75%
**6**	10	72%	47%
**7**	*i*PrOH as sole solvent	>0%	traces

The efficiency of the catalysis stagnates (with slight variations) across 5 equiv of isopropanol. Increasing the amounts of isopropanol further decreases the yields. 3 equiv of isopropanol were chosen for further experiments.

2.5 Screening of Solvents

Table S5. Solvent screening.

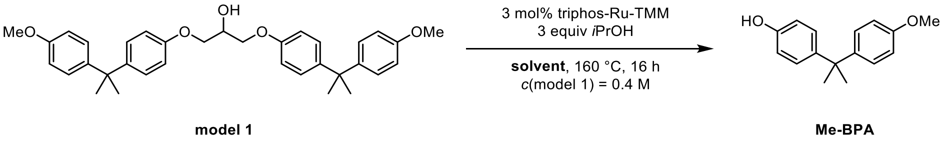

Entry	Solvent	Consumption	Me-BPA
**1 (ref)**	toluene	94%	85%
**2**	1,4-dioxane	0%	0%
**3**	1,2-dimethoxyethane	5%	11%
**4**	1,2-dichloroethane	0%	0%
**5**	*N,N*-dimethylformamide	0%	traces

2.6 Screening of Structurally Related Catalysts and Acid Co-Catalysts

Table S6. Ruthenium complex screening.

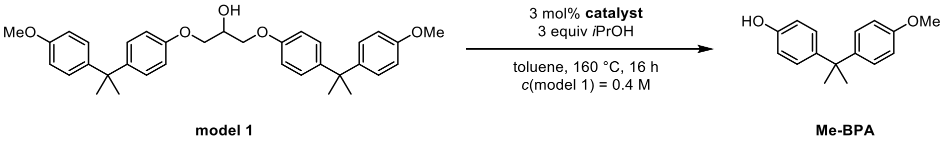

Entry	Catalyst / Other Variations	Consumption	Me-BPA
**1 (ref)**	triphos-Ru-TMM	94%	85%
**2**	N-triphos-Ru-TMM	59%	43%
**3**	triphos-Ru-H_2-CO	4%	0%
**4**	triphos-Ru-HCl-CO	5%	traces
**5**	triphos-Ru-HCl-CO / 6 mol% KOtBu	29%	9%
**6**	triphos-Ru-TMM / 3 mol% $HNTf_2$	13%	0%
**7**	triphos-Ru-TMM / 3 mol% MsOH	8%	0%
**8**	triphos-Ru-TMM / 3 mol% $HNTf_2$ / H_2 instead of *i*PrOH	4%	0%
**9**	triphos-Ru- H_2-CO / 3 mol% $HNTf_2$	13%	traces

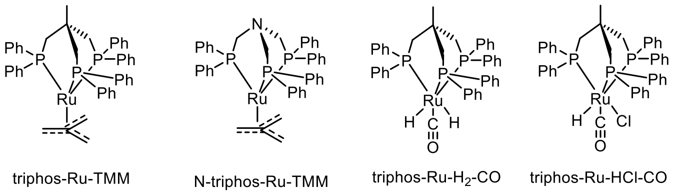

2.7 Variation of Conditions on Airstone 76E/766H and Analysis of Powders

All optimization reactions were set up in an Argon charged glovebox using a 10 ml COtube (from SyTracks) as reaction vessel and a Teflon-coated stirring bar with dried and degassed solvents. 100 mg of powderised epoxy resin were suspended in 1.0 ml of solvent, then catalyst was added. After sealing the reaction vessel, the mixtures were stirred outside of the glovebox in aluminium heating blocks. After the given reaction time, the reaction mixture was allowed to cool to room temperature, the residues taken up in acetone and transferred into a round bottom flask. Celite was added and the solvent removed *in vacuo*. The resulting mixture was loaded onto a silica gel charged column. Column chromatography using a gradient of 6/1 pentane/ethyl acetate to 4/1 pentane/ethyl acetate affords bisphenol A. Afterwards, the rest fraction is eluted from the column using 10% MeOH in DCM.

Table S7. Optimizations on Airstone 760E/766H powder.

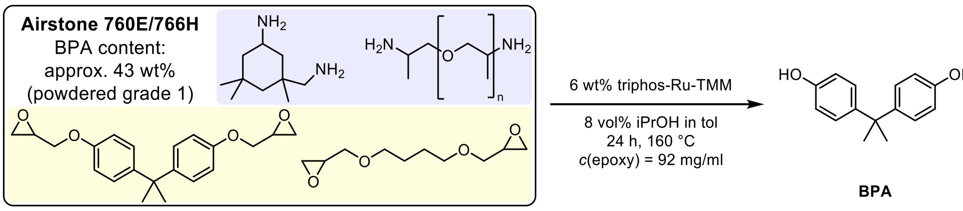

Entry	Variations	Recovered BPA	Rest Fraction	Total Mass
**1**	no catalyst added	0% (0 mg)	-	-
**2**	powder grade 3	26% (11.1 mg)	28.6 mg	40%
**3**	powder grade 2	45% (19.2 mg)	48.9 mg	68%
**4**	powder grade 1 (no variation)	56% (24.3 mg)	60.1 mg	81%
**5**	T = 140 °C	18% (7.3 mg)	10.3 mg	18%
**6**	T = 200 °C	61% (26.3 mg)	58.9 mg	85%
**7**	3 mol% triphos-Ru-TMM	34% (14.6 mg)	29.2 mg	44%
**8**	3 mol% triphos-Ru-TMM, 4 d	81% (35.0 mg)	49.4 mg	84%
**9**	3 mol% triphos-Ru-TMM, 4 d	77% (33.2 mg)	57.3 mg	91%
**10**	no isopropanol	18% (7.7 mg)	21.4 mg	29%

The cured resin was gtound into powders of different grades of coarseness, with grade 1 being the finest to grade 3 representing the most coarse-grained powder. Each of the three powders were reacted with 6 wt% catalyst under the same conditions. BPA could be isolated in all cases, with the yields correlating to particle sizes. For the more coarse-grained powder 3, 26% of BPA was isolated from the resin (entry 2), while the finest powder 1 allowed for the recovery of 56% of BPA (entry 4).

Reducing the reaction temperature to 140 °C (entry 5) or not adding isopropanol (entry 9) led to plummeting yields. Increasing the temperature to 200 °C (entry 6) only let to a marginal rise in yield of BPA. Reducing the catalyst loading by one half to 3 wt% (entry 7) reduced the amount of recovered BPA to 34%. A double entry with 3 wt% of catalyst ran over 4 days (entry 8 and 9). Over this frame of time, the amount of recovered BPA increased to 77-81%.

The influence of the particle sizes on the efficiency of the deconstruction suggests that the solvent cannot easily penetrate the highly crosslinked material. Instead, the catalysis is limited to the surface area of the resin particles suspended in solution. As determined by microscopy, powder 3 possessed average particle sizes ranging from 300–700 μm in diameter and 500 – 3000 μm in length, while the average particle sizes of powder 2 ranged from 100–350 μm in diameter and 500–3000 μm in length.

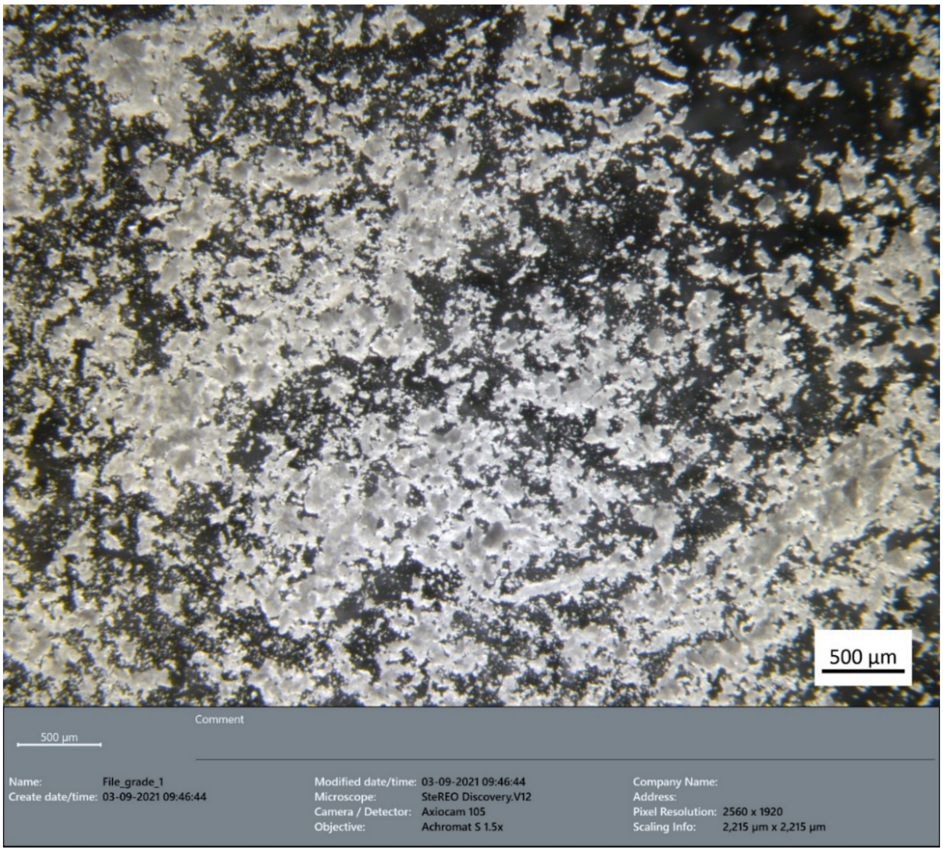

Fig. S6. Microscopic image of powder 1.

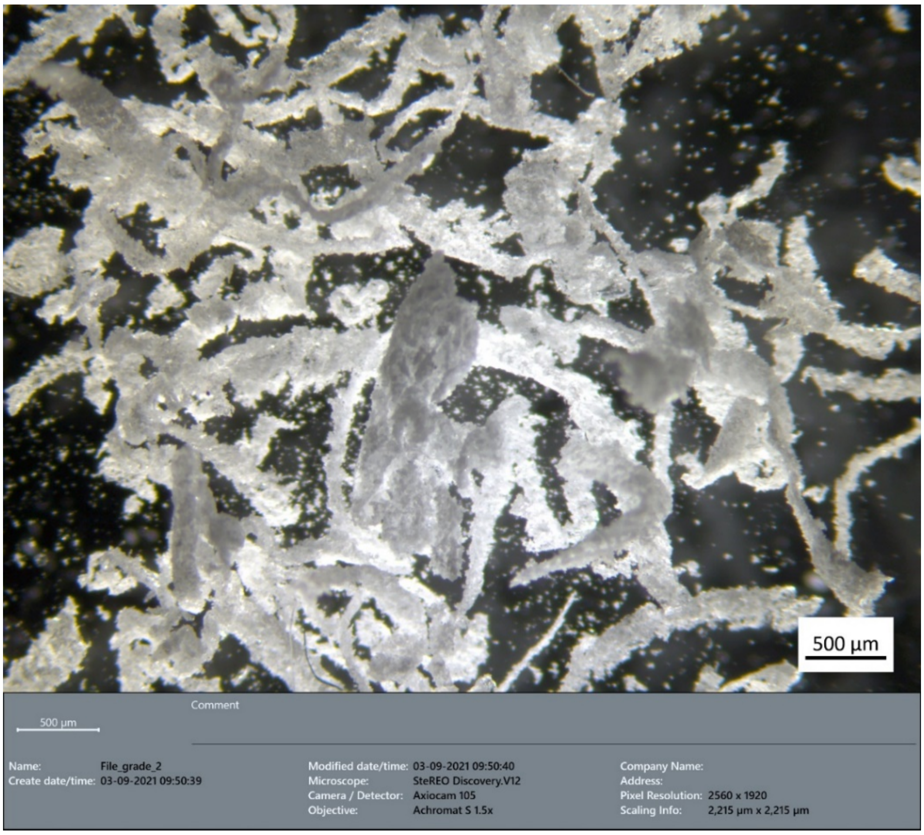

Fig. S7. Microscopic image of powder 2.

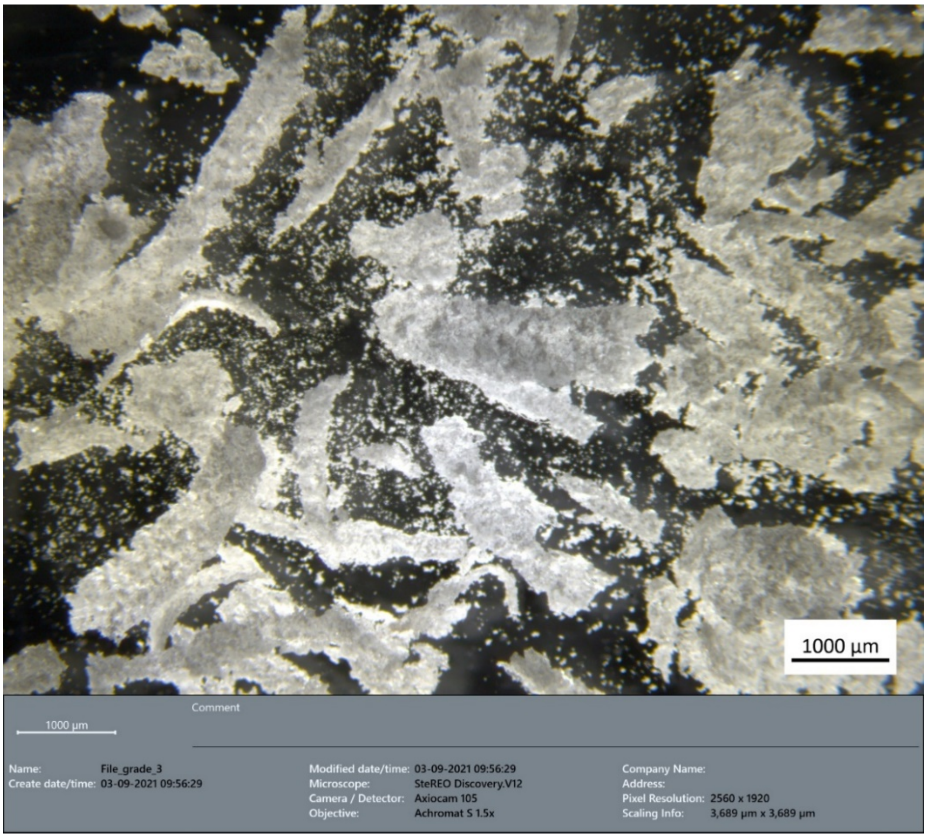

Fig. S8. Microscopic image of powder 3.

For powder 1, which gave the best result and was used for variations of reaction conditions, the particle size was analysed using dynamic light scattering, revealing that 10% of the particles have a diameter below 32.8 µm, 50% are above and 50% below 134.1 µm in diameter and 90% have a diameter below 380.5 µm.

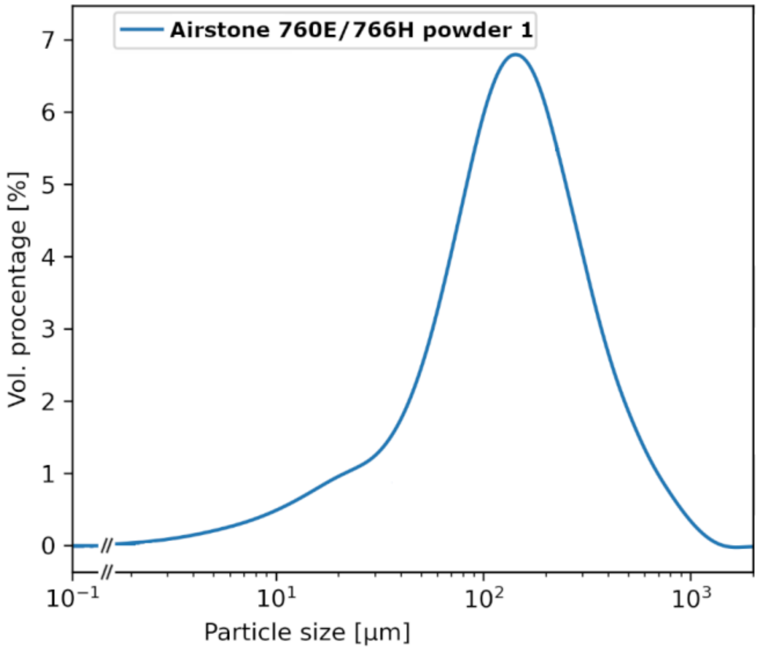

Fig. S9. Distribution of particle sizes of powder 1 as determined by dynamic light scattering.

3. Mechanistic Investigation

3.1 Kinetic Study in Model 1

In order to measure the kinetic profile of the C–O bond cleavage, **model 1** was reacted under optimized conditions nine times with different reaction times each. 43.3 mg (80.0 µmol, 1 equiv, as stock solution) of **model 1** and 0.2 ml of toluene-*d*8 were given into a 10 ml COtube in an Argon charged glovebox. 3 mol% (1.87 mg, 2.40 µmol) of triphos-Ru-TMM and 3 equiv (14.4 mg, 18.3 µl, 240 µmol) of isopropanol were added. After sealing the reaction vessel, the mixtures were stirred at 650 rpm outside of the glovebox in aluminium heating blocks at 160 °C. After the given reaction time, the reaction was cooled using a water/ice bath. Then, 1,3,5-trimethoxybenzene (as stock solution in toluene-*d*8) was added to the reaction mixture. Yields were determined by ^{1}H NMR spectroscopy of the reaction mixture with 1,3,5-trimethoxybenzene as internal standard. GC-MS was used to confirm the products detected *via* ^{1}H NMR spectroscopy for all entries.

Table S8. Kinetic investigation on **model 1**.

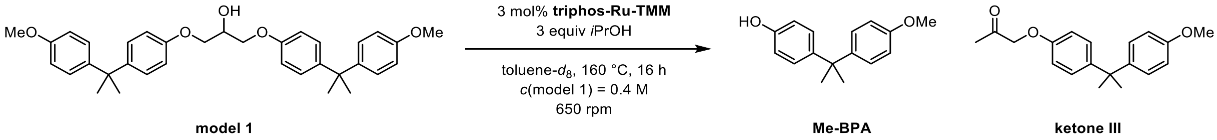

Entry	reaction time	model 1	Me-BPA	III
**1**	0 h	100%	0%	0%
**2**	2 h	95%	2%	0%
**3**	4 h	53%	50%	traces[a]
**4**	6 h	23%	76%	0%
**5**	8 h	11%	91%	0%
**6**	10 h	9%	93%	0%
**7**	12 h	0%	quant.	0%
**8**	14 h	0%	quant.	0%
**9**	16 h	0%	quant.	0%

[a] Detected *via* GC-MS but could not be detected in ^{1}H NMR spectra. Could not be quantified.

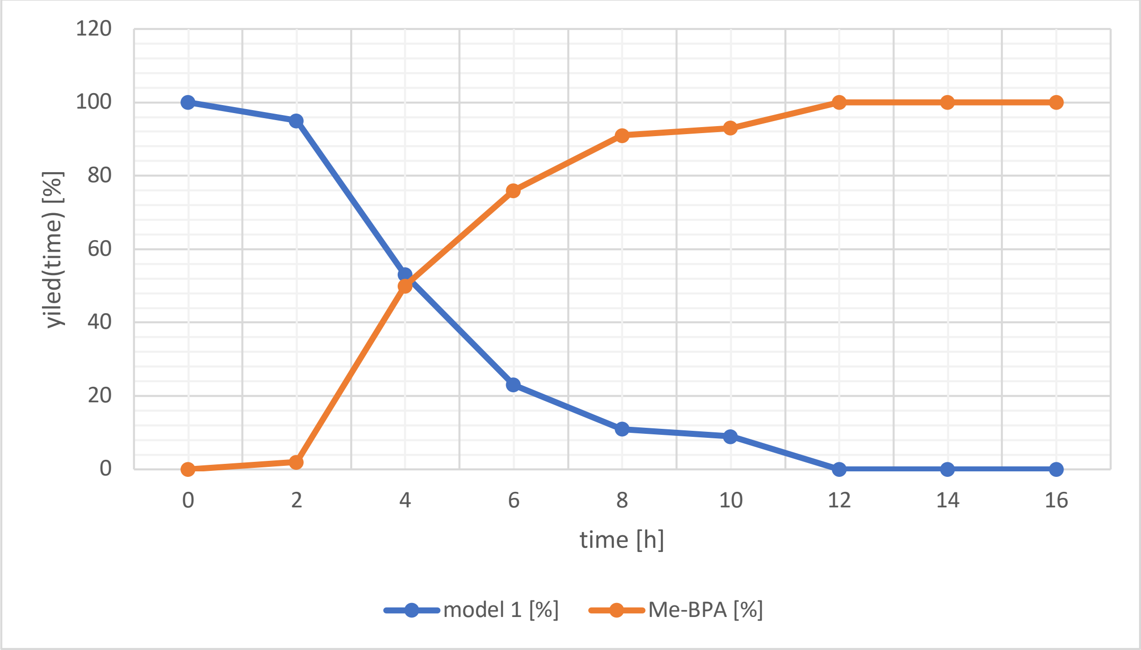

Fig. S10. Kinetic profile of C–O bond cleavage on **model 1**.

A m/z value corresponding to ketone **III**, which we propose as an intermediate in the catalytic deconstruction, was detected in trace amounts after 4 h, but could not be detected in the ^{1}H NMR spectra. In order to scrutinize whether the detected compound is ketone **III**, said compound was synthesised and the GC trace and mass spectra compared, revealing a match.

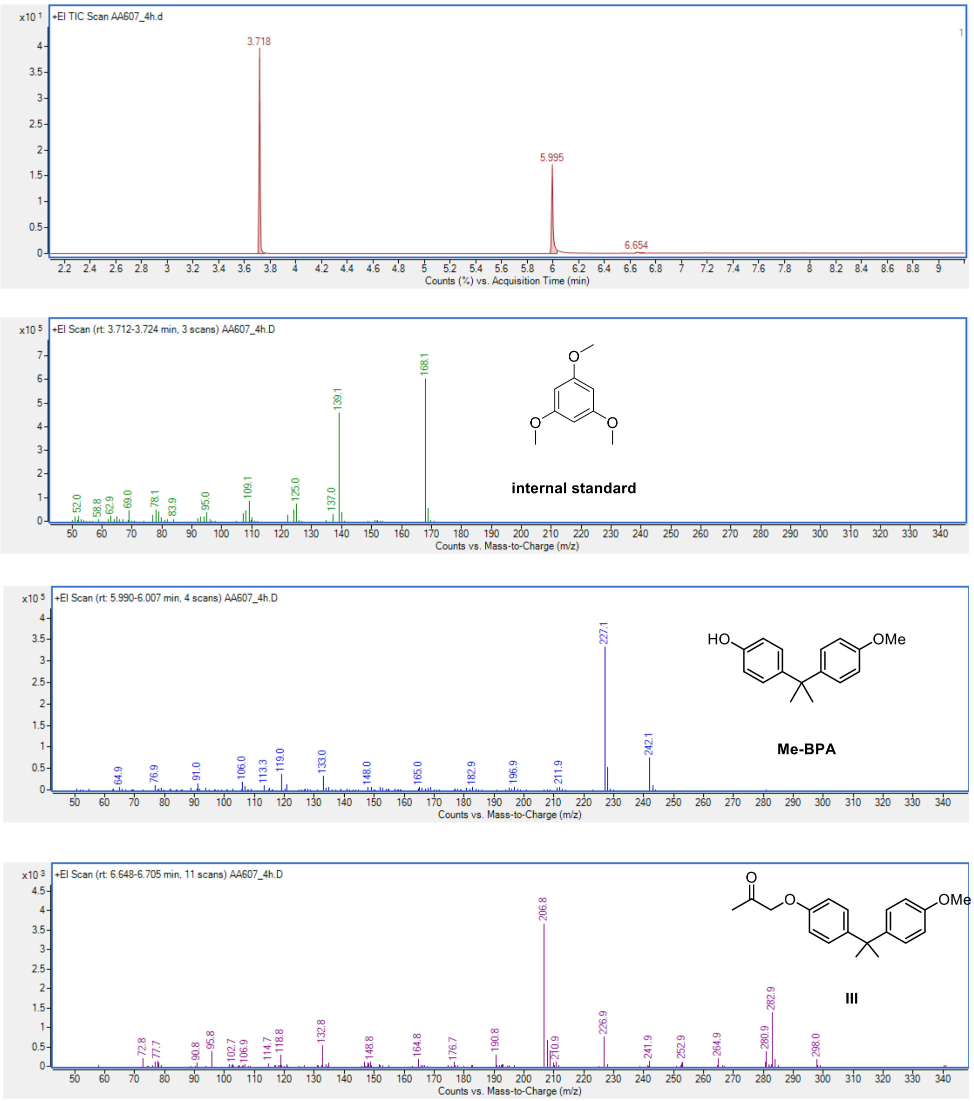

Fig. S11. GC-MS of catalytic deconstruction of **model 1** after 4 h.

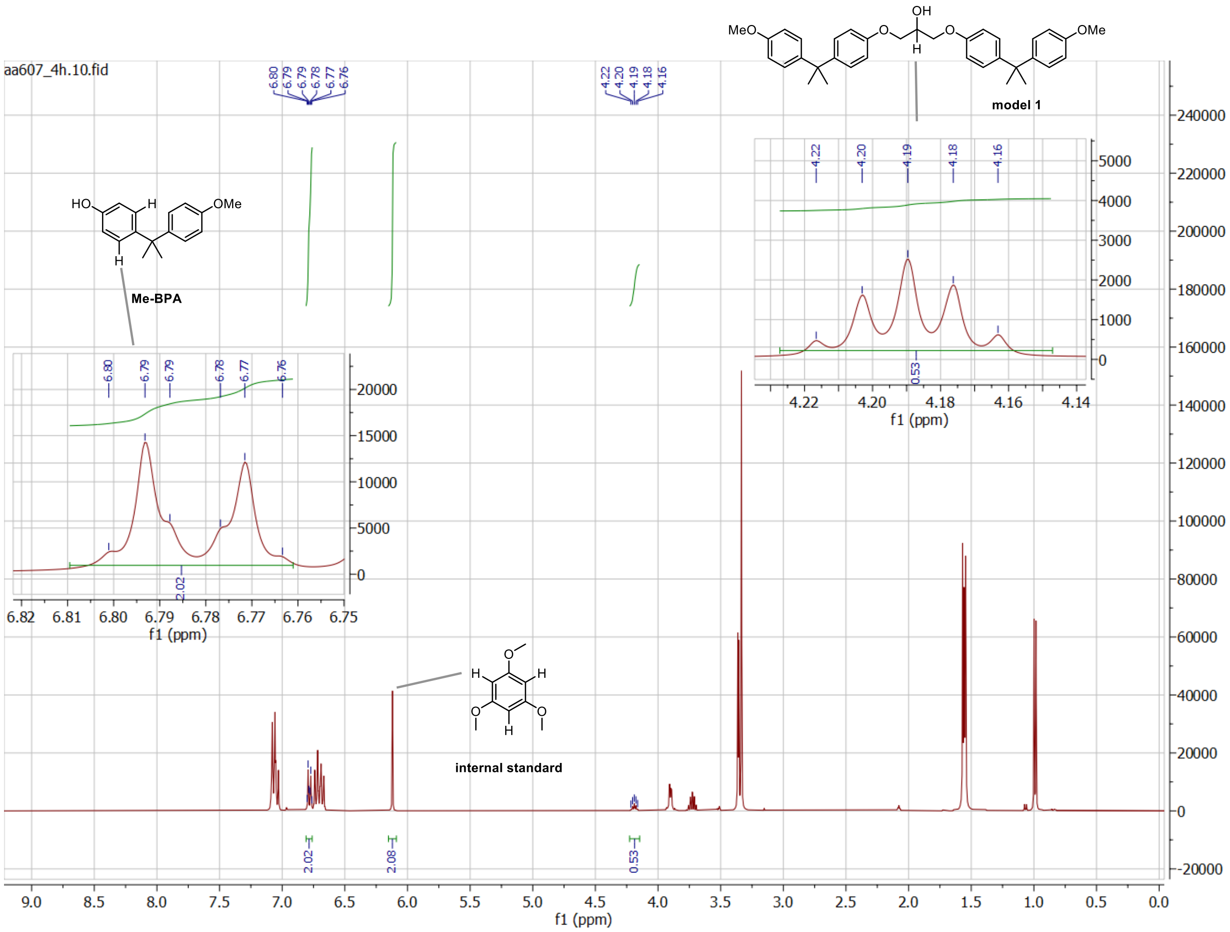

Fig. S12. ^{1}H NMR spectra of catalytic deconstruction of **model 1** after 4 h with monitored protons and their corresponding peaks highlighted.

In order to scrutinize whether **ketone III** is a potential intermediate in the catalysis, it was used as a substrate in a one-to-one ration with **model 1** under standard reaction conditions. The reaction was set up in a J Young NMR tube and operando monitored using ^{1}H and ^{31}P NMR spectroscopy.

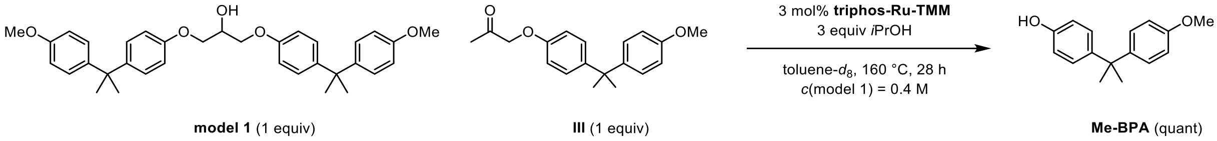

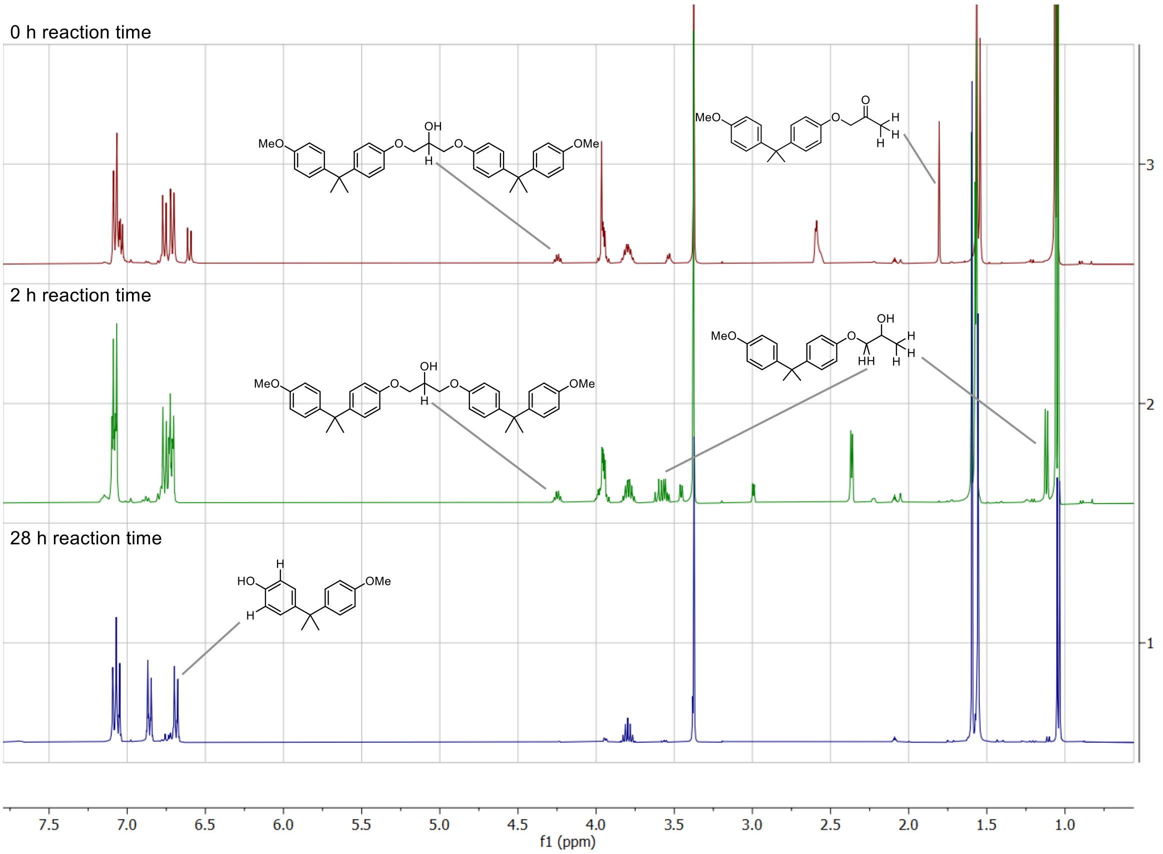

Within the first two hours, the major part of ketone **III** is transfer hydrogenated to the corresponding alcohol (alcohol **III**), due to triphos-Ru-TMM still being present and active for trans hydrogenations as shown in sections 3.2 and 3.3. Over the course of the next 28 h, the catalyst activation takes place and both **model 1** and the alcohol formed from ketone **III** are consumed, forming Me-BPA quantitatively.

3.2 Detection of Acetone as Corresponding Disconnection Product

In order to detect acetone as disconnection product, **model 1** was reacted under optimized conditions with a different hydrogen source than isopropanol. 43.3 mg (80.0 µmol, 1 equiv, as stock solution) of **model 1** and 0.2 ml of toluene-d_8 were given into a 10 ml COtube in an Argon charged glovebox. 3 mol% (1.87 mg, 2.40 µmol) of triphos-Ru-TMM and 1 equiv (9.77 mg, 9.68 µl, 80.0 µmol) of 1-phenylethanol were added. After sealing the reaction vessel, the mixtures were stirred at 650 rpm outside of the glovebox in aluminium heating blocks at 160 °C for 16 h. The reaction was then cooled using a water/ice bath, and the reaction mixture analysed using ^1H NMR and ^{13}C NMR spectroscopy using 1,3,5-trimethoxybenzene as internal standard and toluene-d_8 as solvent.

a) ^1H NMR spectra

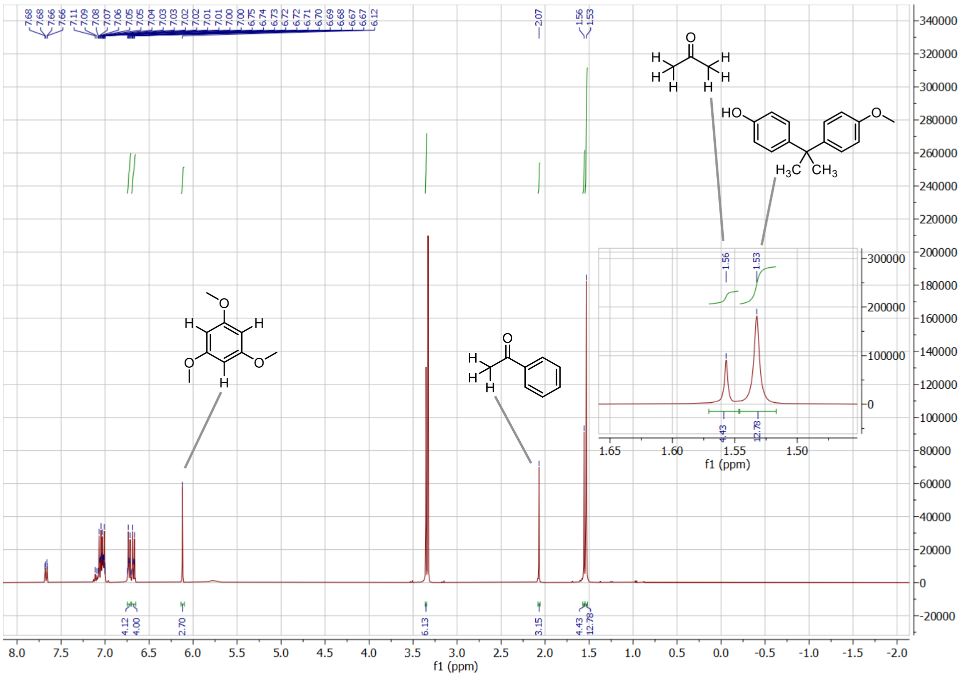

b) ^{13}C NMR spectra

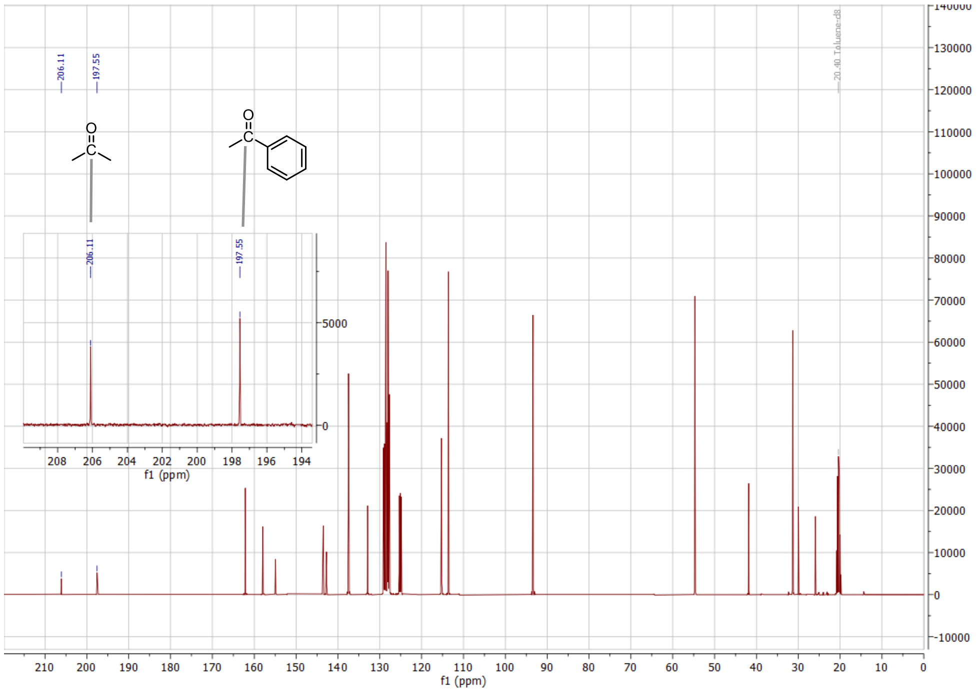

3.3 Activation of Precatalyst - Operando Monitoring *via* NMR Spectroscopy

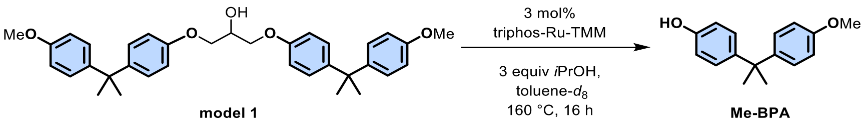

a)^{1}H NMR spectra

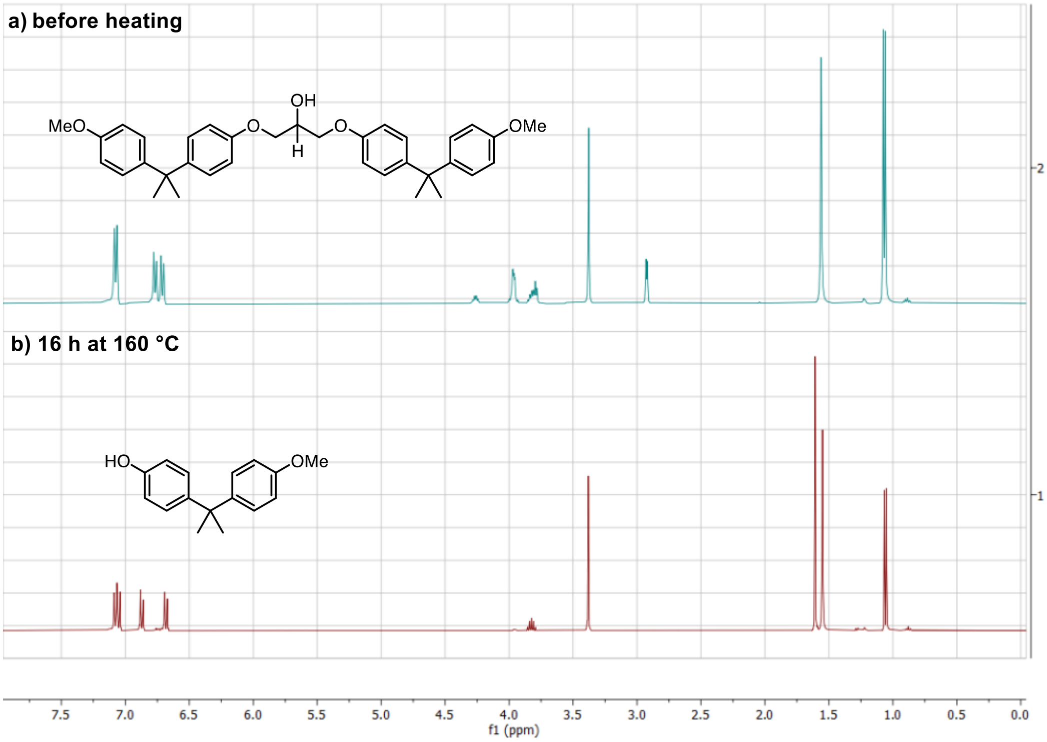

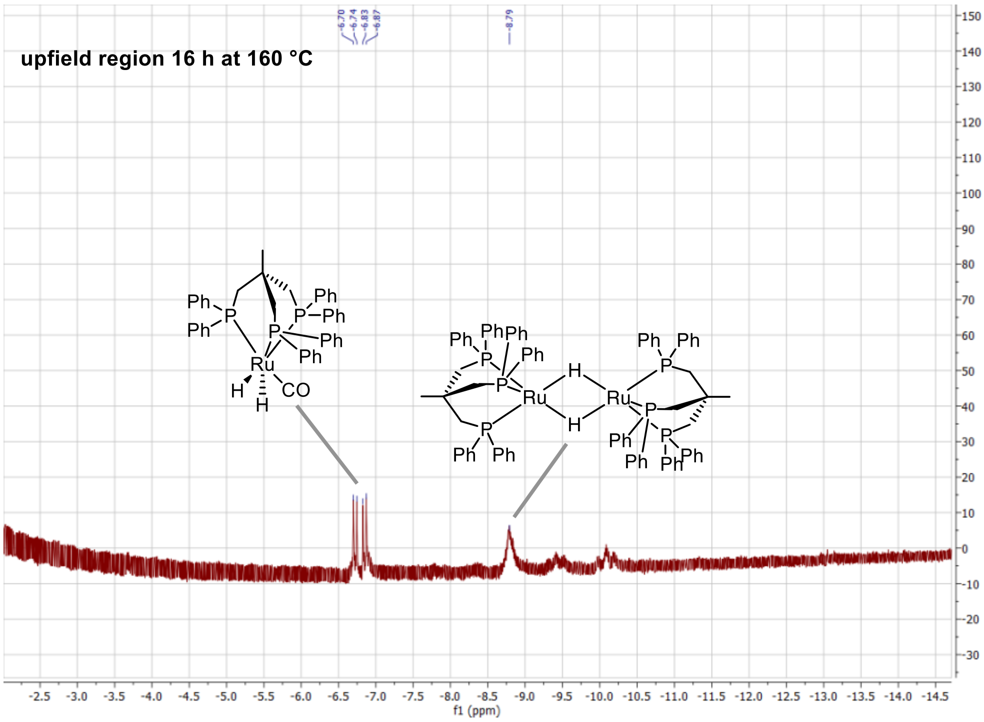

b) ^{31}P NMR spectra

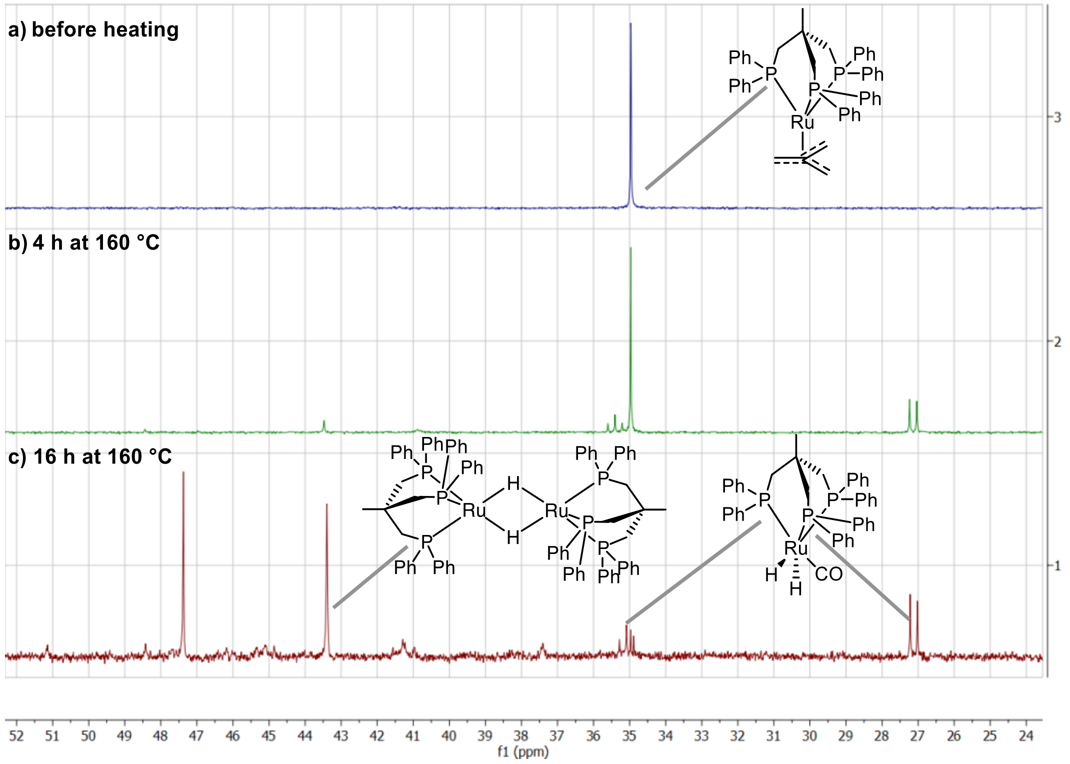

3.6 Identification of triphos-Ru-H_2-CO in Reaction Mixture

a) Zoomed in ^{1}H NMR spectra

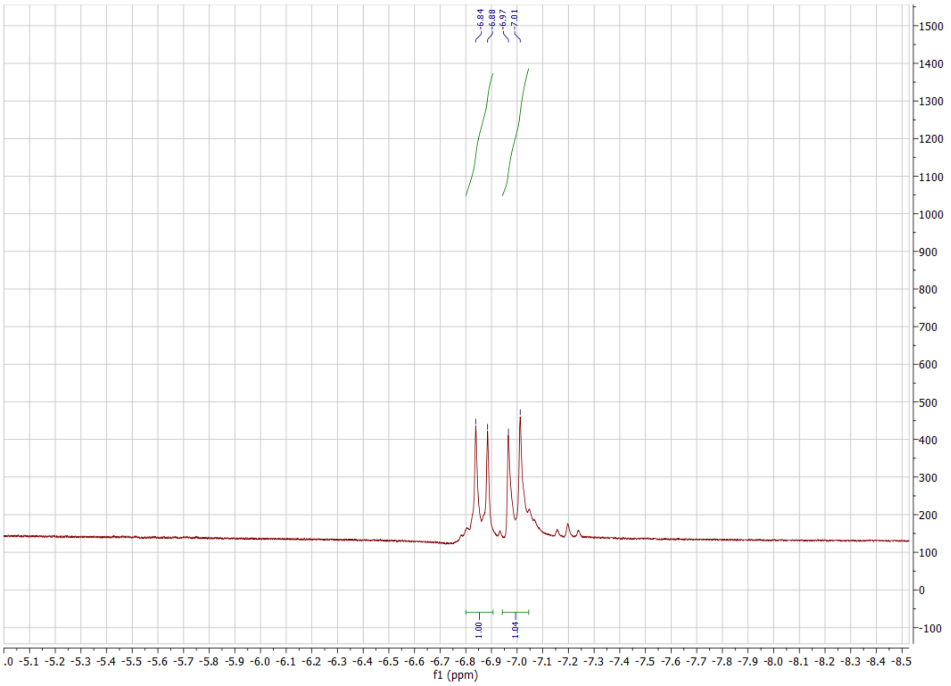

b) Zoomed in ^{31}P NMR spectra

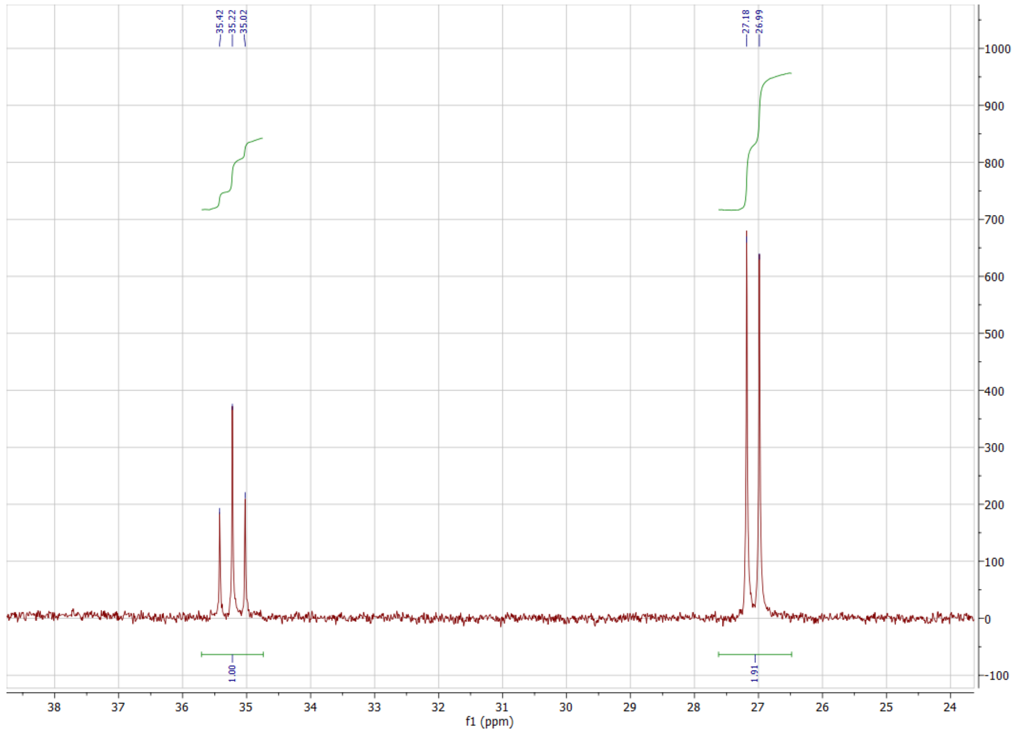

c) ^{1}H, ^{31}P HSQC NMR spectra

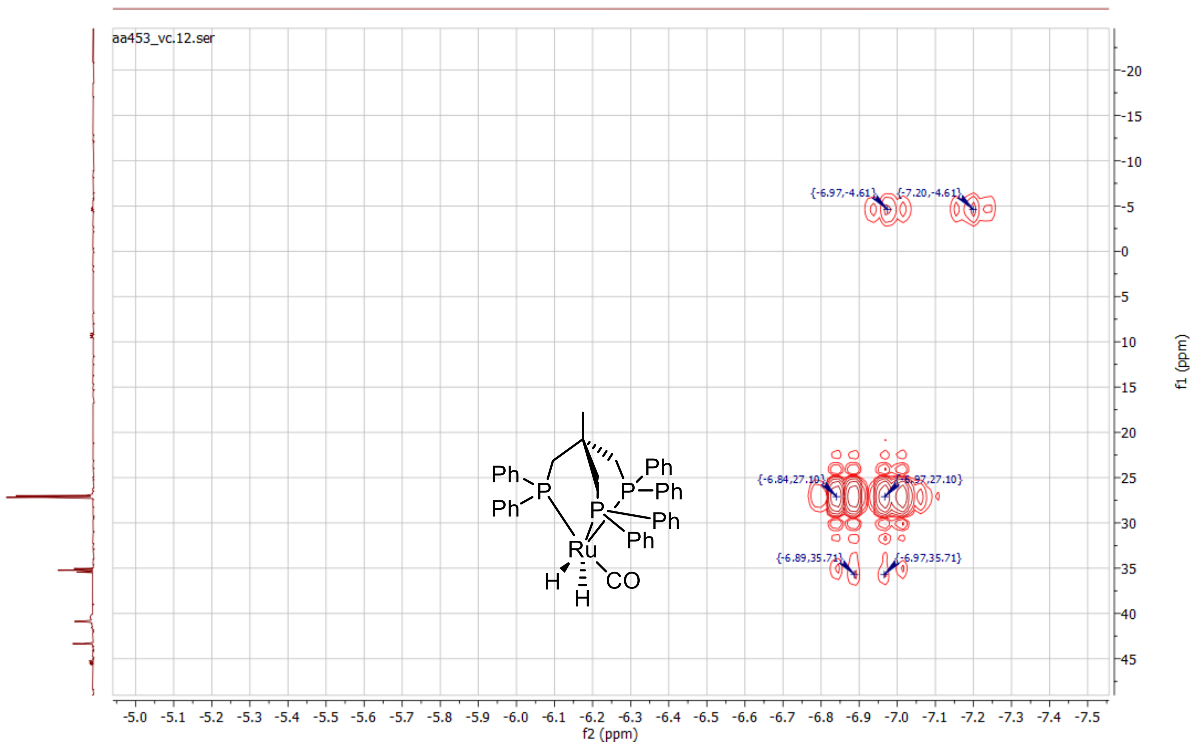

3.4 Hydride-Bridged Binuclear Ruthenium(I) Complex

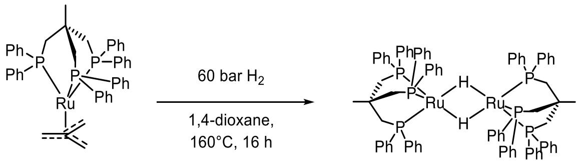

[triphos-Ru-H]2 was prepared according to a modified reported procedure[9]. In an Argon-charged glovebox 15.0 mg (19.0 µmol, 1.0 equiv) of triphos-Ru-TMM was weighed into an 8 ml glass vial and 1.5 ml of 1,4-dioxane were added, forming a suspension. The open vial was placed in 20 ml steel autoclave, sealed and pressurised with 60 bar of hydrogen. The reaction mixture was stirred for 16 h at 160 °C and then allowed to cool to rt. The pressure was carefully released, and the autoclave was opened in an Argon charged glovebox. The clear solution was decanted off the orange precipitate, which was then washed two times with 1 ml of THF and dried *in vacuo*, affording the title complex as orange to red powder in a yield of 51% (7.1 mg, 4.89 µmol).

^{1}H NMR (CD_2Cl_2, 400 MHz, 25 °C): δ = 7.20 (bs, 24H), 7.13 (t, *J* = 7.4 Hz, 12H), 6.76 (t, *J* = 7.6 Hz, 24H), 2.26 (s, 12H), 1.53 (s, 6H), -8.85 (bs, 2H) ppm; ^{31}P NMR (CD_2Cl_2, 162 MHz, 25 °C): δ = 42.9 (s, 6P) ppm. The NMR spectra are in agreement with reported data[9].

Test on **model 1**:

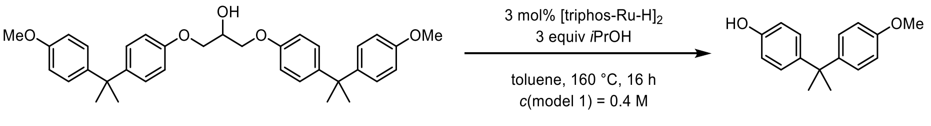

model 1

Me-BPA (0%)

no conversion observed

4. Synthesis of Compounds

4.1 Complexes

triphos-Ru-TMM

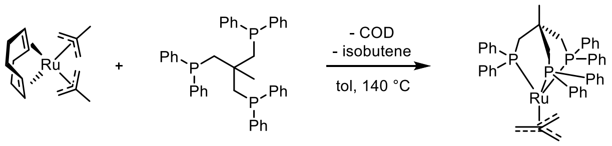

triphos-Ru-TMM was prepared according to a modified reported procedure[4]. In an Argon-charged glovebox 128 mg (0.40 mmol, 1.0 equiv) of (COD)Ru(2-methylallyl)$_2$ and 250 mg (0.40 mmol, 1.0 equiv) of 1,1,1 tris(diphenylphosphinomethyl)ethane (triphos) mixed into a 10 ml COtube and 3 ml of toluene were added. The reaction mixture was stirred at 140 °C over-night in a metal block, forming a grey to yellow suspension. After letting the reaction cool to room temperature, 3 ml of pentane were added and the reaction mixture cooled to -30 °C in a freezer. Afterwards, the solution was decanted off and the precipitate washed two times with 2 ml of pentane. The yellow solid was dried *in vacuo* in order to remove residual solvents and then taken up in 8 ml of DCM. This yellow solution was filtered through a PTFE syringe filter into a fresh vial, overlayered with 16 ml of pentane and left to crystallise in a freezer at -30 °C. The mother liquor was decanted off and the off-white to colourless crystals washed with 2 ml of pentane. The product was dried *in vacuo*. A second crop of crystals was obtained in the previously described manner from the mother liquor, affording 193 mg (0.24 mmol, 62%) of the desired complex in total.

^{1}H NMR (CD$_2$Cl$_2$, 400 MHz, 25 °C): δ = 7.12 – 7.01 (m, 18H), 6.95 - 6.91 (m, 12H), 2.24 - 2.22 (m, 6H), 1.62 (s, 6H), 1.39 (s, 3H) ppm; ^{31}P NMR (CD$_2$Cl$_2$, 162 MHz, 25 °C): δ = 34.5 ppm. The NMR spectra are in agreement with reported data[4].

triphos-Ru-HCl-CO

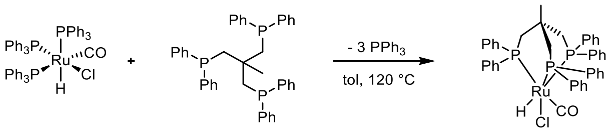

triphos-Ru-HCl-CO was prepared according to a modified reported procedure[10]. In an Argon-charged glovebox 21.4 mg (0.05 mmol, 1.0 equiv) of (PPh$_3$)$_3$RuHCl(CO) and 31.2 mg (0.05 mmol, 1.0 equiv) of 1,1,1 tris(diphenylphosphinomethyl)ethane (triphos) were given into a 10 ml COtube and 1 ml of toluene were added. The reaction mixture was stirred at 120 °C over-night in a metal block, forming a yellow suspension. After cooling to room temperature, the suspension was given onto a basic aluminium oxide pipette column and the precipitate was washed with three times 1 ml of toluene. Afterwards, the product was eluted with four times 1 ml of DCM. Recrystallization from DCM by over layering with toluene afforded triphos-Ru-HCl-CO as pale-yellow solid containing minor unidentified impurities in a yield of 59% (23.1 mg, 29.0 µmol). The complex was tested without further purification.

^{1}H NMR (CD$_2$Cl$_2$, 400 MHz, 25 °C): δ = 7.83 – 7.64 (m, 6H), 7.60 – 7.54 (m, 2H), 7.46 – 7.27 (m, 5H), 7.27 – 7.02 (m, 9H), 7.03 – 6.86 (m, 6H), 6.75 – 6.69 (m, 2H), 2.66 – 2.52 (m, 1H), 2.39 – 2.13 (m, 5H), 1.52 (d, *J* = 2.8 Hz, 3H), -5.91 (ddd, *J* = 93.9, 19.2, 15.1 Hz, 1H) ppm; ^{31}P NMR (CD$_2$Cl$_2$, 162 MHz, 25 °C): δ = 48.13 (dd, *J* = 39.8, 17.6 Hz), 13.10 (dd, *J* = 39.9, 32.0 Hz), 0.32 (ddd, *J* = 32.1, 17.5, 6.6 Hz) ppm.

triphos-Ru-H2-CO

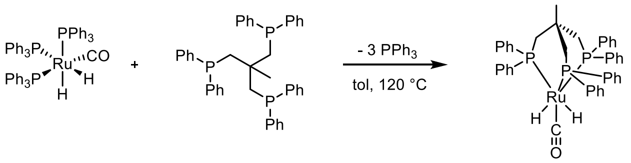

triphos-Ru-H$_2$-CO was prepared according to a modified reported procedure[11]. In an Argon-charged glovebox 45.8 mg (0.05 mmol, 1.0 equiv) of (PPh$_3$)$_3$RuH$_2$(CO) and 31.2 mg (0.05 mmol, 1.0 equiv) of 1,1,1 tris(diphenylphosphinomethyl)ethane (triphos) were given into a 10 ml COtube and 0.6 ml of toluene were added. The reaction mixture was stirred at 120 °C over-night in a metal block, forming a yellow suspension. After letting the reaction cool to room temperature, 3 ml of diethyl ether were added and the reaction mixture cooled to -30 °C in a freezer. Afterwards, the solution was decanted off and the precipitate washed two times 0.5 ml of diethyl ether. The yellow solid was taken up in 2 ml of DCM. This yellow solution was filtered through a PTFE syringe filter into a fresh vial, overlayered with 4 ml of pentane and left to crystallise in a freezer at -30 °C. The mother liquor was decanted off and the yellow powder dried *in vacuo*, affording the product in a yield of 73% (27.4 mg, 36.0 µmol).

^{1}H NMR (CDCl$_3$, 400 MHz, 25 °C): δ = 7.72 – 7.57 (m, 6H), 7.31 – 7.26 (m, 11H), 7.20 – 6.90 (m, 13H), 2.27 – 2.07 (m, 6H), 1.48 (d, J = 2.7 Hz, 3H), -7.33 (dd, *J* = 49.9, 18.6 Hz, 2H) ppm; ^{31}P NMR (CD$_2$Cl$_2$, 162 MHz, 25 °C): δ = 34.2 (t, *J* = 32.3 Hz, 1P), 26.6 (d, *J* = 32.4 Hz, 2P) ppm. The NMR spectra are in agreement with reported data[12].

N-triphos

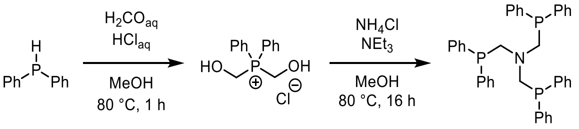

N-triphos was prepared according to a modified reported procedure[13]. In an Argon-charged glovebox 2.30 ml (2.46 g, 13.2 mmol, 1.0 equiv) of HPPh$_2$ were dissolve in 10 ml MeOH in a 20 ml screw cap vial. 1.81 ml (2.64 g, 31.7 mmol, 2.4 equiv) of formaldehyde (36% solution in water, degassed) and 1.27 ml (1.47 g, 14.5 mmol, 1.1 equiv) of hydrogen chloride (37% in water, degassed) were added and the vial sealed. The reaction mixture was stirred at 80 °C in an oil bath outside of the glovebox for 1 h and then cooled to room temperature. The vial was open under air and the solution filtered through a PTFE syringe filter into a fresh vial. The solvent was removed *in vacuo* yielding the crude product as a colourless solid. The solid was recrystallised from hot methanol in order to remove impurities, yielding [Ph$_2$P(CH$_2$OH)$_2$]Cl as colourless crystals in a yield of 67% (2.25 g, 13.2 mmol).

^{1}H NMR (CDCl$_3$, 400 MHz, 25 °C): δ = 7.87 – 7.75 (m, 3H), 7.67 – 7.62 (m, 2H), 5.06 (s, 2H) ppm; ^{31}P NMR (CD$_2$Cl$_2$, 162 MHz, 25 °C): δ = 13.3 (s, 1P) ppm. The NMR spectra are in agreement with reported data[14].

In an Argon-charged glovebox 1.14 g (4.5 mmol, 3.0 equiv) of [Ph$_2$P(CH$_2$OH)$_2$]Cl were dissolved in 5 ml MeOH in a 12 ml screw cap vial. 80.2 mg (1.5 mmol, 1 equiv) of ammonium chloride and 2.09 ml (1.52 g, 15 mmol, 10 equiv) of triethylamine were added and the vial sealed. The reaction mixture was stirred at 80 °C in an oil bath outside of the glovebox over-night, forming a white precipitate, and then cooled to room temperature. In an Argon-charged glovebox, the solution was decanted and the precipitate washed with 2 ml MeOH. The crude product was recrystallised from 4 ml hot methanol / THF 1:1, yielding N-triphos as colourless crystals in a yield of 88% (817 mg, 1.50 mmol).

^{1}H NMR (CDCl$_3$, 400 MHz, 25 °C): δ = 7.30 - 7.26 (m, 12H), 7.20 - 7.12 (m, 18H), 3.72 (d, J = 3.5 Hz, 6H) ppm; ^{31}P NMR (CD$_2$Cl$_2$, 162 MHz, 25 °C): δ = - 29.0 (s, 1P) ppm. The NMR spectra are in agreement with reported data[13].

N-triphos-Ru-TMM

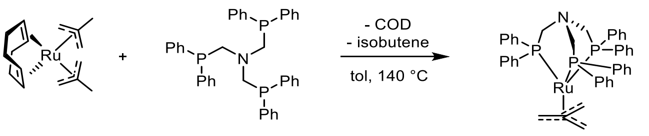

N-triphos-Ru-TMM was prepared according to the procedure used for triphos-Ru-TMM. In an Argon-charged glovebox 39.0 mg (0.125 mmol, 1.0 equiv) of (COD)Ru(2-methylallyl)$_2$ and 76.5 mg (0.125 mmol, 1.0 equiv) of N-triphos were given into a 10 ml COtube and 1 ml of toluene were added. The reaction mixture was stirred at 140 °C over-night in a metal block, forming a grey to yellow suspension. After letting the reaction cool to room temperature, 1 ml of pentane was added and the reaction mixture cooled to -30 °C in a freezer. Afterwards, the solution was decanted off and the precipitate rinsed with two times 2 ml of pentane and then washed with 3 times with 3 ml of DCM, affording 45.6 mg (590 µmol, 48%) of the desired complex.

^{1}H NMR (CD_2Cl_2, 400 MHz, 25 °C): δ = 7.11 - 7.07 (m, 8H), 7.04 – 6.87 (m, 22H), 3.87 (s, 6H), 1.66 (s, 6H) ppm; ^{31}P NMR (CD_2Cl_2, 162 MHz, 25 °C): δ = 17.8 ppm. The NMR spectra are in agreement with reported data[15].

4.2 Model Substrates

Me-BPA (4-(2-(4-methoxyphenyl)propan-2-yl)phenol)

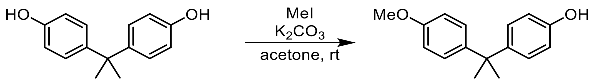

11.4 g (50.0 mmol, 1.0 equiv) of bisphenol A were dissolved in 100 ml of acetone in a 250 ml round bottom flask under air. Under stirring, 10.4 g (75.0 mmol, 1.5 equiv) of potassium carbonate were added, forming a suspension. Then, 3.11 ml (7.10 g, 50.0 mmol, 1 equiv) of methyl iodide were added. The reaction mixture was stirred over-night at room temperature. Afterwards, the suspension was filtered over a plug of silica. The solvent was removed *in vacuo*. Column chromatography over silica gel using a gradient of 15/1 pentane/ethyl acetate to 10/1 pentane/ethyl acetate afforded Me-BPA as colourless highly viscous oil in a yield of 53% (6.40 g, 52.8 mmol).

R_f (pentane/ethyl acetate 4/1, silica gel) = 0.3; ^{1}H NMR ($CDCl_3$, 400 MHz, 25 °C): δ = 7.16 -7.14 (m, 2H), 7.11 - 7.09 (m, 2H), 6.83 - 6.81 (m, 2H), 6.74- 6.72 (m, 2H), 4.76 (s, 1H), 3.80 (s, 3H), 1.64 (s, 6H) ppm. The NMR spectra are in agreement with reported data[16].

Model 1 (1,3-bis(4-(2-(4-methoxyphenyl)propan-2-yl)phenoxy)propan-2-ol)

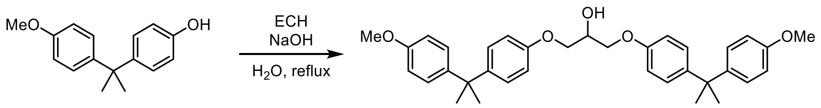

model 1

1.43 g (5.90 mmol, 2.0 equiv) of Me-BPA were suspended in 50 ml of water in a 100 ml round bottom flask under air. 236 mg (5.90 mmol, 2 equiv) of sodium hydroxide were added and the mixture was stirred at room temperature for 10 min. Then, 231 µl (273 mg, 2.95 mmol, 1 equiv) of epichlorohydrin (ECH) were added. The flask was equipped with a reflux condenser and the mixture was heated to reflux over-night under vigorous stirring. The reaction mixture was allowed to cool to room temperature and 30 ml of DCM were added. The crude product was extracted using three times 30 ml of DCM. The combined organic phases were dried over $MgSO_4$, filtered, and the solvent removed *in vacuo*. Column chromatography over silica gel using a gradient of 10/1 pentane/ethyl acetate to 5/1 pentane/ethyl acetate, afforded model 1 as colourless highly viscous oil in a yield of 66% (1.05 g, 1.94 mmol). Some but not all batches of model 1 crystallised slowly over the course of days to weeks.

R_f (pentane/ethyl acetate 3/1, silica gel) = 0.41; ^{1}H NMR ($CDCl_3$, 400 MHz, 25 °C): δ = 7.18 - 7.15 (m, 8H), 6.86 - 6.82 (m, 8H), 4.38 (h, *J* = 5.3 Hz, 1H), 4.19 – 4.08 (m, 4H), 3.80 (s, 6H), 2.65 (d, *J* = 5.3 Hz, 1H), 1.66 (s, 12H) ppm; ^{13}C NMR ($CDCl_3$, 101 MHz, 25 °C): δ = 157.5 (s, 2C), 156.3 (s, 2C), 143.9 (s, 4C), 143.1 (s, 2C), 127.9 (s,4C), 127.8 (d, 4C), 114.0 (d, 4C), 113.4 (d, 4C), 68.9 (d, 1C), 68.7 (t, 2C),

55.3 (q, 2C), 41.8 (s, 2C), 31.2 (q, 4C) ppm; HRMS (ESI+): calculated $[M+Na]^+$ = $[C_{35}H_{40}O_5+Na]^+$ 563.2768; found 563.2762.

Model 2 (1-((cyclohexylmethyl)amino)-3-(4-(2-(4-methoxyphenyl)propan-2-yl)phenoxy)propan-2-ol)

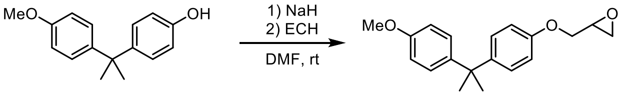

1.70 g (7.02 mmol, 1.0 equiv) of Me-BPA were dissolved in 10 ml of dry DMF in a flame-dried round bottom flask purged with argon. The reaction mixture was cooled with a water / ice bath, and 310 mg (7.72 mmol, 1.1 equiv, 60% in mineral oil) of sodium hydride were added in portions. After complete addition, the mixture was left to stir for another 15 min. Then, while still cooling with a water / ice bath, 2.75 ml (3.25 g, 35.1 mmol, 5.0 equiv) of epichlorohydrine were added. The cooling bath was removed, and the reaction mixture stirred at room temperature over-night. 60 ml of water and 30 ml of ethyl acetate were added subsequently, and the combined organic phase was extracted with three times 30 ml of ethyl acetate, washed with brine, dried over $MgSO_4$, filtered, and the solvent was removed *in vacuo*. Column chromatography over silica gel using 10/1 pentane/ethyl acetate afforded 2-((4-(2-(4-methoxyphenyl)propan-2-yl)phenoxy)methyl)oxirane as colourless oil in a yield of 94% (1.96 g, 7.02 mmol).

R_f (pentane/ethyl acetate 4/1, silica gel) = 0.6; ^{1}H NMR ($CDCl_3$, 400 MHz, 25 °C): δ = 7.15 – 7.12 (m, 4H), 6.83 – 6.79 (m, 4H), 4.18 (dd, *J* = 11fz.0, 3.3 Hz, 1H), 3.95 (dd, *J* = 11.0, 5.6 Hz, 1H), 3.78 (s, 3H), 3.36 – 3.32 (m, 1H), 2.90 (dd, *J* = 5.0, 4.1 Hz, 1H), 2.74 (dd, *J* = 4.9, 2.6 Hz, 1H), 1.63 (s, 6H) ppm; ^{13}C NMR ($CDCl_3$, 101 MHz, 25 °C): δ = 157.5 (s, 1C), 156.4 (s, 1C), 143.8 (s, 1C), 143.1 (s, 1C), 127.9 (d, 2C), 127.8 (d 2C), 114.1 (d, 2C), 113.3 (d, 2fC), 68.8 (t, 1C), 55.3 (q, 1C), 50.2 (s, 1C), 44.9 (t, 1C), 41.8 (s, 1C), 31.1 (q, 2C) ppm; HRMS (ESI+): calculated $[M+Na]^+$ = $[C_{19}H_{22}O_3+Na]^+$ 321.1461; found 321.1460.

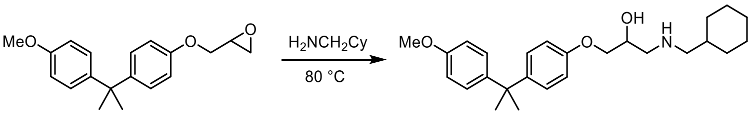

model 2

In a round bottom flask, 321 mg (1.08 mmol, 1 equiv) of 2-((4-(2-(4-methoxyphenyl)propan-2-yl)phenoxy)methyl)oxirane were mixed with 1.40 ml (1.61 g, 10.8 mmol, 10 equiv) of cyclohexanemethylamine and stirred at 80 °C for 6 h, then cooled to room temperature. The residue was taken up in 25 ml of ethyl acetate and washed five times with 15 ml of brine, dried over $MgSO_4$ and filtered. The solvent volume was reduced to a few ml *in vacuo,* overlayered with pentane and then stored in a freezer at -30 °C. The product precipitated as colourless solid over the course of a few days. The mother liquor was decanted off and the solid washed with pentane, affording the product in a yield of 47% (206 mg, 0.50 mmol).

^{1}H NMR ($CDCl_3$, 400 MHz, 25 °C): δ = 7.18 – 7.10 (m, 4H), 6.84 – 6.77 (m, 4H), 4.04 – 3.97 (m, 1H), 3.96 – 3.94 (m, 2H), 3.78 (s, 3H), 2.87 – 2.69 (m, 2H), 2.53 – 2.41 (m, 2H), 1.80 – 1.66 (m, 5H), 1.63 (s, 6H), 1.50 – 1.38 (m, 1H), 1.29 – 1.13 (m, 3H), 0.97 – 0.84 (m, 2H) ppm; ^{13}C NMR ($CDCl_3$, 101 MHz, 25 °C): δ = 157.5 (s, 1C), 156.6 (s, 1C), 143.6 (s, 1C), 143.2 (s, 1C), 127.9 (d, 2C), 127.8 (d, 2C), 114.0 (d, 2C), 113.4 (d, 2C), 70.6 (t, 1C), 68.2 (q, 1C), 56.8 (t, 1C), 55.3 (q, 1C), 52.0 (t, 1C) 41.8 (s, 1C), 38.2 (d, 1C), 31.5, (t, 2C), 31.2 (q, 2C), 26.8 (t, 1C), 26.2 (t, 2C) ppm; HRMS (ESI+): calculated $[M+H]^+$ = $[C_{26}H_{38}NO_3+H]^+$ 412.2846; found 412.2866.

Model 3 (1-(diethylamino)-3-(4-(2-(4-methoxyphenyl)propan-2-yl)phenoxy)propan-2-ol)

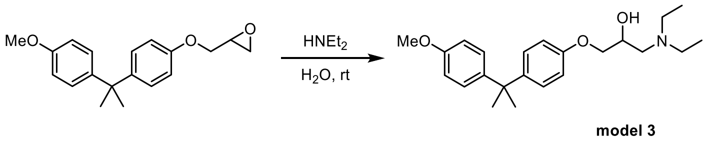

In a round bottom flask, 1.79 g (6 mmol, 1 equiv) of 2-((4-(2-(4-methoxyphenyl)propan-2-yl)phenoxy)methyl)oxirane were suspended in 4 ml of water. 0.75 ml (527 mg, 7.2 mmol, 1.2 equiv) of diethylamine and was added and the reaction mixture stirred vigorously at rt over. 40 ml of water and 30 ml of ethyl acetate were added subsequently, and the combined organic phase was extracted with three times 30 ml of ethyl acetate, washed with brine, dried over $MgSO_4$, filtered, and the solvent was removed *in vacuo*. Column chromatography over silica gel using a gradient from ethyl acetate 80% / methanol 20% to ethyl acetate 40% / methanol 60% afforded 1-(diethylamino)-3-(4-(2-(4-methoxyphenyl)propan-2-yl)phenoxy)propan-2-ol as colourless oil in a yield of 51% (1.14 g, 3.07 mmol).

R_f (ethyl acetate, silica gel) = 0;13 ^{1}H NMR ($CDCl_3$, 400 MHz, 25 °C): δ = 7.21 – 7.09 (m, 4H), 6.89 – 6.77 (m, 4H), 4.04 – 3.90 (m, 4H), 3.79 (s, 3H), 2.74 – 2.48 (m, 6H), 1.65 (s, 6H), 1.06 (t, J = 7.1 Hz, 6H) ppm; ^{13}C NMR ($CDCl_3$, 101 MHz, 25 °C): δ = 157.4 (s, 1C), 156.8 (s, 1C), 143.4 (s, 1C), 143.2 (s, 1C), 127.8 (d, 2C), 127.8 (d, 2C), 114.0 (d, 2C), 113.3 (d, 2C), 70.6 (t, 1C), 66.0 (d, 1C), 56.1 (t, 1C), 55.27 (q, 1C), 47.3 (t, 2C), 41.8 (s, 1C), 31.2 (q, 2C), 12.0 (q, 2C) ppm; HRMS (ESI+): calculated $[M+H]^+$ = $[C_{23}H_{33}NO_3+H]^+$ 372.2533; found 372.2560.

Model 4 (1-((1,3-bis(4-(2-(4-methoxyphenyl)propan-2-yl)phenoxy)propan-2-yl)oxy)butan-2-ol)

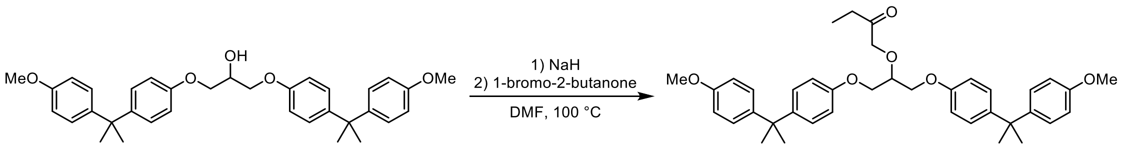

In an Argon-charged glovebox 541 mg (1.00 mmol, 1 equiv) of model 1 were dissolved in 4 ml DMF in a round bottom flask. 32.0 mg (1.2 mmol, 1.2 equiv, 90% purity) of sodium hydride were added and the reaction mixture stirred at room temperature for 30 min. 200 µl (296 mg, 2.0 mmol, 2 equiv) of 1-bromo-2-butanone were added, and the reaction mixture was stirred at 100 °C over-night. After cooling to room temperature and opening under air, 30 ml of water and 15 ml of ethyl acetate were added subsequently, and the organic phase was extracted with three times 15 ml of ethyl acetate, washed with brine, dried over $MgSO_4$ and the solvent was removed *in vacuo*. Column chromatography over silica gel using a gradient from pentane to 4/1 pentane/ethyl acetate afforded the product 1-((1,3-bis(4-(2-(4-methoxyphenyl)propan-2-yl)phenoxy)propan-2-yl)oxy)butan-2-one as a colourless oil in a yield of 32% (198 mg, 325 µmol).

R_f (pentane/ethyl acetate 4/1, silica gel) = 0.53; ^{1}H NMR ($CDCl_3$, 400 MHz, 25 °C): δ = 7.19 – 7.12 (m, 8H), 6.86 – 6.79 (m, 8H), 4.40 (s, 2H), 4.21 – 4.20 (m, 4H), 4.13 – 4.09 (m, 1H), 3.80 (s, 6H), 2.52 (q, *J* = 7.3 Hz, 2H), 1.65 (s, 12H), 1.07 (t, *J* = 7.3 Hz, 3H) ppm; ^{13}C NMR ($CDCl_3$, 101 MHz, 25 °C): δ = 209.7 (s, 1C), 157.5 (s, 2C), 156.3 (s, 2C), 143.8 (s, 2C), 143.1 (s, 2C), 127.9 (d, 4C), 127.8 (d, 4C), 114.0 (d, 4C), 113.4 (d, 4C), 77.7 (d, 1C), 76.0 (t, 1C), 68.1 (t, 2C), 55.3 (q, 2C), 41.8 (s, 2C), 32.2 (t, 1C), 31.2 (q, 4C), 7.31 (q, 1C) ppm; HRMS (ESI+): calculated $[M+Na]^+$ = $[C_{39}H_{46}O_6+Na]^+$ 633.3187; found 633.3202.

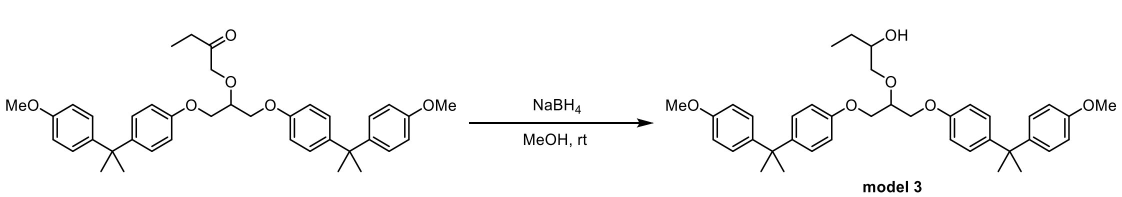

In a round bottom flask, 198 mg (0.325 mmol, 1 equiv) of 1-((1,3-bis(4-(2-(4-methoxyphenyl)propan-2-yl)phenoxy)propan-2-yl)oxy)butan-2-one was dissolved in 3 ml of MeOH under air. 123 mg (3.24 mmol, 10 equiv) of $NaBH_4$ were added and the reaction mixture stirred at room temperature over-night. The resulting solution was taken up in 15 ml of ethyl acetate and washed three times with 10 ml of water. The organic phase was dried over $MgSO_4$ and the solvent was removed *in vacuo*. Column chromatography over silica gel using a gradient from pentane to 3/1 pentane/ethyl acetate afforded the product as a colourless oil in a yield of 80% (159 mg, 260 µmol).

R_f (pentane/ethyl acetate 3/1, silica gel) = 0.3; ^{1}H NMR ($CDCl_3$, 400 MHz, 25 °C): δ = 7.18 – 7.11 (m, 8H), 6.83 – 6.79 (m, 8H), 4.17 – 4.06 (m, 5H), 3.81 – 3.78 (m, 7H), 3.77 – 3.70 (m, 1H), 3.50 (dd, *J* = 10.1, 8.0 Hz, 1H), 2.88 (d, *J* = 3.3 Hz, 1H), 1.64 (s, 12H), 1.59 (s, 1H), 1.54 – 1.42 (m, 2H), 0.97 (t, *J* = 7.5 Hz, 3H) ppm; ^{13}C NMR ($CDCl_3$, 101 MHz, 25 °C): δ = 157.5 (s, 2C), 156.4 (s, 2C), 143.9 (s, 2C), 143.2 (s, 2C), 127.9 (d, 4C), 127.8 (d, 4C), 114.0 (d, 4C), 113.4 (d, 4C), 77.9 (d, 1C), 75.4 (t, 2C), 72.0 (d, 1C), 67.8 (t, 1C), 55.3 (q, 2C), 41.8 (s, 2C), 31.2 (q, 4C),n25.9 (t, 1C), 10.1 (q, 1C) ppm; HRMS (ESI+): calculated $[M+Na]^+$ = $[C_{39}H_{48}O_6+Na]^+$ 635.3343; found 635.3337.

Model 5 (1,3-bis(4-(2-(4-methoxyphenyl)propan-2-yl)phenoxy)propan-2-ol)

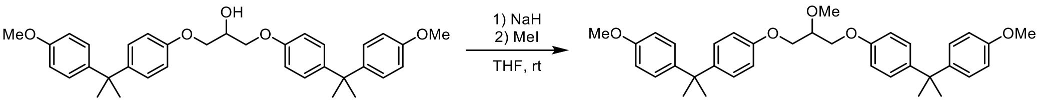

model 4

1.35 g (2.5 mmol, 1.0 equiv) of model 1 were dissolved in 10 ml of THF in a 50 ml Schlenk flask under argon and cooled using an ice/water bath. 200 mg (5 mmol, 2 equiv, 60% dispersion in mineral oil) of sodium hydride were added and the mixture was stirred for 1 h, while being allowed to warm to room temperature. Then, 467 µl (1.07 g, 7.5 mmol, 3 equiv) of iodomethane were added the reaction mixture was stirred over-night at room temperature. 50 ml of water were added carefully to the reaction mixture and the crude product was then extracted using three times 30 ml of DCM. The organic phase was dried over $MgSO_4$ and the solvent removed *in vacuo*. Column chromatography over silica gel using an eluent of 10/1 pentane/ethyl acetate afforded model 2b as colourless solid in a yield of 83% (1.16 g, 2.09 mmol).

R_f (pentane/ethyl acetate 10/1, silica gel) = 0.17; ^{1}H NMR ($CDCl_3$, 400 MHz, 25 °C): δ = 7.14 – 7.12 (m, 8H), 6.83 – 6.7.9 (m, 8H), 4.19 – 4.08 (m, 4H), 3.91 (q, *J* = 5.0 Hz, 1H), 3.78 (s, 6H), 3.56 (s, 3H), 1.63 (s, 12H) ppm, ^{13}C NMR ($CDCl_3$, 101 MHz, 25 °C): δ = 157.5 (s, 2C), 156.6 (s, 2C), 143.7 (s, 2C), 143.2 (s, 2C), 127.9 (d, 4C), 127.9 (d, 4C), 114.0 (d, 4C), 113.4 (d, 4C), 78.2 (q, 1C), 67.2 (t, 2C), 58.5 (d, 1C), 55.3 (q, 2C), 41.8 (s, 2C), 31.2 (d, 4C) ppm; HRMS (ESI+): calculated $[M+Na]^+$ = $[C_{36}H_{42}O_2+Na]^+$ 577.2924; found 577.2910.

Ketone III (1-(4-(2-(4-methoxyphenyl)propan-2-yl)phenoxy)propan-2-one)

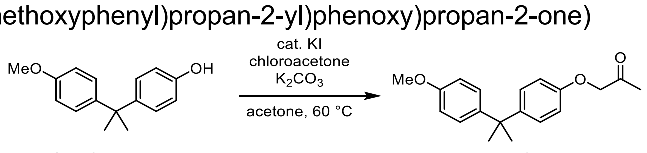

1.21 g (5.0 mmol, 1.0 equiv) of Me-BPA were dissolved in 30 ml of acetone in a round bottom flask. 1.38 g (10.0 mmol, 2.0 equiv) of potassium carbonate, 83.0 mg (0.50 mmol, 0.1 equiv) of potassium iodide and lastly 694 mg (7.50 mmol, 1.5 equiv) of chloroacetone were added and the reaction mixture stirred for 3 h at 60 °C. After the reaction was complete, as confirmed by TLC, it was allowed to cool to rt, and the suspension filtered over a glass frit with acetone. The solvent was removed *in vacuo*. Automated column chromatography using a gradient from heptane 95% / ethyl acetate 5% to heptane 80% / ethyl acetate 20% over silica afforded the product as a colourless oil in a yield of 68 % (1.02 g, 3.42 mmol).

R_f (pentane/ethyl acetate 15/1, silica gel) = 0.2; ^{1}H NMR ($CDCl_3$, 400 MHz, 25 °C): δ = 7.19 – 7.10 (m, 4H), 6.83 – 6.74 (m, 4H), 4.51 (s, 2H), 3.78 (s, 3H), 2.28 (s, 3H), 1.64 (s, 6H) ppm; ^{13}C NMR ($CDCl_3$, 101 MHz, 25 °C): δ = 206.3 (s, 1C), 157.6 (s, 1C), 155.7 (s, 1C), 144.5 (s, 1C), 143.0 (s, 1C), 128.1 (d,

2C), 127.8 (d, 2C), 114.0 (d, 2C), 113.4 (d, 2C), 73.3 (t, 1C), 55.3 (q, 1C), 41.9 (s, 1C), 31.2 (q, 1C), 26.8 (q, 1C) ppm; HRMS (ESI+): calculated $[M+Na]^+$ = $[C_{19}H_{22}O_3+Na]^+$ 321.1461; found 321.1467.

Me-BPS (4-((4-methoxyphenyl)sulfonyl)phenol)

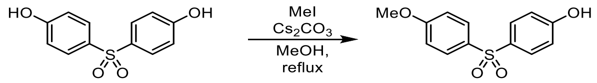

2.00 g (8.00 mmol, 1.0 equiv) of bisphenol S were suspended in 20 ml of methanol in a 10 ml round bottom flask under air. Under stirring, 3.90 g (12.0 mmol, 1.5 equiv) of caesium carbonate were added. Then, 0.50 ml (1.13 g, 8.00 mmol, 1 equiv) of methyl iodide were added. The reaction mixture was stirred over-night at reflux. Afterwards, the suspension was filtered over a glass frit. The solvent was removed *in vacuo*. Automated column chromatography over silica gel using a gradient of heptane 62% / ethyl acetate 33% / methanol 5% to heptane 25% / ethyl acetate 50% / methanol 25% afforded Me-BPS as colourless highly viscous oil in a yield of 37% (772 mg, 2.92 mmol).

^{1}H NMR (MeOD, 400 MHz, 25 °C): δ = 7.79 (d, *J* = 9.0 Hz, 2H), 7.63 (d, *J* = 8.9 Hz, 2H), 7.02 (d, *J* = 9.0 Hz, 2H), 6.75 (d, *J* = 8.8 Hz, 2H), 4.89 (s, 1H), 3.83 (s, 3H) ppm; ^{13}C NMR (MeOD, 101 MHz, 25 °C): δ = 168.7 (s, 2C), 164.5 (s, 2C), 136.0 (s, 2C), 130.7 (d, 2C), 130.1 (d, 2C), 129.3 (s, 2C), 118.5 (d, 2C), 115.5 (d, 2C), 56.2 (q, 1C) ppm; HRMS (ESI+): calculated $[M+Na]^+$ = $[C_{13}H_{12}O_4S+Na]^+$ 287.0349; found 287.0349.

Model 6 (1,3-bis(4-((4-methoxyphenyl)sulfonyl)phenoxy)propan-2-ol)

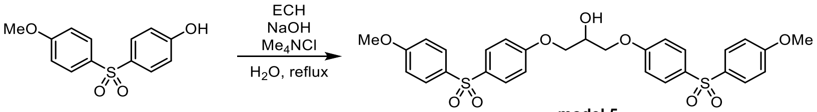

model 5

766 mg (2.90 mmol, 2.0 equiv) of Me-BPS were suspended in 4.5 ml of water in a 10 ml COtube under air. 116 mg (2.90 mmol, 2 equiv) of sodium hydroxide and 15.9 mg (14.5 µmol, 10 mol%) of tetramethylammonium chloride were added and the mixture was stirred at room temperature for 10 min. Then, 114 µl (134 mg, 2.95 mmol, 1 equiv) of epichlorohydrin (ECH) were added. The flask was sealed, and the mixture was heated to reflux over-night under vigorous stirring. The reaction mixture was allowed to cool to room temperature and 15 ml of ethyl acetate were added. The crude product was extracted using three times 20 ml of ethyl acetate. The combined organic phases were dried over $MgSO_4$, filtered, and the solvent removed *in vacuo*. Automated column chromatography over silica gel using a gradient of heptane 75% / ethyl acetate 25% to heptane 56% / ethyl acetate 33% / methanol 10%, afforded model 5 as colourless solid in a yield of 46% (389 mg, 0.67 mmol) still containing minor impurities, which could not be removed be repeated columns or crystallization from methanol.

R_f (pentane/ethyl acetate 2/1, silica gel) = 0.15; ^{1}H NMR ($CDCl_3$, 400 MHz, 25 °C): δ = 7.83 (dd, *J* = 9.0, 2.9 Hz, 8H), 6.95 (dd, *J* = 9.0, 7.4 Hz, 8H), 4.39 (q, *J* = 5.1 Hz, 1H), 4.16 (dd, *J* = 5.3, 3.7 Hz, 4H), 3.83 (s, 6H), 2.57 (d, *J* = 5.4 Hz, 1H) ppm; ^{13}C NMR ($CDCl_3$, 101 MHz, 25 °C): δ = 163.3 (s, 4C), 161.8 (s, 4C), 134.9 (s, 4C), 133.8 (s, 4C), 129.8 (d, 4C), 129.7 (d, 4C), 129.7 (d, 4C), 115.1 (d, 4C), 68.9 (t, 2C), 68.4 (d, 1C), 63.48, 55.8 (1, 2C) ppm; HRMS (ESI+): calculated $[M+Na]^+$ = $[C_{29}H_{28}O_9S_2+Na]^+$ 607.1067; found 607.1069.

4,4'-methylenebis(5-isopropyl-2-methylphenol)

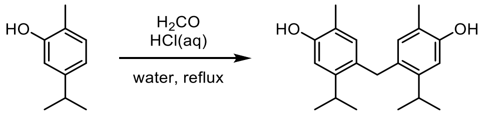

9.01 g (60.0 mmol, 2.0 equiv) of carvacrol and 901 mg (30.0 mmol, 1.0 equiv) of paraformaldehyde were suspended in 60 ml of water and 15 ml of concentrated hydrochloric acid were added carefully.

The reaction mixture was refluxed over-night and then allowed to cool to rt. The reaction mixture was concentrated *in vacuo* and heptane was added. The resulting mixture was sonicated, and then filtrated, affording the title compound as a white solid in a yield of 71% (6.64 g, 21.2 mmol).

^{1}H NMR ($CDCl_3$, 400 MHz, 25 °C): δ = 6.75 (s, 2H), 6.62 (s, 2H), 4.54 (s, 2H), 3.86 (s, 2H), 3.04 (hept, *J* = 6.8 Hz, 2H), 2.14 (s, 6H), 1.18 (d, *J* = 6.8 Hz, 12H) ppm; ^{13}C NMR ($CDCl_3$, 101 MHz, 25 °C): δ = 152.4 (s, 2C), 146.1 (s, 2C), 132.3 (d, 2C), 130.0 (s, 2C), 120.6 (s, 2C), 112.0 (d, 2C), 33.6 (t, 1C), 28.8 (d, 2C), 23.8 (q, 4C), 15.4 (q, 2C) ppm. The NMR spectra are in agreement with reported data[17].

5-isopropyl-4-(2-isopropyl-4-methoxy-5-methylbenzyl)-2-methylphenol

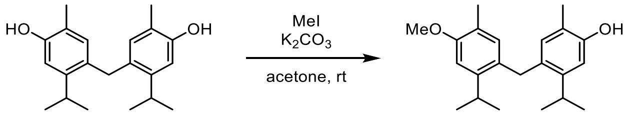

6.31 g (20.2 mmol,1.0 equiv) of 4,4'-methylenebis(5-isopropyl-2-methylphenol) and 4.19 mg (30.3 mg, 1.5 equiv) of potassium carbonate were suspended in 40 ml of acetone under air and 1.26 ml (2.87 g, 20.2 mmol, 1.0 equiv) of methyl iodide were added. The reaction mixture was stirred over-night at rt. The reaction mixture was then filtered through a plug of Celite using acetone and the solvent removed *in vacuo*. Automated column chromatography over silica gel using a gradient of heptane 100% to heptane 80% / ethyl acetate 20% afforded the title compound as yellow oil in a yield of 40% (2.63 g, 8.06 mmol).

^{1}H NMR ($CDCl_3$, 400 MHz, 25 °C): δ = 6.78 (s, 1H), 6.74 (s, 1H), 6.64 (s, 1H), 6.61 (s, 1H), 4.49 (s, 1H), 3.87 (s, 2H), 3.85 (s, 3H), 3.16 – 2.99 (m, 2H), 2.13 (s, 3H), 2.10 (s, 3H), 1.21 (d, *J* = 6.8 Hz, 6H), 1.18 (d, *J* = 6.8 Hz, 6H) ppm; ^{13}C NMR ($CDCl_3$, 101 MHz, 25 °C): δ = 156.5 (s, 1C), 152.4 (s, 1C), 146.1 (s, 1C), 145.4 (s, 1C), 132.3 (d, 1C), 132.1 (d, 1C), 130.0 (s, 1C), 129.3 (s, 1C), 123.7 (s, 1C), 120.6 (s, 1C), 112.0 (d, 1C), 107.1 (d, 1C), 55.5 (q, 1C), 33.6 (t, 1C), 29.1 (d, 1C), 28.8 (d, 1C), 23.9 (q, 2C), 23.8 (q, 2C), 15.8 (q, 1C), 15.4 (q, 1C) ppm; HRMS (ESI+): calculated $[M+Na]^+$ = $[C_{22}H_{30}O_2+Na]^+$ 349.2138; found 349.2137.

Model 7 (1,3-bis(5-isopropyl-4-(2-isopropyl-4-methoxy-5-methylbenzyl)-2-methylphenoxy)propan-2-ol)

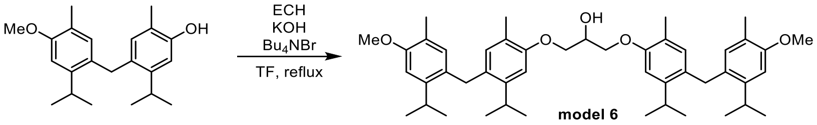

2.60 g (7.65 mmol, 2.0 equiv) of 5-isopropyl-4-(2-isopropyl-4-methoxy-5-methylbenzyl)-2-methylphenol were dissolved in 80 ml of THF under air. 515 mg (9.18 mmol, 2.4 equiv) of potassium hydroxide and 493 mg (1.53 mmol, 20 mol%) of tetrabutylammonium bromide were added and the mixture was stirred at room temperature for 10 min. Then, 350 µl (412 mg, 3.8 mmol, 1 equiv) of epichlorohydrin (ECH) were added. The flask was sealed, and the mixture was heated to reflux over-night under vigorous stirring. The reaction mixture was allowed to cool to room temperature and taken up in 100 ml of water. The crude product was extracted using three times 50 ml of ethyl acetate. The combined organic phases were dried over $MgSO_4$, filtered, and the solvent removed *in vacuo*. Automated column chromatography over silica gel using a gradient of heptane 100% to heptane 87.5% / ethyl acetate 12.5% afforded model 6 as colourless solid in a yield of 44% (1.19 g, 1.68 mmol).

^{1}H NMR ($CDCl_3$, 400 MHz, 25 °C): δ = 6.81 (s, 2H), 6.78 (s, 2H), 6.65 (s, 2H), 6.63 (s, 2H), 4.45 (p, *J* = 5.3 Hz, 1H), 4.25 – 4.18 (m, 4H), 3.88 (s, 4H), 3.85 (s, 6H), 3.10 (p, *J* = 6.8 Hz, 4H), 2.61 (d, *J* = 5.1 Hz, 1H), 2.13 (s, 6H), 2.10 (s, 6H), 1.22 – 1-19 (m, 24H) ppm; ^{13}C NMR ($CDCl_3$, 101 MHz, 25 °C): δ = 156.5 (s, 2C), 155.3 (s, 2C), 145.6 (s, 2C), 145.3 (s, 2C), 132.2 (d, 2C), 132.1 (d, 2C), 130.1 (s, 2C), 129.2 (s, 2C), 123.9 (s, 2C), 123.7 (s, 2C), 108.4 (d, 2C), 107.1 (d, 2C), 69.3 (d, 1C), 69.1 (t, 2C), 55.5 (q, 2C), 33.6 (t, 2C), 29.1 (d, 2C), 29.1 (d, 2C), 23.9 (q 4C), 23.9 (q, 4C), 15.9 (q, 2C), 15.9 (q, 2C) ppm; HRMS (ESI+): calculated $[M+NH_4]^+$ = $[C_{47}H_{64}O_5+NH_4]^+$ 726.5092; found 726.5101.

4-(3,4-dimethoxybenzyl)-2-methoxyphenol

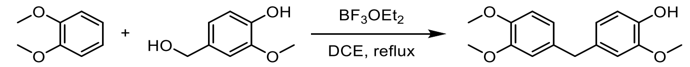

771 mg (5.0 mmol, 1.0 equiv) of vanillyl alcohol and 3.45 g (25.0 mmol, 5.0 equiv) of veratrole were dissolved in 7 ml of DCE in 40 ml COtube under air. The reaction vessel was purged with argon and 740 µl (852 mg, 6.00 mmol, 1.2 equiv) BF_3*OEt_2 was added. The reaction mixture was refluxed over-night and then allowed to cool to rt. The solvent was removed *in vacuo* and automated column chromatography over silica gel using a gradient of heptane 90% / ethyl acetate 10% to heptane 55% / ethyl acetate 40%/ methanol 5% afforded the title compound as colourless oil in a yield of 91% (1.25 g, 4.56 mmol).

^{1}H NMR ($CDCl_3$, 400 MHz, 25 °C): δ = 6.86 – 6.84 (m, 1H), 6.81 – 6.79 (m, 1H), 6.75 – 6.65 (m, 4H), 5.50 (s, 1H), 3.86 (m, 5H), 3.83 (s, 6H) ppm; ^{13}C NMR ($CDCl_3$, 101 MHz, 25 °C): δ = 149.0 (s, 1C), 147.5 (s, 1C), 146.6 (s, 1C), 144.0 (s, 1C), 134.1 (s, 1C), 133.3 (s, 1C), 121.6 (d, 1C), 120.9 (d, 1C), 120.8 (d, 1C), 114.3 (d, 1C), 112.2 (d, 1C), 111.4 (d, 1C), 111.3 (d, 1C), 56.0 (q, 1C), 56.0 (q, 1C), 55.9 (q, 1C), 41.2 (t, 1C) ppm; HRMS (ESI+): calculated $[M+Na]^+$ = $[C_{16}H_{18}O_4+Na]^+$ 297.1097; found 297.1104.

Model 8 1,3-bis(4-(3,4-dimethoxybenzyl)-2-methoxyphenoxy)propan-2-ol

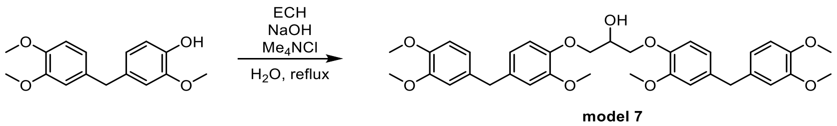

466 mg (1.70mmol, 2.0 equiv) of 4-(3,4-dimethoxybenzyl)-2-methoxyphenol were suspended in 5 ml of water in a 10 ml COtube under air. 68.0 mg (1.70 mmol, 2 equiv) of sodium hydroxide and 9.32 mg (8.50 µmol, 10 mol%) of tetramethylammonium chloride were added and the mixture was stirred at room temperature for 10 min. Then, 78.8 µl (78.6 mg, 850 µmol, 1 equiv) of epichlorohydrin (ECH) were added. The tube was sealed, and the mixture was heated to reflux over-night under vigorous stirring. The reaction mixture was allowed to cool to room temperature and 15 ml of ethyl acetate were added. The crude product was extracted using three times 20 ml of ethyl acetate. The combined organic phases were dried over $MgSO_4$, filtered, and the solvent removed *in vacuo*. Automated column chromatography over silica gel using a gradient of heptane 80% / ethyl acetate 20% to heptane 50% / 50% ethyl acetate, afforded model 7 as colourless highly viscous oil in a yield of 51% (263 mg, 0.44 mmol).

^{1}H NMR ($CDCl_3$, 400 MHz, 25 °C): δ = 6.89 – 6.87 (m, 2H), 6.81 – 6.79 (m, 2H), 6.74 – 6.67 (m, 8H), 4.37 (t, J = 5.5 Hz, 1H), 4.21 – 4.09 (m, 4H), 3.87 (s, 4H), 3.86 (s, 6H), 3.83 (s, 6H), 3.79 (s, 6H) ppm; ^{13}C NMR ($CDCl_3$, 101 MHz, 25 °C): δ = 150.0 (s, 2C), 149.0 (s, 2C), 147.5 (s, 2C), 146.6 (s, 2C), 135.5 (s, 2C), 133.8 (s, 2C), 121.1 (d, 2C), 120.9 (d, 2C), 115.4 (d, 2C), 112.8 (d, 2C), 112.2 (d, 2C), 111.3 (d, 2C), 71.4 (t, 2C), 68.8 (d, 1C), 56.0 (q, 2C), 56.0 (q, 2C), 56.0 (q, 2C), 41.2 (t, 2C) ppm; HRMS (ESI+): calculated $[M+NH_4]^+$ = $[C_{35}H_{40}O_9+NH_4]^+$ 622.3011; found 622.3011.

(5-(methoxymethyl)furan-2-yl)methanol

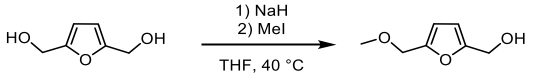

In an Argon-charged glovebox, 2.56 g (20.0 mmol, 1.0 equiv) of 2,5-bis(hydroxymethyl)furan were dissolved in 10 ml of THF in a 40 ml COtube. 553 mg (20.0 mmol, 1 equiv, 90% purity) of NaH were suspended in 10 ml of THF and slowly added to the COtube under stirring at rt. The COtube was sealed and taken out of the glovebox. Under stirring at rt, 1.25 ml (2.85 g, 20.0 mmol, 1 equiv) of methyl iodide were added through the septum and the reaction mixture was stirred over-night at 40 °C. The reaction was carefully quenched with water and then the mixture was neutralised using 4 M HCl. The organic products were extracted using three times 25 ml of CH_2Cl_2. The combined organic phases were dried over Na_2SO_4, filtered, and the solvent removed *in vacuo*. Automated column chromatography over silica

gel using a gradient of heptane 100% to ethyl acetate 100%, afforded the title compound as colourless oil in a yield of 26% (751 mg, 5.28 mmol).

^{1}H NMR ($CDCl_3$, 400 MHz, 25 °C): δ = 6.28 – 6.27 (m, 1H), 6.24 (m, 1H), 4.59 (d, J = 5.6 Hz, 2H), 4.37 (s, 2H), 3.37 (s, 3H), 1.94 (t, J = 6.0 Hz, 1H) ppm; ^{13}C NMR ($CDCl_3$, 101 MHz, 25 °C): δ = 154.5 (s, 1C), 151.7 (s, 1C), 110.3 (d, 1C), 108.5 (d, 1C), 66.6 (t, 1C), 58.1 (q, 1C), 57.7 (t, 1C) ppm. The NMR spectra are in agreement with reported data[18].

Model 9 1,3-bis((5-(methoxymethyl)furan-2-yl)methoxy)propan-2-ol

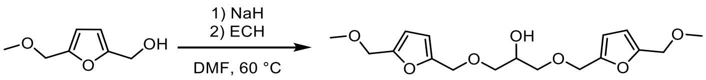

In an Argon-charged glovebox, 462 mg (3.25 mmol, 2.0 equiv) of (5-(methoxymethyl)furan-2-yl)methanol were dissolved in 2 ml of DMF in a 10 ml COtube. 86.7 mg (3.25 mmol, 2 equiv, 90% purity) of NaH were suspended in 1 ml of DMF and added to the COtube under stirring at rt. After 30 min of stirring at rt, 130 µl (153 mg, 1.60 mmol, 1 equiv) of epichlorohydrin (ECH) were added and the reaction mixture was stirred for three days at 60 °C. The reaction was allowed to cool to rt and then carefully quenched with water. The resulting mixture was neutralised with 4 M HCl. The organic products were extracted using three times 50 ml of chloroform from isopropanol. The combined organic phases were dried over Na_2SO_4, filtered, and the solvent removed *in vacuo*. Automated column chromatography over silica gel using a gradient of heptane 100% to ethyl acetate 100%, afforded the title compound as colourless oil in a yield of 30% (165 mg, 484 µmol).

^{1}H NMR ($CDCl_3$, 400 MHz, 25 °C): δ = 6.27 (s, 4H), 4.46 (s, 4H), 4.37 (s, 4H), 3.98 -3-91 (m, 1H), 3.57 – 3.45 (m, 4H), 3.36 (s, 6H), 2.47 (d, J = 4.3 Hz, 1H) ppm; ^{13}C NMR ($CDCl_3$, 101 MHz, 25 °C): δ = 152.1 (s, 2C), 151.9 (s, 2C), 110.3 (d, 2C), 110.1 (d, 2C), 71.2 (t, 2C), 69.6 (d, 1C), 66.6 (t, 2C), 65.5 (t, 2C), 58.1 (q, 2C) ppm; HRMS (ESI+): calculated $[M+NH_4]^+$ = $[C_{17}H_{24}O_7+NH_4]^+$ 358.1860; found 358.1864.

5. Catalytic Deconstruction

5.1 On Model Substrates

General procedure 4.1: 1 equiv of model substrate is given into 10 ml COtube. In an Argon-charged glovebox, 3 mol% of triphos-Ru-TMM, toluene and 3 equiv of isopropanol are added and the reaction vessel sealed. Outside of the glovebox, the reaction mixture is stirred at 650 rpm in an aluminium block at 160 °C for 16 h. The reaction mixture is then cooled to room temperature, the solvent removed *in vacuo* and products isolated using column chromatography.

Deconstruction of Model 1

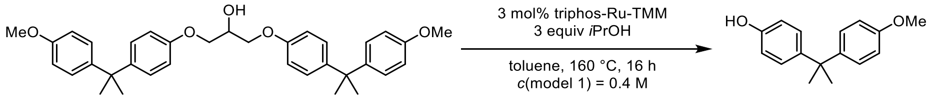

model 1 **Me-BPA**

The reaction was carried out according to the general procedure 4.1. 43.3 mg (0.08 mmol, 1 equiv) of model 1, 1.87 mg (2.40 µmol, 3 mol%) of triphos-Ru-TMM, 18.4 µl (14.4 mg, 0.24 mmol, 3 equiv) of isopropanol and 0.2 mol of toluene were used. Column chromatography over silica gel using 10/1 pentane/ethyl acetate as eluent afforded Me-BPA as colourless oil in a yield of 83% (64.3 mg, 0.27 mmol).

R_f (pentane/ethyl acetate 4/1, silica gel) = 0.3; ^{1}H NMR ($CDCl_3$, 400 MHz, 25 °C): δ = 7.16 – 7.14 (m, 2H), 7.11 – 7.09 (m, 2H), 6.83 – 6.81 (m, 2H), 6.74 – 6.72 (m, 2H), 4.76 (s, 1H), 3.80 (s, 3H), 1.64 (s, 6H) ppm. The NMR spectra are in agreement with reported data[16].

Deconstruction of Model 2

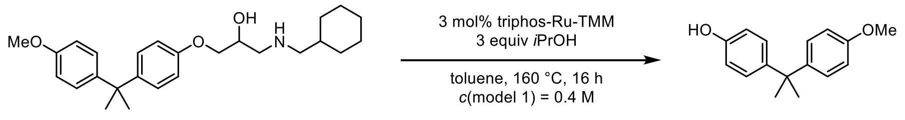

model 2 **Me-BPA**

The synthesis was carried out according to the general procedure 4.1. 65.9 mg (0.16 mmol, 1 equiv) of model 3, 3.74 mg (4.80 µmol, 3 mol%) of triphos-Ru-TMM, 36.7 µl (28.8 mg, 0.48 mmol, 3 equiv) of isopropanol and 0.4 mol of toluene were used. Column chromatography over silica gel using 10/1 pentane/ethyl acetate as eluent afforded Me-BPA as colourless oil in a yield of 88% (34.1 mg, 0.14 mmol).

R_f (pentane/ethyl acetate 4/1, silica gel) = 0.3; ^{1}H NMR ($CDCl_3$, 400 MHz, 25 °C): δ = 7.16 – 7.14 (m, 2H), 7.11 – 7.09 (m, 2H), 6.83 – 6.81 (m, 2H), 6.74 – 6.72 (m, 2H), 4.76 (s, 1H), 3.80 (s, 3H), 1.64 (s, 6H) ppm. The NMR spectra are in agreement with reported data[16].

Deconstruction of Model 3

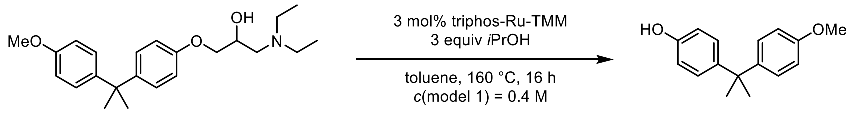

model 3 **Me-BPA**

The synthesis was carried out according to the general procedure 4.1. 59.4 mg (0.16 mmol, 1 equiv) of model 3, 3.74 mg (4.80 µmol, 3 mol%) of triphos-Ru-TMM, 36.7 µl (28.8 mg, 0.48 mmol, 3 equiv) of isopropanol and 0.4 mol of toluene were used. Column chromatography over silica gel using 10/1 pentane/ethyl acetate as eluent afforded Me-BPA as colourless oil in a yield of 80% (30.9 mg, 0.13 mmol).

R_f (pentane/ethyl acetate 4/1, silica gel) = 0.3; ^{1}H NMR ($CDCl_3$, 400 MHz, 25 °C): δ = 7.16 – 7.14 (m, 2H), 7.11 – 7.09 (m, 2H), 6.83 – 6.81 (m, 2H), 6.74 – 6.72 (m, 2H), 4.76 (s, 1H), 3.80 (s, 3H), 1.64 (s, 6H) ppm. The NMR spectra are in agreement with reported data[16].

Deconstruction of Model 6

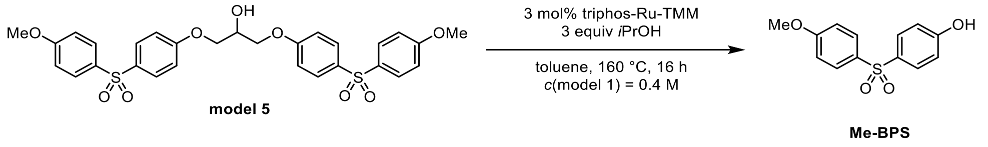

The synthesis was carried out according to the general procedure 4.1. 46.8 mg (0.08 mmol, 1 equiv) of model 5, 1.87 mg (2.40 µmol, 3 mol%) of triphos-Ru-TMM, 18.4 µl (14.4 mg, 0.24 mmol, 3 equiv) of isopropanol and 0.2 mol of toluene were used. Automated column chromatography over silica gel using a gradient of heptane 80%/ ethyl acetate 20% / to 25% heptane / ethyl acetate 50% / methanol 25% eluent afforded Me-BPA as colourless oil in a yield of 74% (31.0 mg, 0.12 mmol).

^{1}H NMR (MeOD, 400 MHz, 25 °C): δ = 7.79 (d, *J* = 9.0 Hz, 2H), 7.63 (d, *J* = 8.9 Hz, 2H), 7.02 (d, *J* = 9.0 Hz, 2H), 6.75 (d, *J* = 8.8 Hz, 2H), 4.89 (s, 1H), 3.83 (s, 3H) ppm. Data is consistent with the one reported here for Me-BPS.

Deconstruction of Model 7

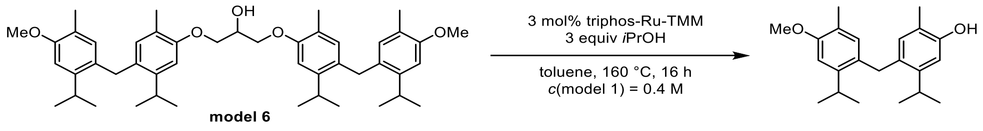

The synthesis was carried out according to the general procedure 4.1. 56.7 mg (0.08 mmol, 1 equiv) of model 6, 1.87 mg (2.40 µmol, 3 mol%) of triphos-Ru-TMM, 18.4 µl (14.4 mg, 0.24 mmol, 3 equiv) of isopropanol and 0.2 mol of toluene were used. Automated column chromatography over silica gel using a gradient of heptane 100% to heptane 80% / ethyl acetate 20% as eluent afforded Me-BPA as colourless oil in a yield of 86% (44.8 mg, 0.14 mmol).

^{1}H NMR ($CDCl_3$, 400 MHz, 25 °C): δ = 6.78 (s, 1H), 6.74 (s, 1H), 6.64 (s, 1H), 6.61 (s, 1H), 4.49 (s, 1H), 3.87 (s, 2H), 3.85 (s, 3H), 3.16 – 2.99 (m, 2H), 2.13 (s, 3H), 2.10 (s, 3H), 1.21 (d, *J* = 6.8 Hz, 6H), 1.18 (d, *J* = 6.8 Hz, 6H) ppm. Data is consistent with the one reported here for 5-isopropyl-4-(2-isopropyl-4-methoxy-5-methylbenzyl)-2-methylphenol.

Deconstruction of Model 8

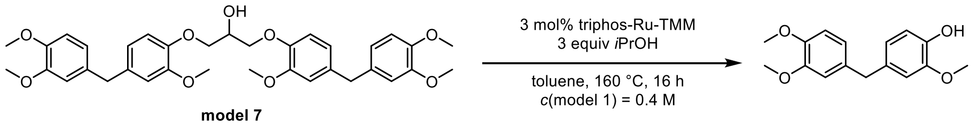

The synthesis was carried out according to the general procedure 4.1. 48.4 mg (0.08 mmol, 1 equiv) of model 6, 1.87 mg (2.40 µmol, 3 mol%) of triphos-Ru-TMM, 18.4 µl (14.4 mg, 0.24 mmol, 3 equiv) of isopropanol and 0.2 mol of toluene were used. Automated column chromatography over silica gel using a gradient of heptane 100% to heptane 80% / ethyl acetate 20% as eluent afforded Me-BPA as colourless oil in a yield of 57% (30.5 mg, 0.09 mmol).

^{1}H NMR ($CDCl_3$, 400 MHz, 25 °C): δ = 6.86 – 6.84 (m, 1H), 6.81 – 6.79 (m, 1H), 6.75 – 6.65 (m, 4H), 5.50 (s, 1H), 3.86 (m, 5H), 3.83 (s, 6H) ppm. Data is consistent with the one reported here for 4-(3,4-dimethoxybenzyl)-2-methoxyphenol.

5.2 On Amine-Cured Epoxy Resins

General procedure 4.2: 100 mg of finely powdered epoxy resin are given into 10 ml COtube. In an Argon-charged glovebox, 6.0 wt% of triphos-Ru-TMM, 1.0 ml of toluene and 80.0 µl (1.05 mmol) of isopropanol are added and the reaction vessel sealed. Outside of the glovebox, the reaction mixture is stirred at 650 rpm in an aluminium block at 160 °C for 24 h. The reaction mixture is then allowed to cool to room temperature and the residues taken up in acetone and transferred into a round bottom flask. Celite is added and the solvent removed *in vacuo*. The resulting mixture is loaded onto a silica gel charged column. Column chromatography using a gradient of 6/1 pentane/ethyl acetate to 4/1 pentane/ethyl acetate affords bisphenol A. Afterwards, the rest fraction is eluted from the column using 10% MeOH in DCM.

The deconstruction of **Airstone 760E/766H** (approx. BPA content 43 wt%) was carried out according to the general procedure 4.2. 100 mg of the powdered resin, 6.0 mg (7.69 µmol, 6 wt%) of triphos-Ru-TMM, 80 µl (62.9 mg, 1.05 mmol) of isopropanol and 1.0 ml of toluene were used. Column chromatography afforded BPA as a colourless solid and a rest fraction as brown highly viscous oil.

BPA: Yield of 56% (24.3 mg, 107 µmol); R_f (pentane/ethyl acetate 4/1, silica gel) = 0.21; ^1H NMR ($CDCl_3$, 400 MHz, 25 °C): δ = 7.11 – 7.07 (m, 4H), 6.76 – 6.70 (m, 4H), 4.57 (s, 2H), 1.62 (s, 6H) ppm. The NMR spectra are in agreement with reported data[19].

Rest fraction: Yield of 60.1 mg (60 wt%).

The deconstruction of **UHU 2-component glue** (approx. BPA content 34 wt%) was carried out according to the general procedure 4.2. 100 mg of the powdered resin, 6.0 mg (7.69 µmol, 6.0 wt%) of triphos-Ru-TMM, 80 µl (62.9 mg, 1.05 mmol) of isopropanol and 1.0 ml of toluene were used. Column chromatography afforded of BPA as colourless solid and a rest fraction as brown highly viscous oil.

BPA: Yield of 38% (12.9 mg, 56.5 µmol); R_f (pentane/ethyl acetate 4/1, silica gel) = 0.21; ^1H NMR ($CDCl_3$, 400 MHz, 25 °C): δ = 7.11 – 7.07 (m, 4H), 6.76 – 6.70 (m, 4H), 4.57 (s, 2H), 1.62 (s, 6H) ppm. The NMR spectra are in agreement with reported data[19].

Rest fraction: Yield of 20.8 mg (21 wt%).

The deconstruction of **Roizefar Epoxy Resin** (approx. BPA content 30 wt%) was carried out according to the general procedure 4.2. 100 mg of the powdered resin, 6.0 mg (7.69 µmol, 6 wt%) of triphos-Ru-TMM, 80 µl (62.9 mg, 1.05 mmol) of isopropanol and 1.0 ml of toluene were used. Column chromatography afforded a fraction containing both BPA and of isomers of cresols as colourless solid. Furthermore, a rest fraction was obtained as brown highly viscous oil.

BPA: Yield of 50% (15.0 mg, 65.7 µmol); R_f (pentane/ethyl acetate 4/1, silica gel) = 0.21; ^1H NMR ($CDCl_3$, 400 MHz, 25 °C): δ = 7.11 – 7.07 (m, 4H), 6.76 – 6.70 (m, 4H), 4.57 (s, 2H), 1.62 (s, 6H) ppm. The NMR spectra are in agreement with reported data[15].

Isomers of cresols (2 isomers 2:1): Yield of 9 wt% (9 mg, 83.2 µmol); ^1H NMR (d_6-acetone, 400 MHz, 25 °C): δ = 7.17 – 7.09 (m, 2H), 6.89 – 6.79 (m, 2H), 2.86 (s, 1H), 2.83 (s, 2H) ppm. The NMR spectra are in agreement with reported data[20].

Rest fraction: Yield of 32.8 mg (33 wt%).

The deconstruction of **Sicomin SR infugreen 810/SD8822** (approx. BPA content 36 wt%) was carried out according to the general procedure 4.2. 100 mg of the powdered resin, 6.0 mg (7.69 µmol, 6 wt%) of triphos-Ru-TMM, 80 µl (62.9 mg, 1.05 mmol) of isopropanol and 1.0 ml of toluene were used. Column chromatography afforded BPA as colourless solid and a rest fraction as brown highly viscous oil.

BPA: Yield of 54% (19.5 mg, 85.4 µmol); R_f (pentane/ethyl acetate 4/1, silica gel) = 0.21; ^1H NMR ($CDCl_3$, 400 MHz, 25 °C): δ = 7.11 – 7.07 (m, 4H), 6.76 – 6.70 (m, 4H), 4.57 (s, 2H), 1.62 (s, 6H) ppm. The NMR spectra are in agreement with reported data[19].

Rest fraction: Yield of 48.3 mg (48 wt%).

5.2 On Fiber Reinforced Epoxy Composites

General procedure 4.3: A piece (between 1 cm to 1.5 cm in length and width) of an epoxy composite is placed in a 40 ml COtube. In an Argon-charged glovebox, 6 wt% of triphos-Ru-TMM is added. Then, 1.0 ml of toluene and 80 µl (1.05 mmol) isopropanol each are added per 100 mg of composite and the reaction vessel is sealed. Outside of the glovebox, the reaction mixture is stirred at 650 rpm in an aluminium block at 160 °C until the composite has visibly disassembled. The reaction mixture is allowed to cool to room temperature and the solution decanted off using a syringe. The fibers are washed using acetone and DCM, then dried *in vacuo*. The remaining reaction mixture is transferred it into a round bottom flask. Celite® is added and the solvent removed *in vacuo*. The resulting powder is loaded onto a silica gel charged column. Column chromatography using gradient of 6/1 pentane/ethylacetate to 4/1 pentane/ethylacetate affords bisphenol A. Afterwards, the rest fraction is eluted from the column using 10% MeOH in DCM.

The deconstruction of a piece of **carbon fiber-based landfilled material (Fig. 3A, a))** was carried out according to the general procedure 4.3. A cube of the material (187 mg), 11.4 mg (14.6 µmol, 6 wt%) of triphos-Ru-TMM, 152 µl (119 mg, 1.99 mmol) of isopropanol and 1.6 ml of toluene were used. The reaction was left to stir for 3 days. Carbon fibers were recovered. Column chromatography afforded BPA as colourless solid and a rest fraction as brown highly viscous oil.

Carbon fibers: Yield of 57 wt% (106 mg).

BPA: Yield of 13 wt% (23.4 mg, 102 µmol); R_f (pentane/ethyl acetate 4/1, silica gel) = 0.21; ^{1}H NMR ($CDCl_3$, 400 MHz, 25 °C): δ = 7.11 – 7.07 (m, 4H), 6.76 – 6.70 (m, 4H), 4.57 (s, 2H), 1.62 (s, 6H) ppm. The NMR spectra are in agreement with reported data[19].

Rest fraction: Yield of 26 wt% (49.2 mg).

The deconstruction of a piece of **commercial product sample of a glass fiber-based laminate (Fig. 3A, b))** was carried out according to the general procedure 4.3. A cube of the material (390 mg), 23.4 mg (30.0 µmol, 6 wt%) of triphos-Ru-TMM, 312 µl (245 mg, 4.08 mmol) of isopropanol and 3.9 ml of toluene were used. The reaction was left to stir for 3 days. Glass fibers were recovered. Column chromatography afforded BPA as colourless solid and a rest fraction as brown highly viscous oil.

Glass fibers: Yield of 53 wt% (207 mg).

BPA: Yield of 15 wt% (60.1 mg, 264 µmol); R_f (pentane/ethyl acetate 4/1, silica gel) = 0.21; ^{1}H NMR ($CDCl_3$, 400 MHz, 25 °C): δ = 7.11 – 7.07 (m, 4H), 6.76 – 6.70 (m, 4H), 4.57 (s, 2H), 1.62 (s, 6H) ppm. The NMR spectra are in agreement with reported data[19].

Rest fraction: Yield of 13 wt% (52.3 mg).

The deconstruction of a glass fiber-based piece of **the outer shell of a wind turbine blade (Fig. 3A, c))** was carried out according to the general procedure 4.3. A cube of the material (216 mg), 12.6 mg (16.2 µmol, 6.0 wt%) of triphos-Ru-TMM, 168 µl (132 mg, 2.2 mmol) of isopropanol and 2.1 ml of toluene were used. The reaction was left to stir for 3 days. Glass fibers and a piece of metal grid were recovered. Column chromatography afforded of BPA as colourless solid and a rest fraction as brown highly viscous oil.

Glass fibers: Yield of 50 wt% (108 mg).

BPA: Yield of 19 wt% (40.0 mg, 175 µmol); R_f (pentane/ethyl acetate 4/1, silica gel) = 0.21; ^{1}H NMR ($CDCl_3$, 400 MHz, 25 °C): δ = 7.11 – 7.07 (m, 4H), 6.76 – 6.70 (m, 4H), 4.57 (s, 2H), 1.62 (s, 6H) ppm. The NMR spectra are in agreement with reported data[19].

Rest fraction: Yield of 23 wt% (49.2 mg).

Metal grid: Yield of 5 wt% (10 mg).

Upscaling Experiment

The deconstruction of a glass fiber-based piece of **the outer shell of a wind turbine blade (Fig. 3B)** was carried out according to the general procedure 4.3. The reaction was set up in a 300 ml steel autoclave with a Teflon inlay. A rectangle shaped piece of the material (5.13 g), 300 mg (385 µmol, 6.0 wt%) of triphos-Ru-TMM, 4.0 ml (3.14 g, 52.3 mmol) of isopropanol and 50 ml of toluene were used. The reaction was left to stir for 6 days. Glass fibers and a piece of metal grid were recovered. Column chromatography afforded of BPA as an off white solid and a rest fraction as brown highly viscous oil.

Glass fibers: Yield of 47 wt% (2.39 g).

BPA: Yield of 18 wt% (918 mg, 4.02 mmol); R_f (pentane/ethyl acetate 4/1, silica gel) = 0.21; ^{1}H NMR ($CDCl_3$, 400 MHz, 25 °C): δ = 7.11 – 7.07 (m, 4H), 6.76 – 6.70 (m, 4H), 4.57 (s, 2H), 1.62 (s, 6H) ppm. The NMR spectra are in agreement with reported data[19].

Rest fraction: Yield of 27 wt% (1.40vg).

Metal grid: Yield of 5 wt% (277 mg).

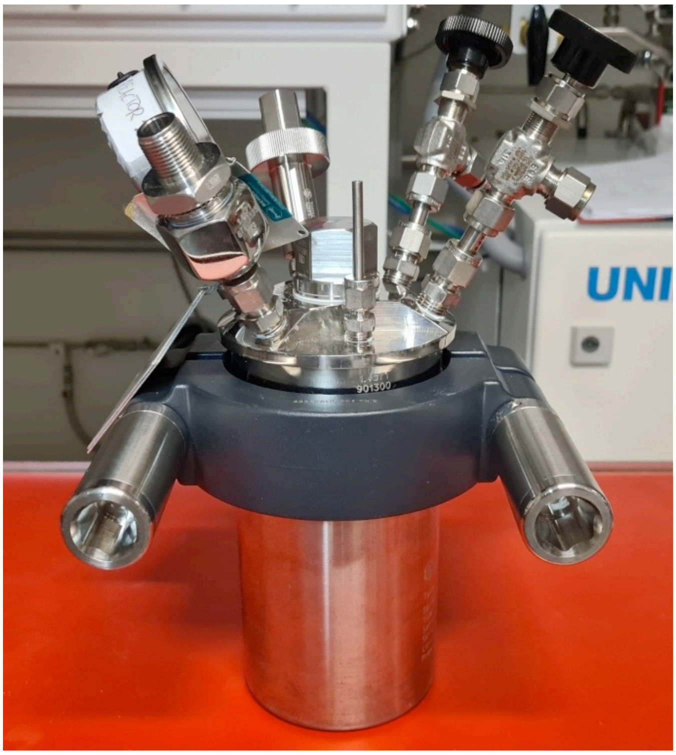

5.3 Control Experiment on Wind Turbine blade Composite

A piece of wind turbine blade composite (286 mg) was placed in a 40 ml COtube. In an Argon-charged glovebox, 3.0 ml of toluene and 240 µl of isopropanol were added and the reaction vessel is sealed. Outside of the glovebox, the reaction mixture is stirred at 650 rpm in an aluminium block at 160 °C for 3 days. The resulting reaction mixture was analysed using ^{1}H NMR spectroscopy and GC-MS, however no compounds could be detected. Also, no fibers or metal grid piece were liberated.

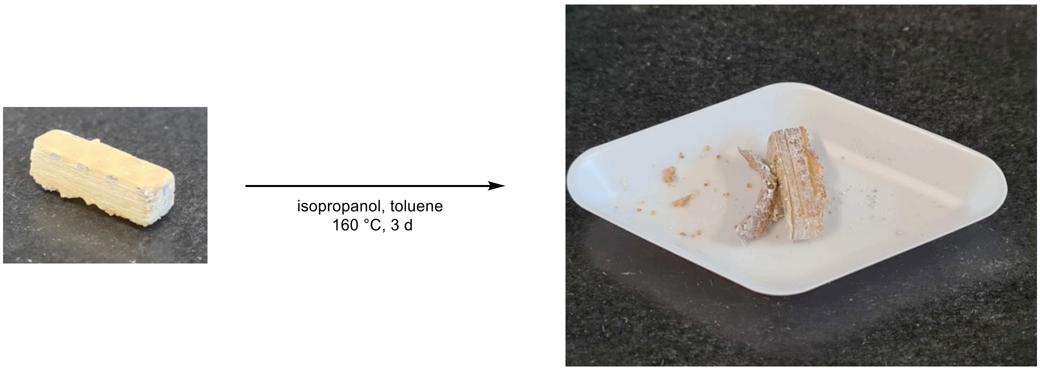

6. Analytical Data

6.1 NMR Spectra of Synthesised Compounds

triphos-Ru-HCl-CO

a) ^{1}H NMR spectrum

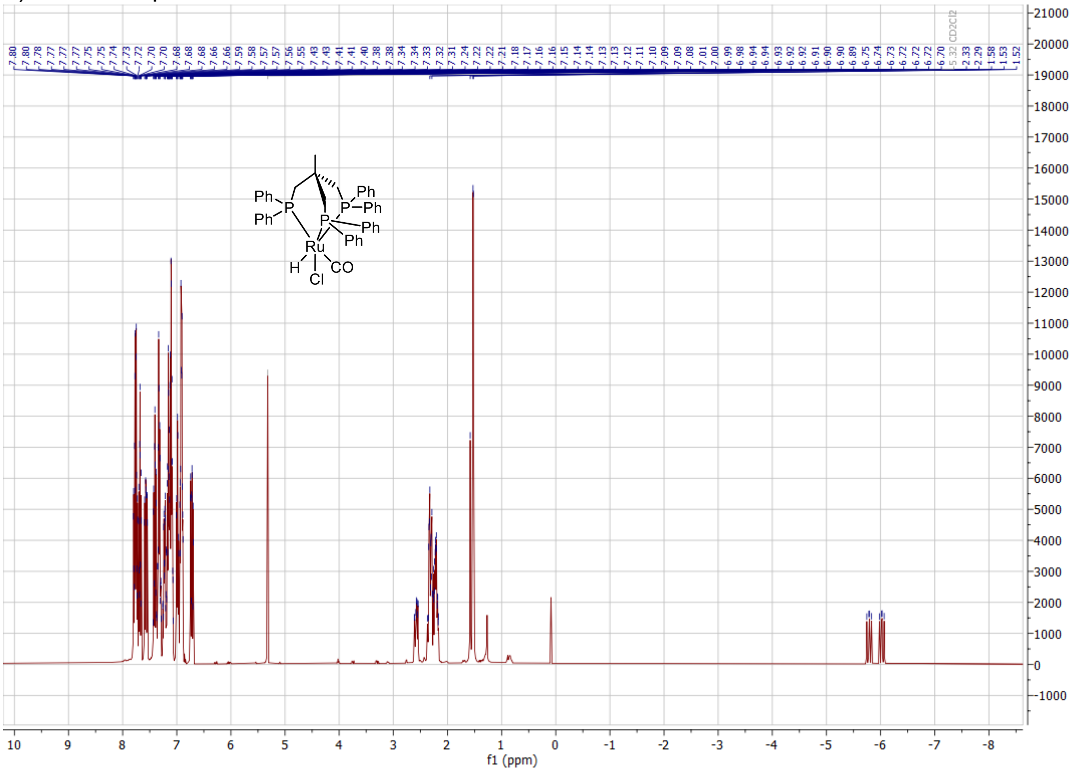

b) ^{31}P NMR spectrum

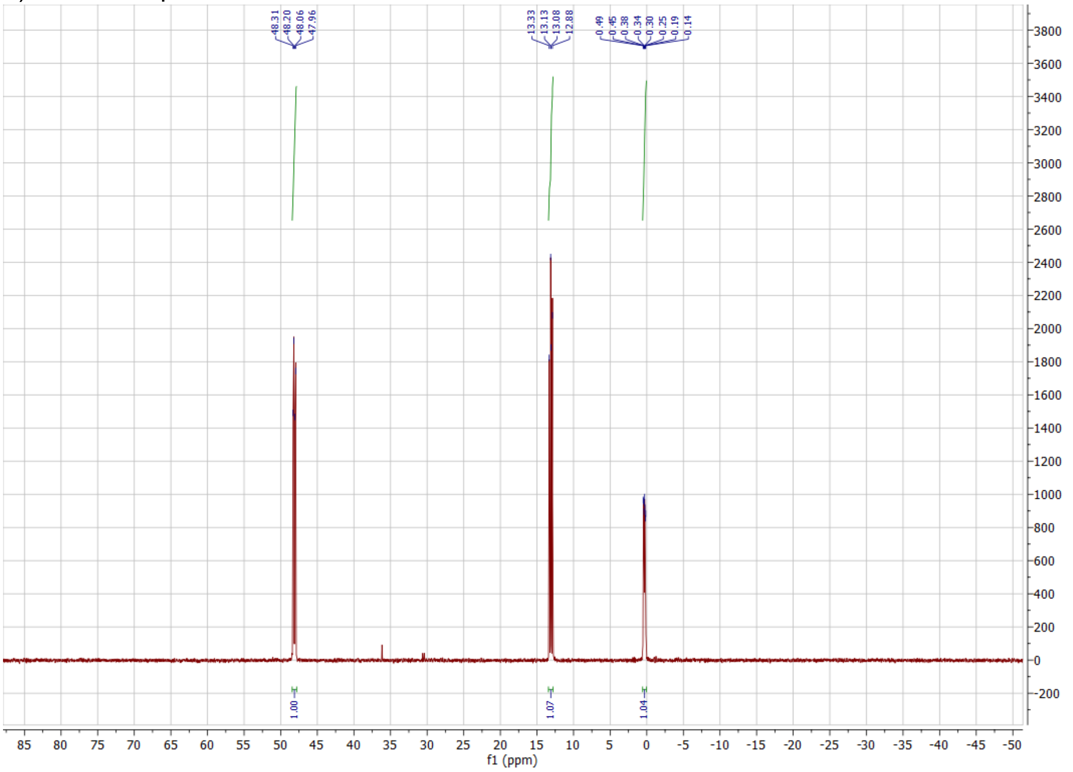

Model 1 (1,3-bis(4-(2-(4-methoxyphenyl)propan-2-yl)phenoxy)propan-2-ol)

a) ^{1}H NMR spectrum

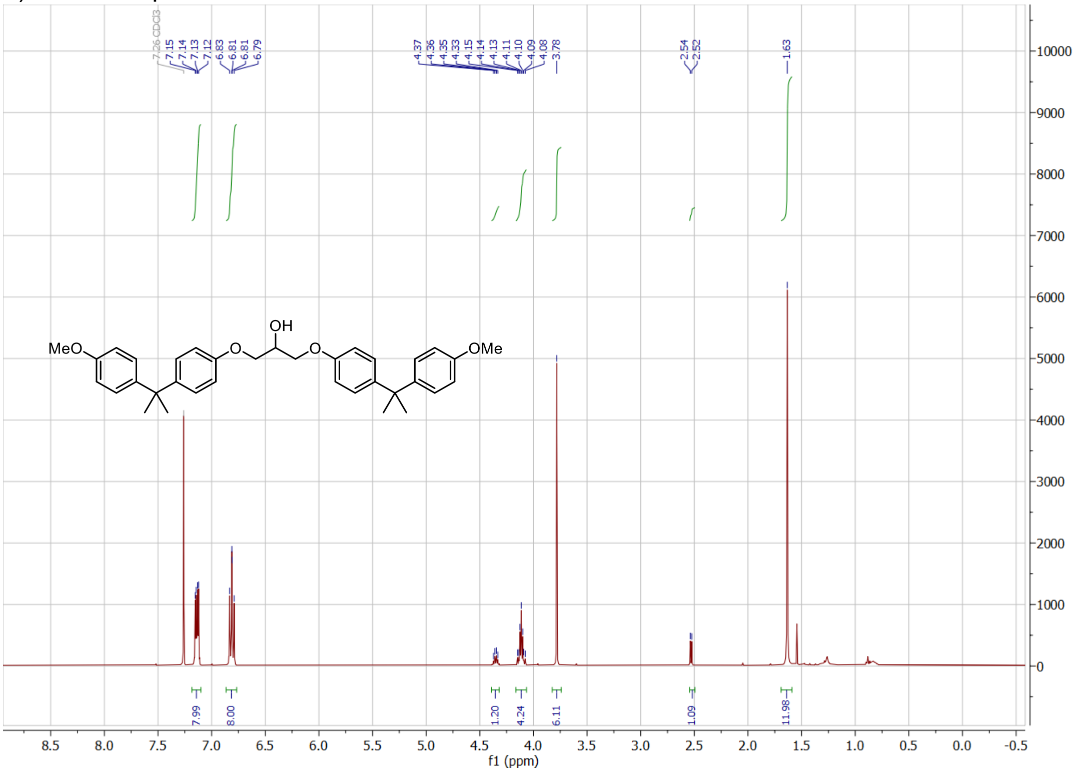

b) ^{13}C NMR spectrum

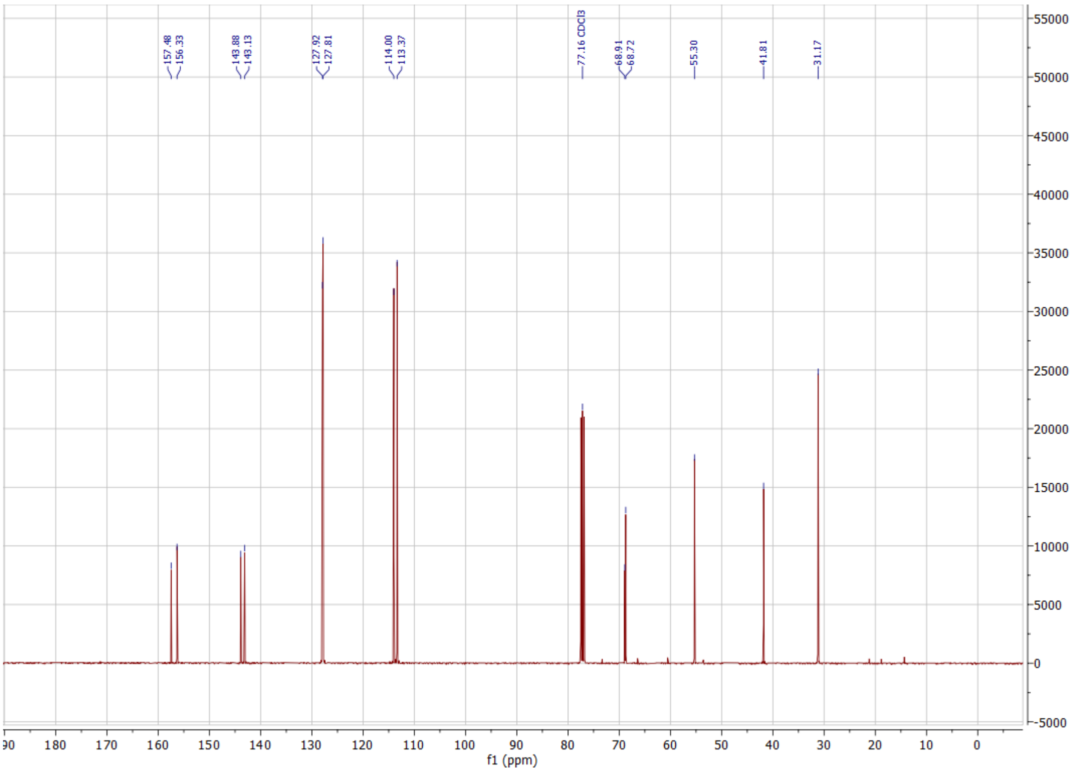

2-((4-(2-(4-methoxyphenyl)propan-2- yl)phenoxy)methyl)oxirane

a) ^{1}H NMR spectrum

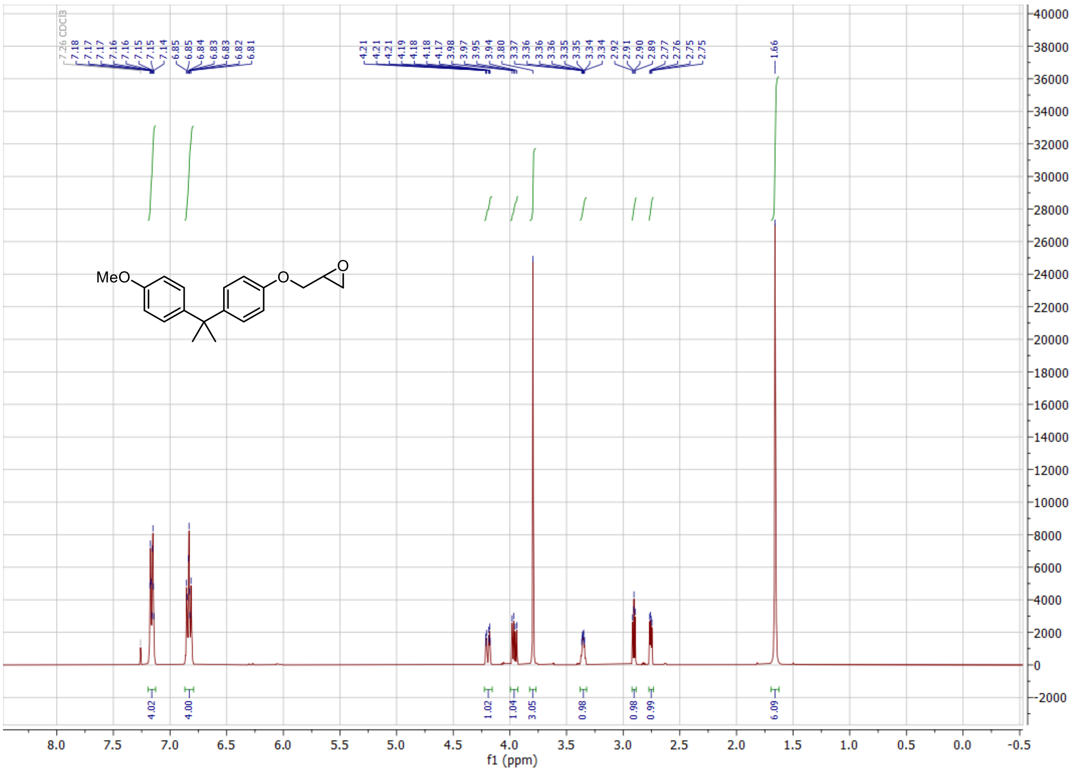

b) ^{13}C NMR spectrum

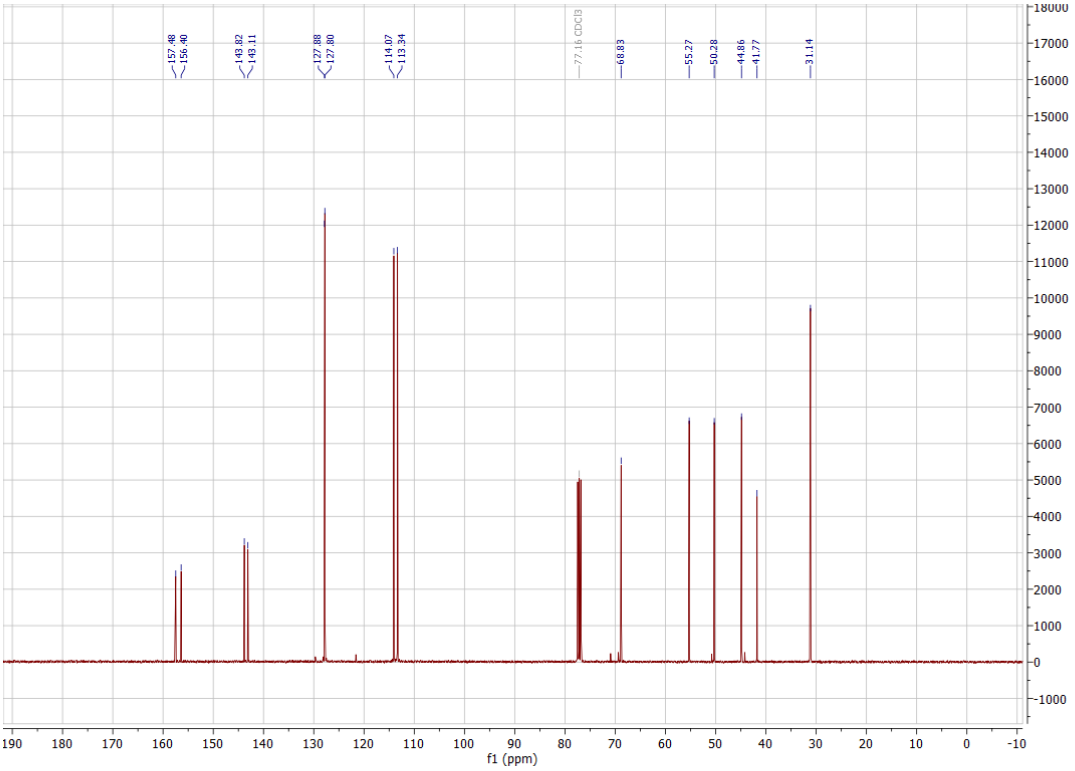

Model 2 (1-((cyclohexylmethyl)amino)-3-(4-(2-(4-methoxyphenyl)propan-2-yl)phenoxy)propan-2-ol)

a) ^{1}H NMR spectrum

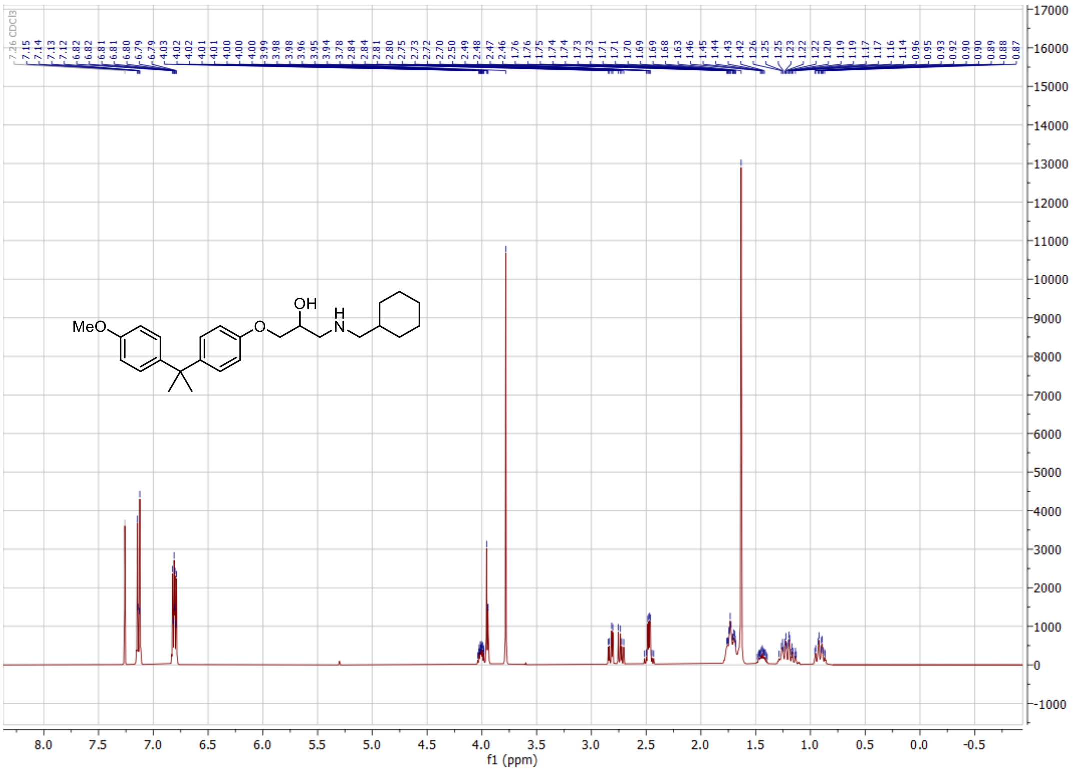

b) ^{13}C NMR spectrum

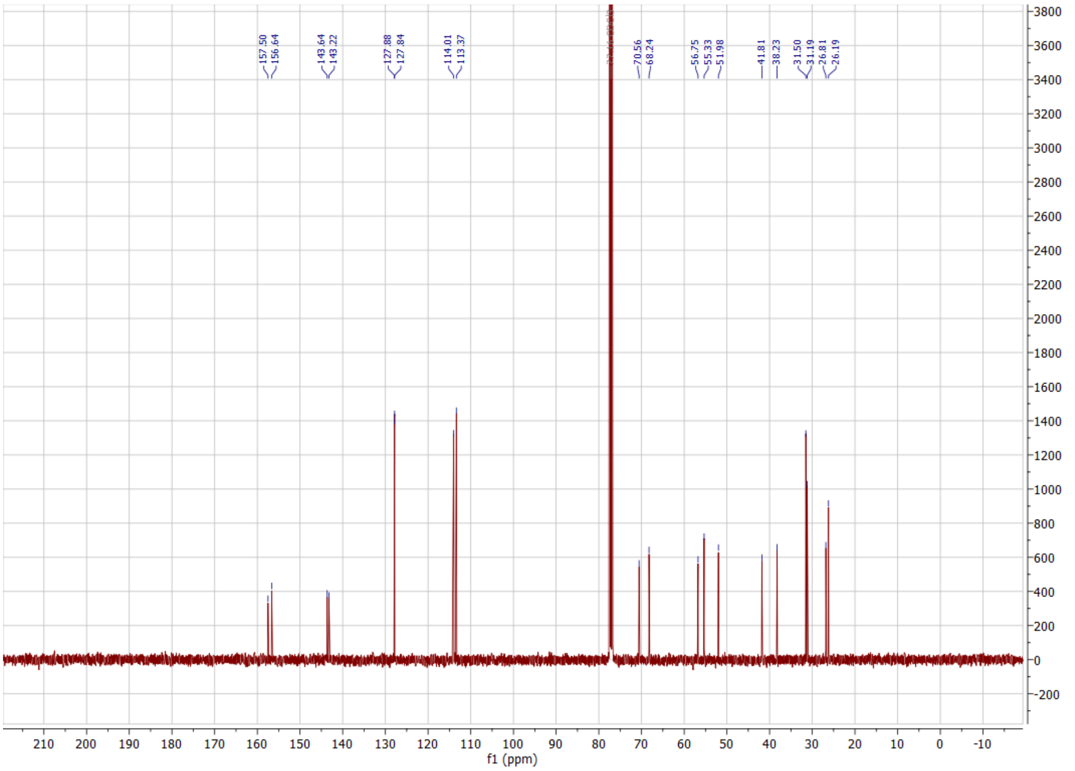

1-((1,3-bis(4-(2-(4- methoxyphenyl)propan-2-yl)phenoxy)propan-2-yl)oxy)butan-2-one

a) ^{1}H NMR spectrum

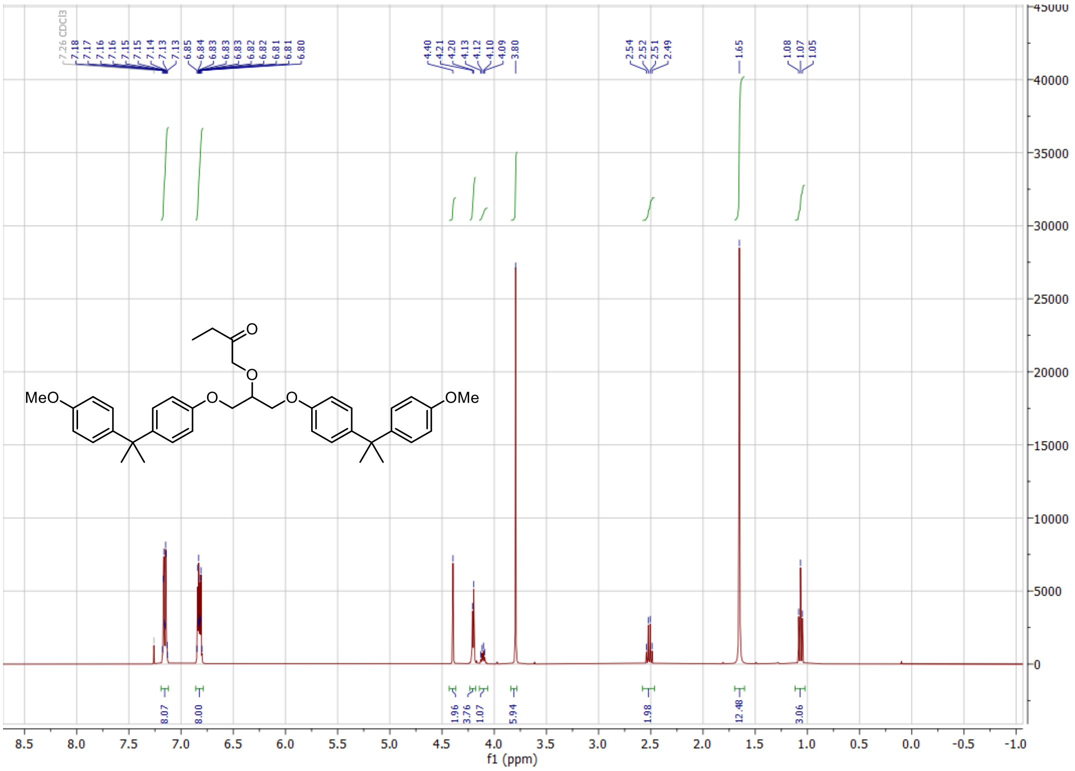

b) ^{13}C NMR spectrum

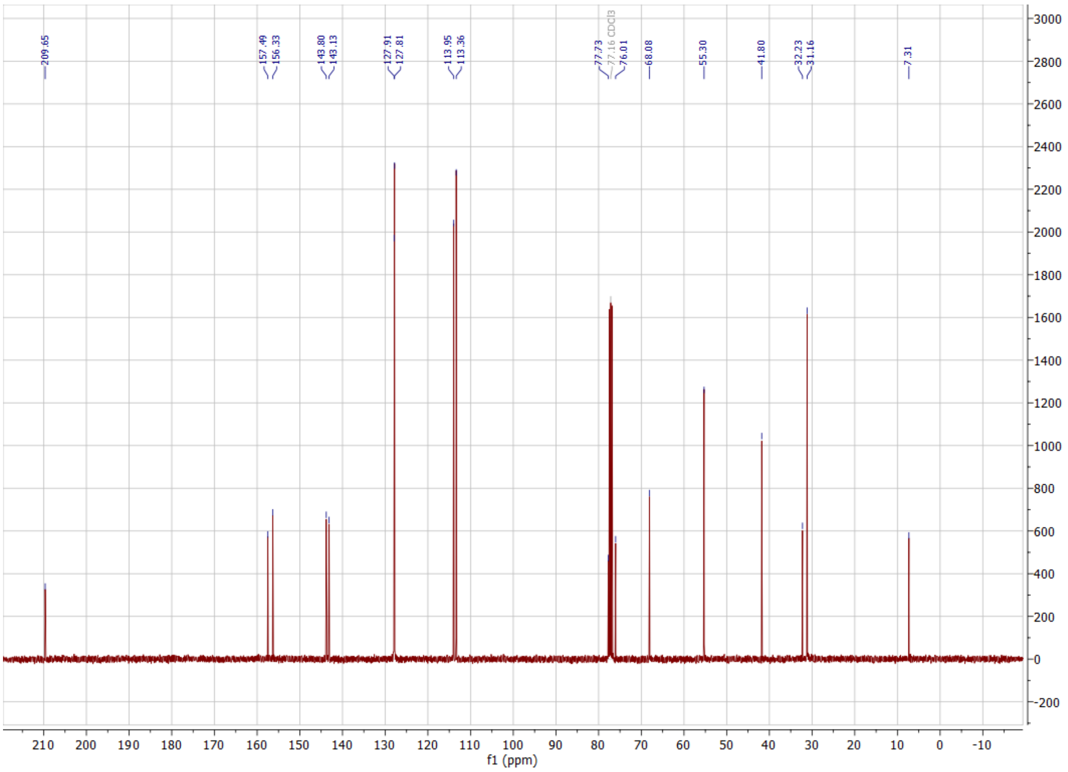

Model 3 (1-(diethylamino)-3-(4-(2-(4-methoxyphenyl)propan-2-yl)phenoxy)propan-2-ol)

a) ^{1}H NMR spectrum

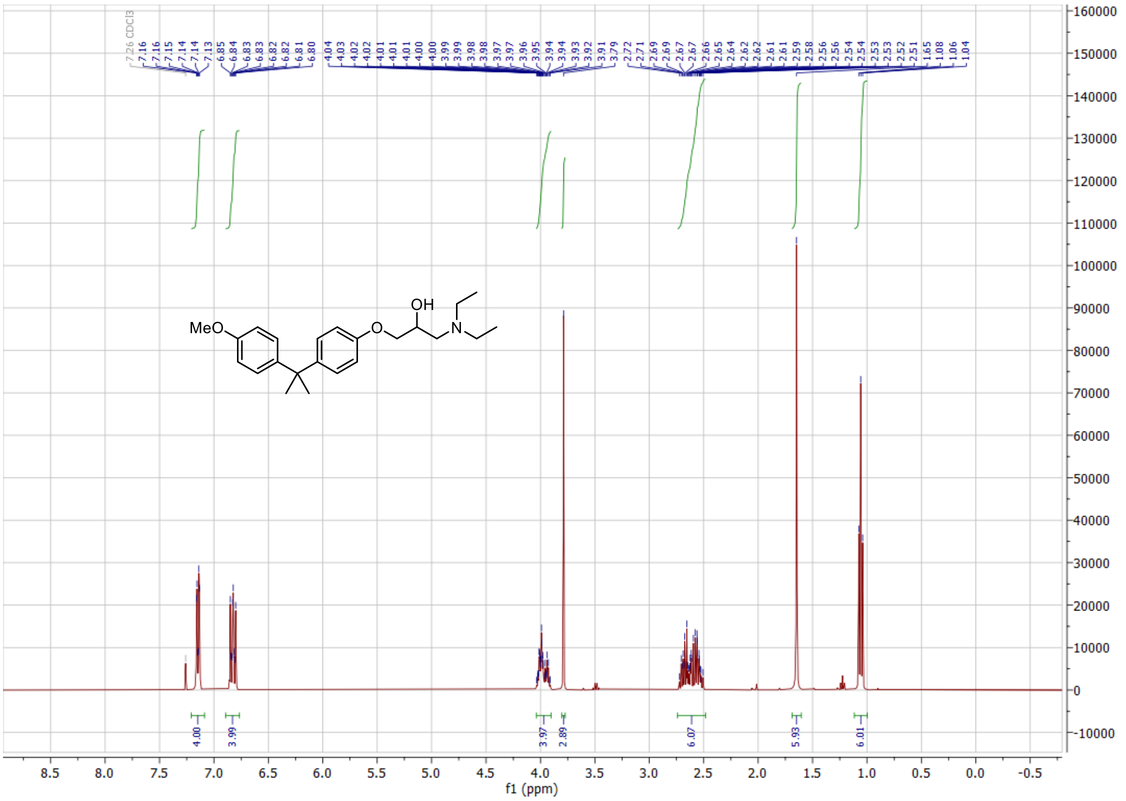

b) ^{13}C NMR spectrum

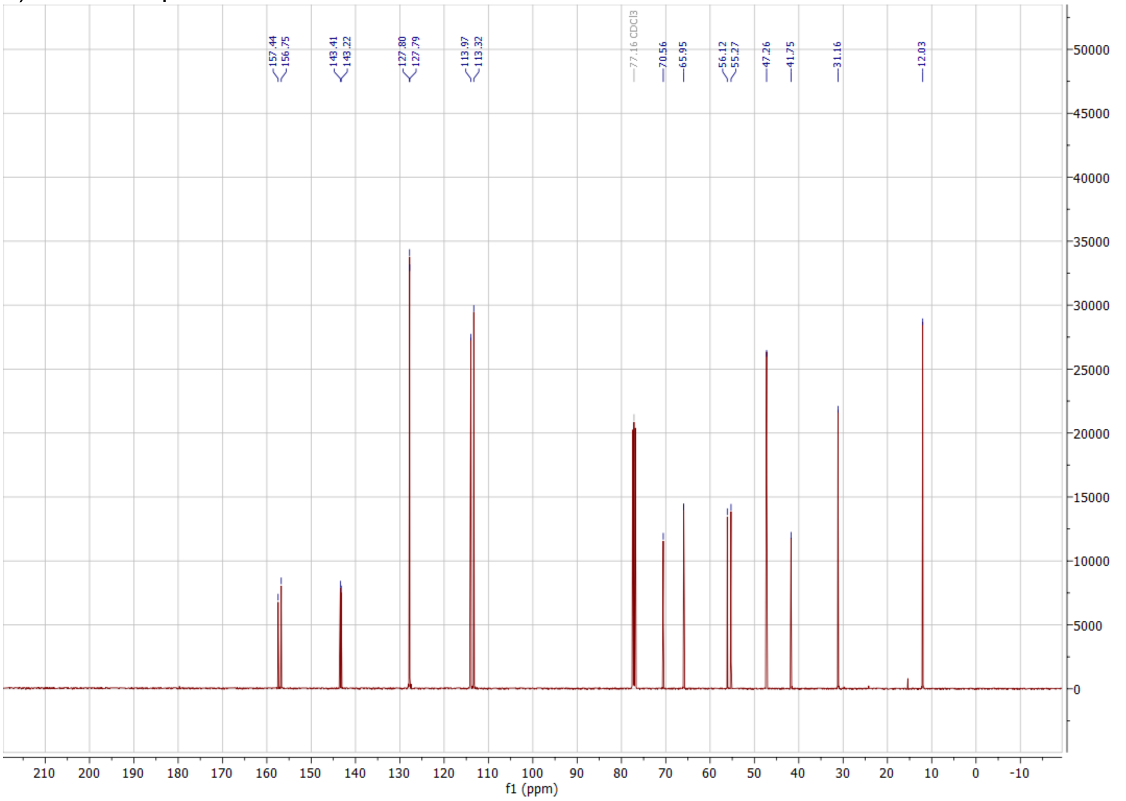

Model 4 (1-((1,3-bis(4-(2-(4-methoxyphenyl)propan-2-yl)phenoxy)propan-2-yl)oxy)butan-2-ol)

a) ^{1}H NMR spectrum

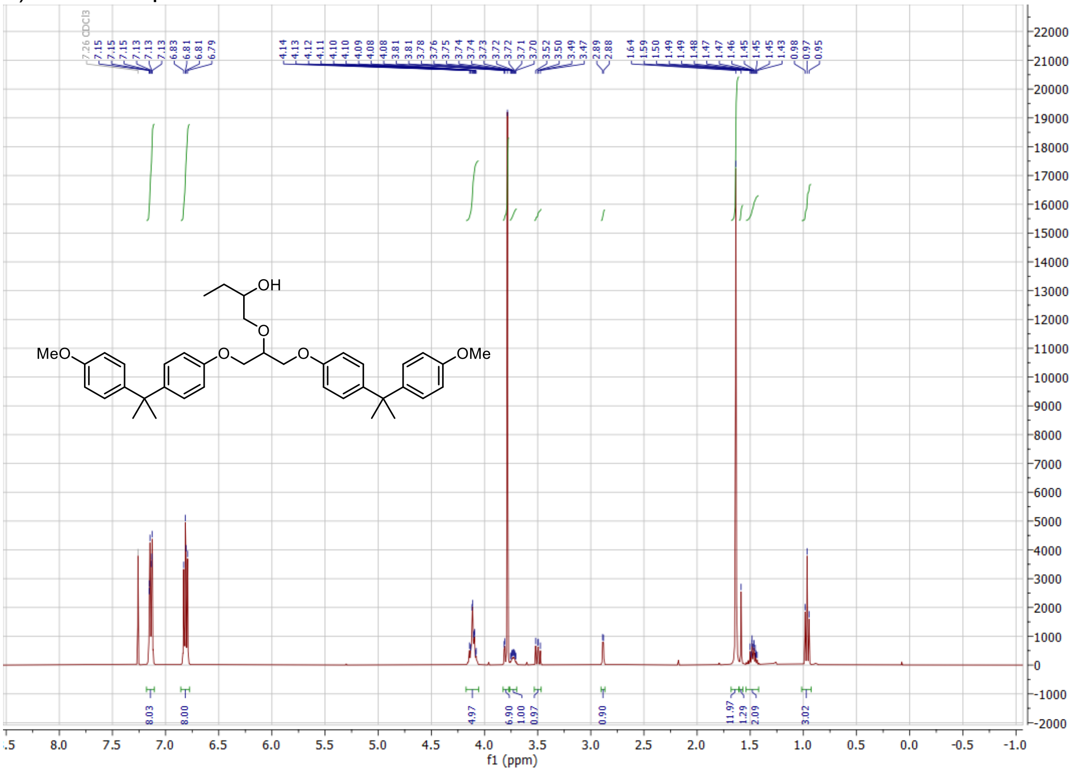

b) ^{13}C NMR spectrum

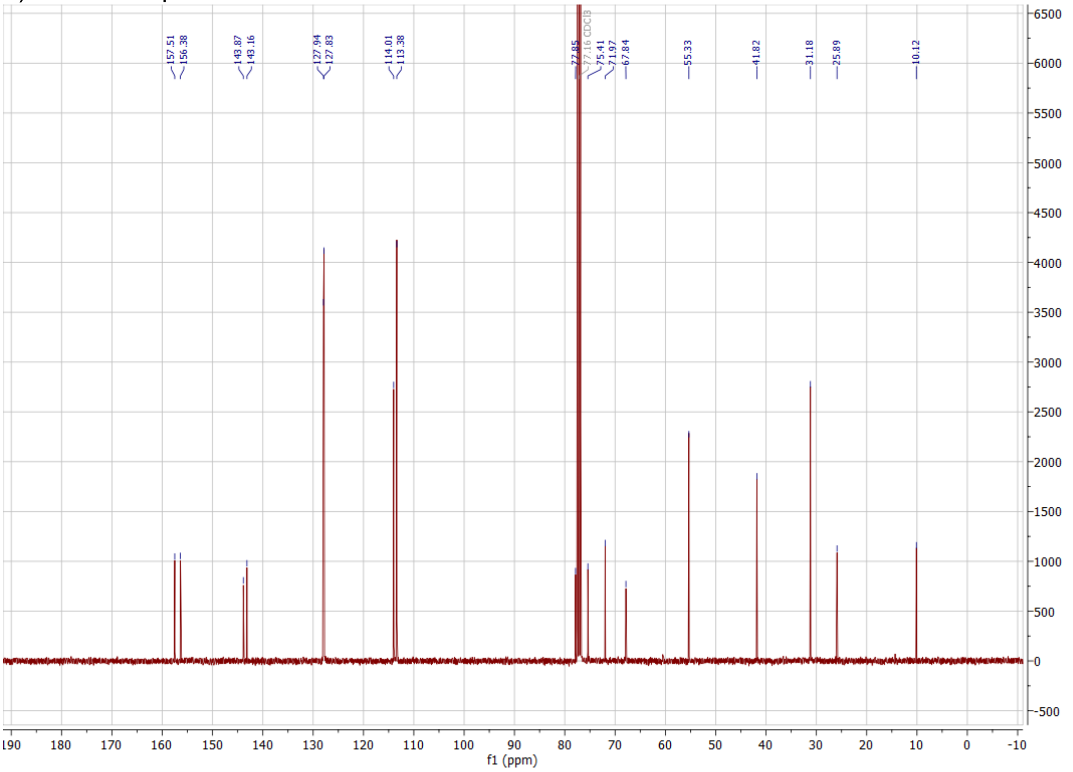

Model 5 (1,3-bis(4-(2-(4-methoxyphenyl)propan-2-yl)phenoxy)propan-2-ol)

a) ^{1}H NMR spectrum

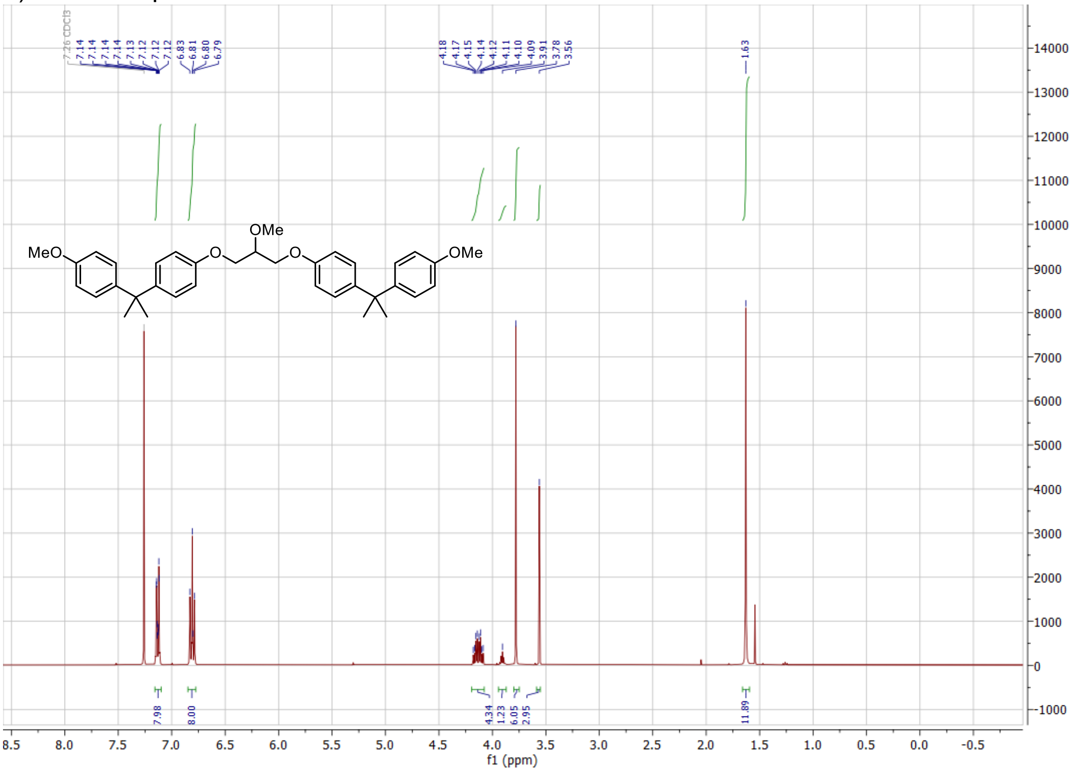

b) ^{13}C NMR spectrum

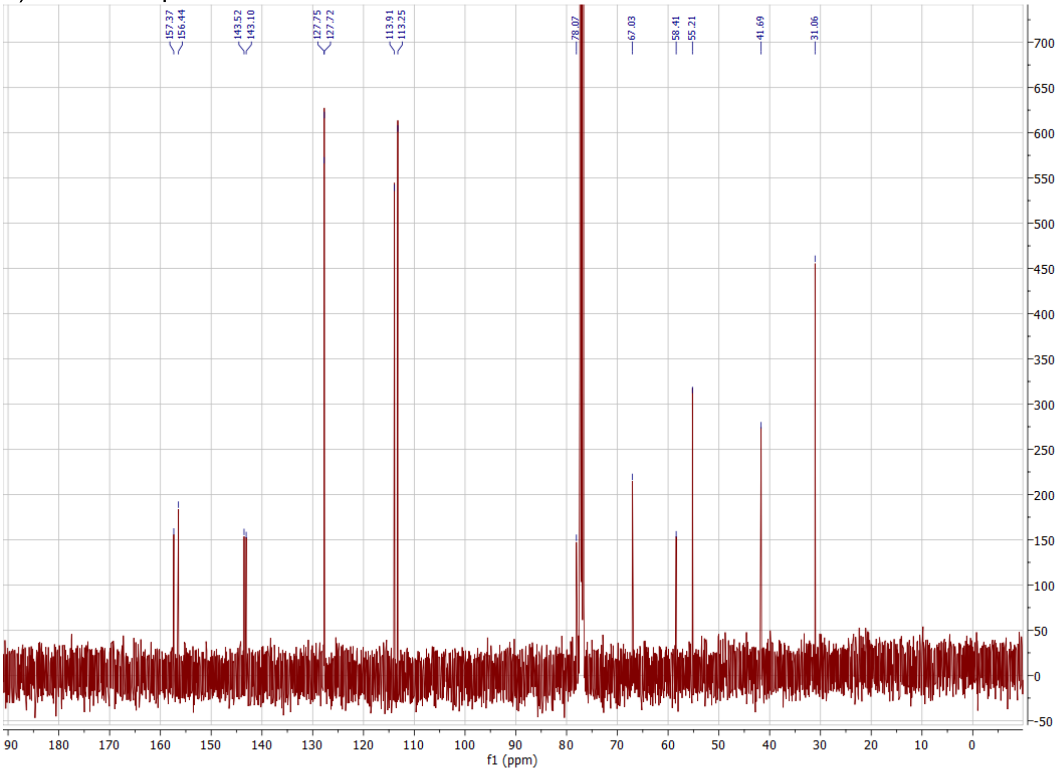

Ketone III (1-(4-(2-(4-methoxyphenyl)propan-2-yl)phenoxy)propan-2-one)

a) ^{1}H NMR spectrum

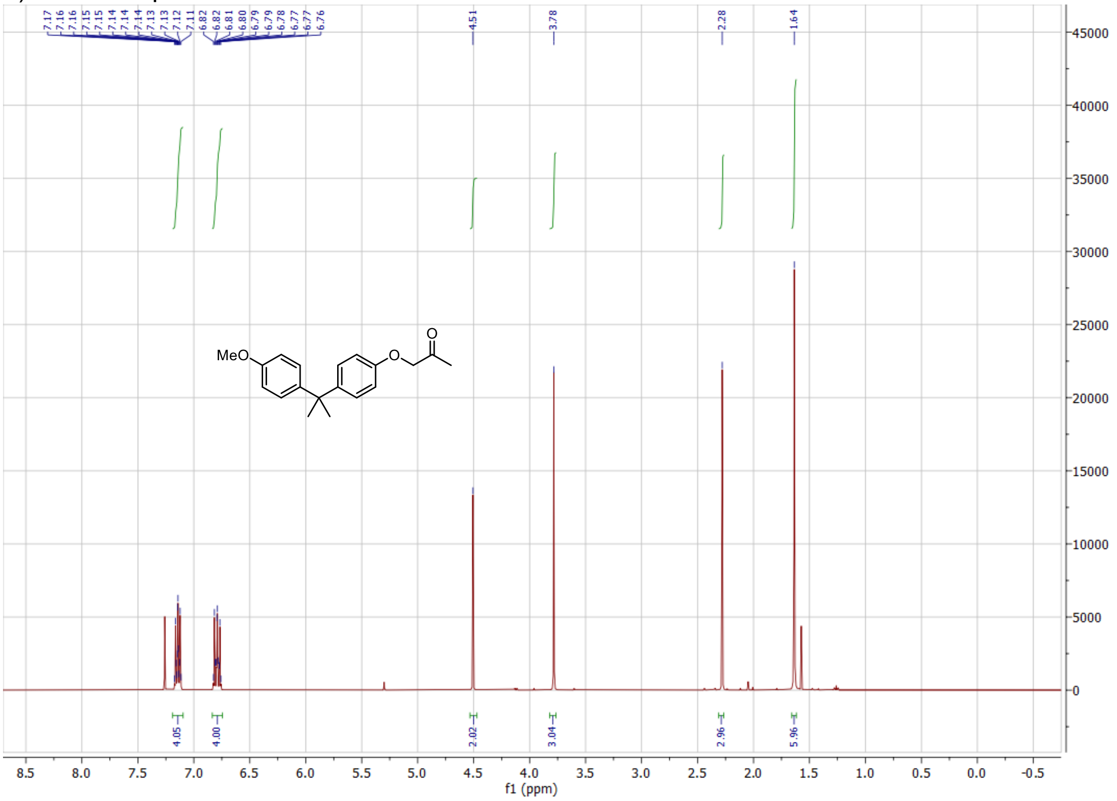

b) ^{13}C NMR spectrum

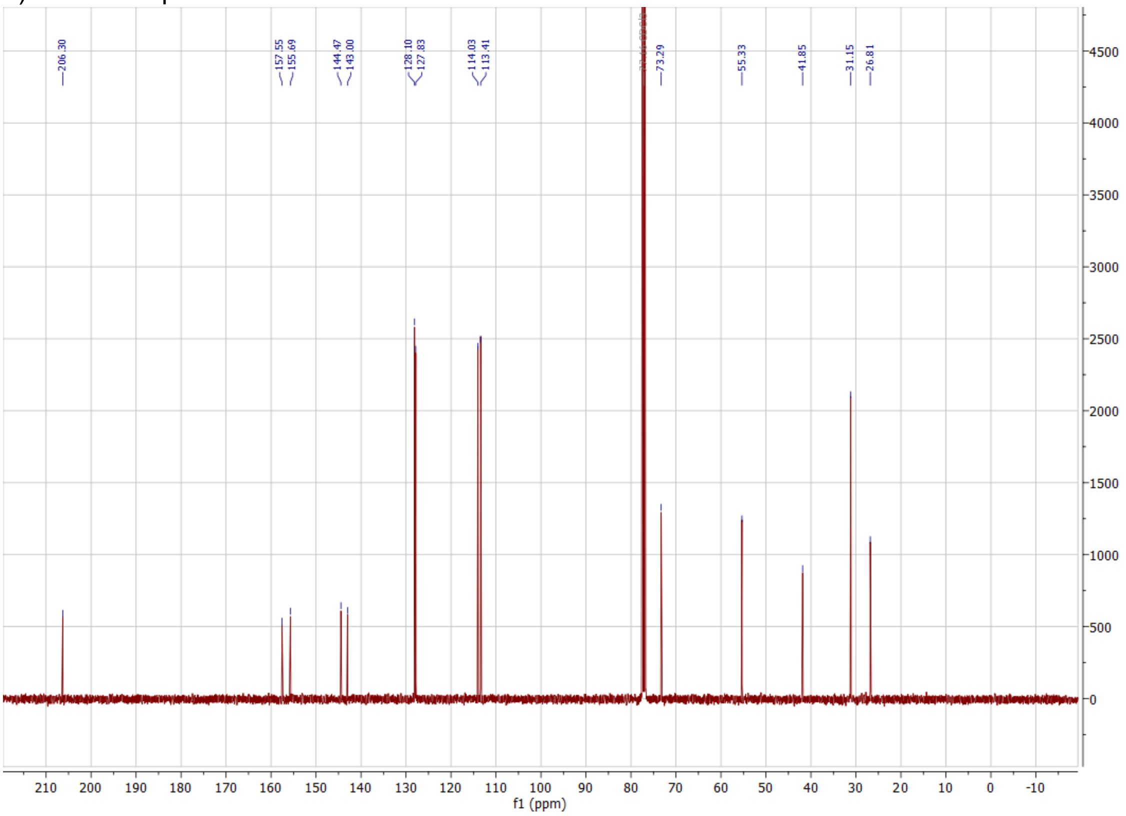

Me-BPS (4-((4-methoxyphenyl)sulfonyl)phenol)

a) ^{1}H NMR spectrum

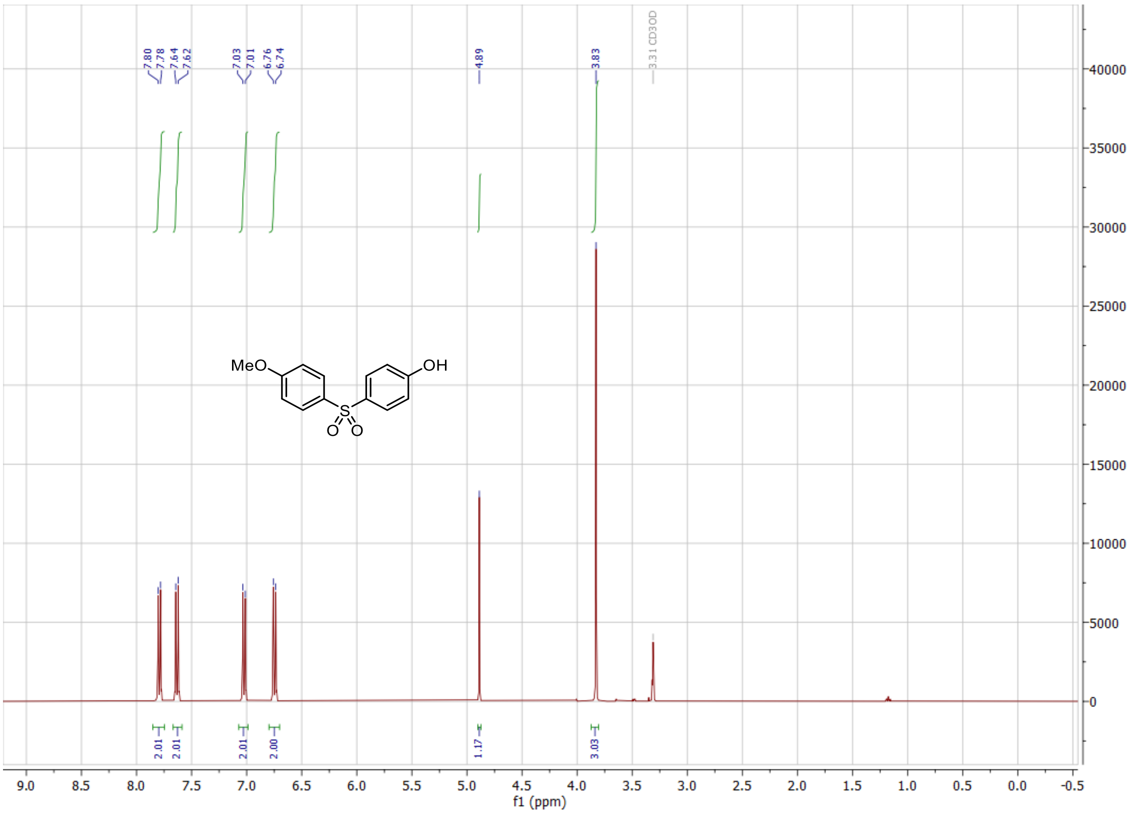

b) ^{13}C NMR spectrum

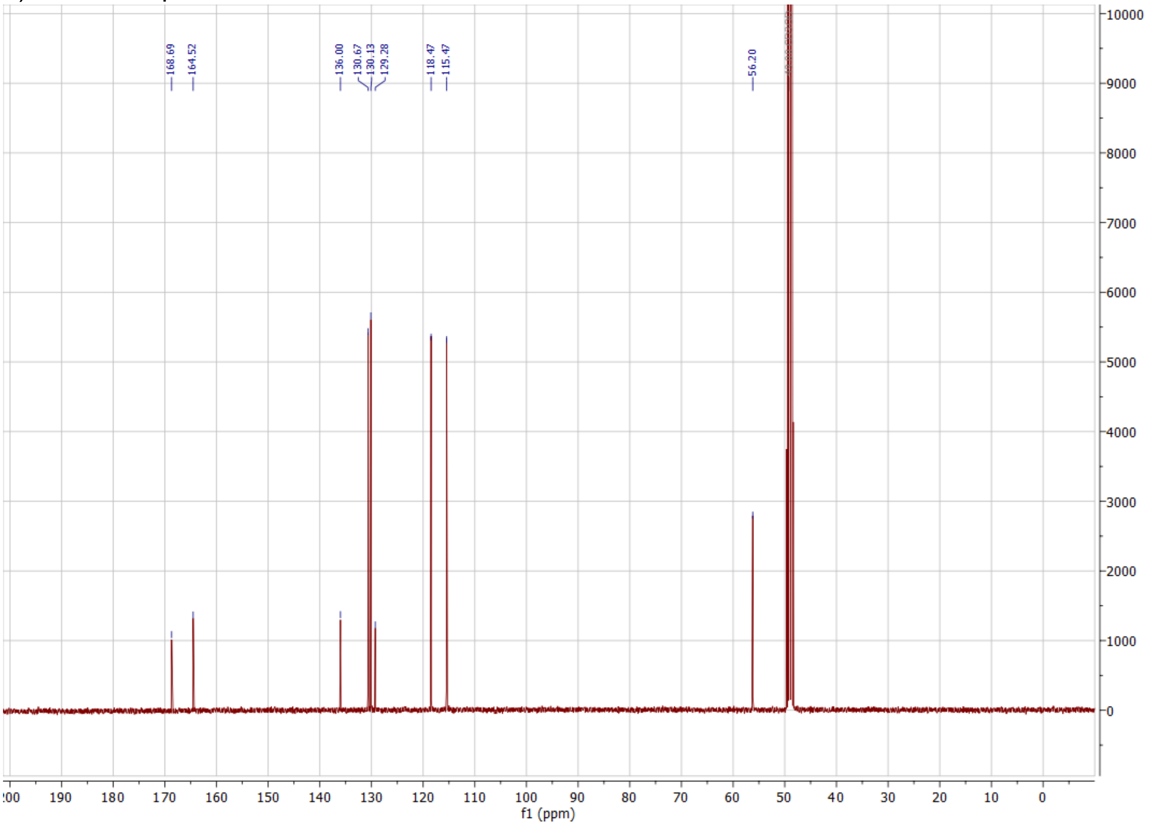

Model 6 (1,3-bis(4-((4-methoxyphenyl)sulfonyl)phenoxy)propan-2-ol)

a) ^{1}H NMR spectrum

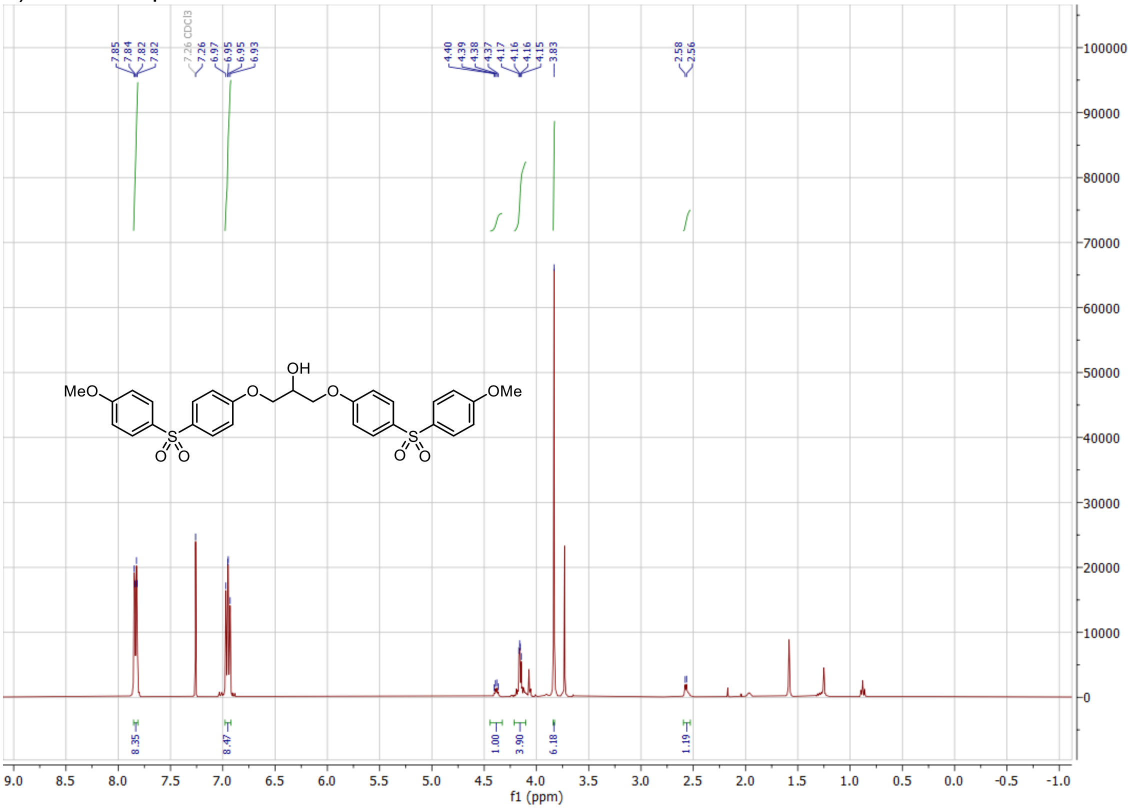

b) ^{13}C NMR spectrum

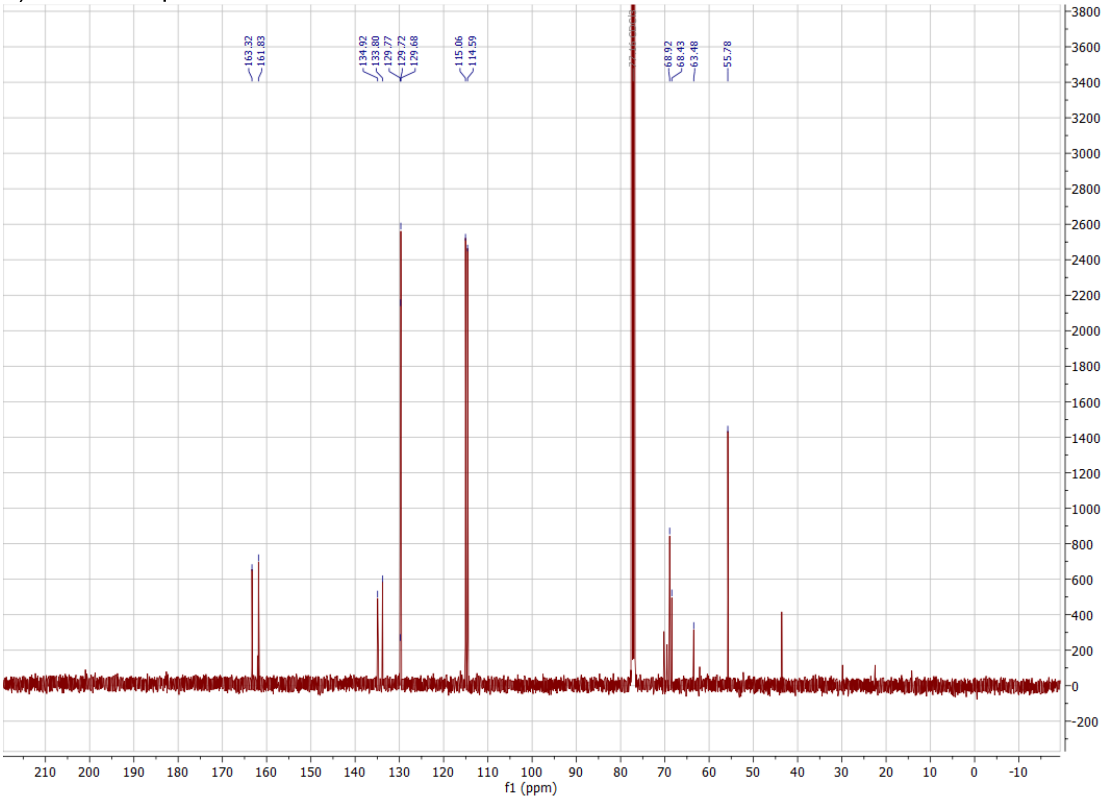

4,4'-methylenebis(5-isopropyl-2-methylphenol)

a) ^{1}H NMR spectrum

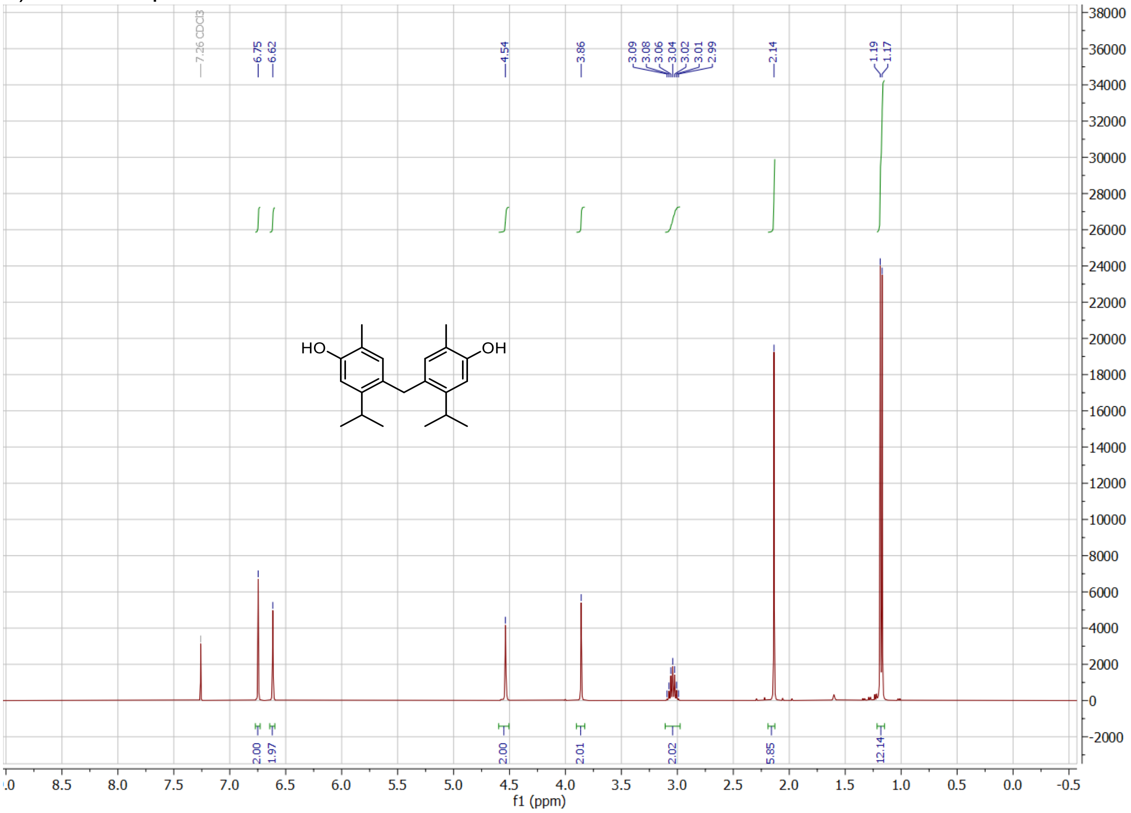

b) ^{13}C NMR spectrum

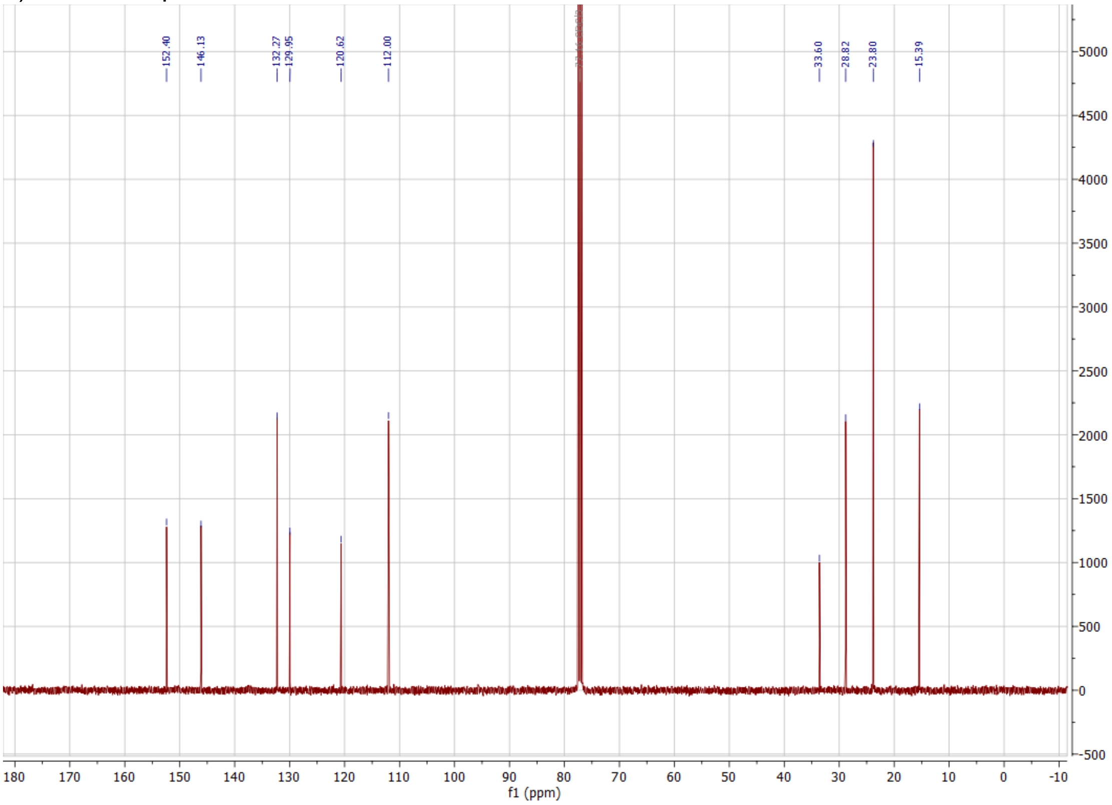

Model 7 (1,3-bis(5-isopropyl-4-(2-isopropyl-4-methoxy-5-methylbenzyl)-2-methylphenoxy)propan-2-ol)

a) ^{1}H NMR spectrum

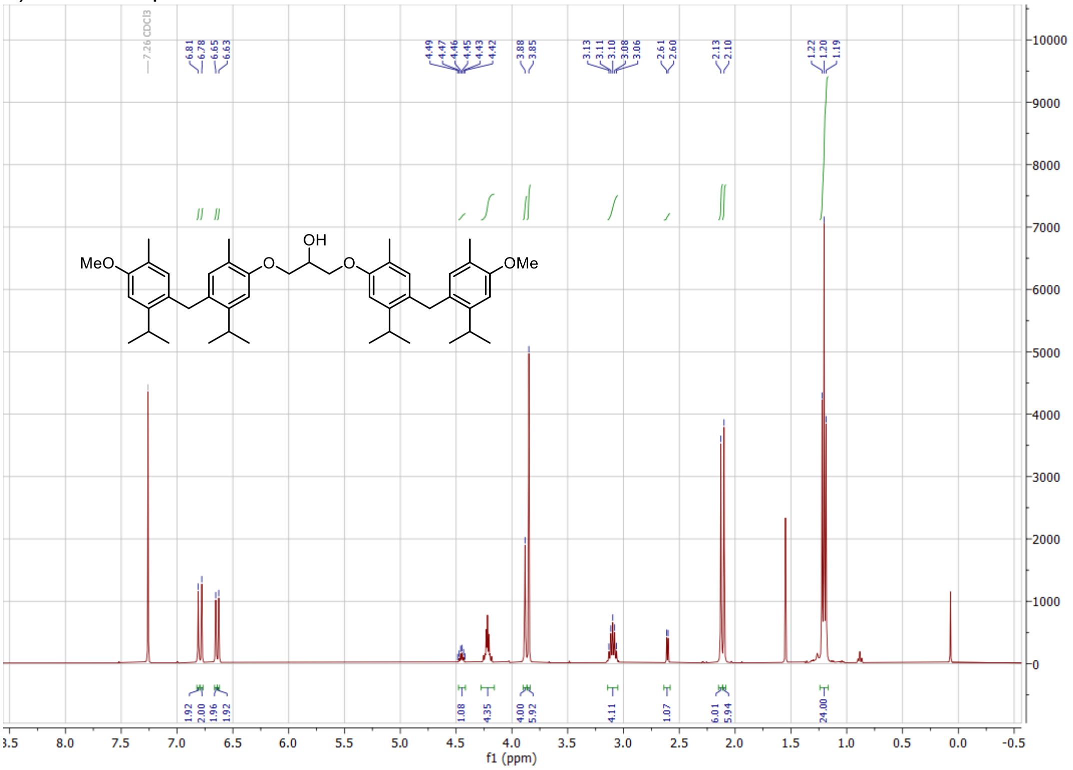

b) ^{13}C NMR spectrum

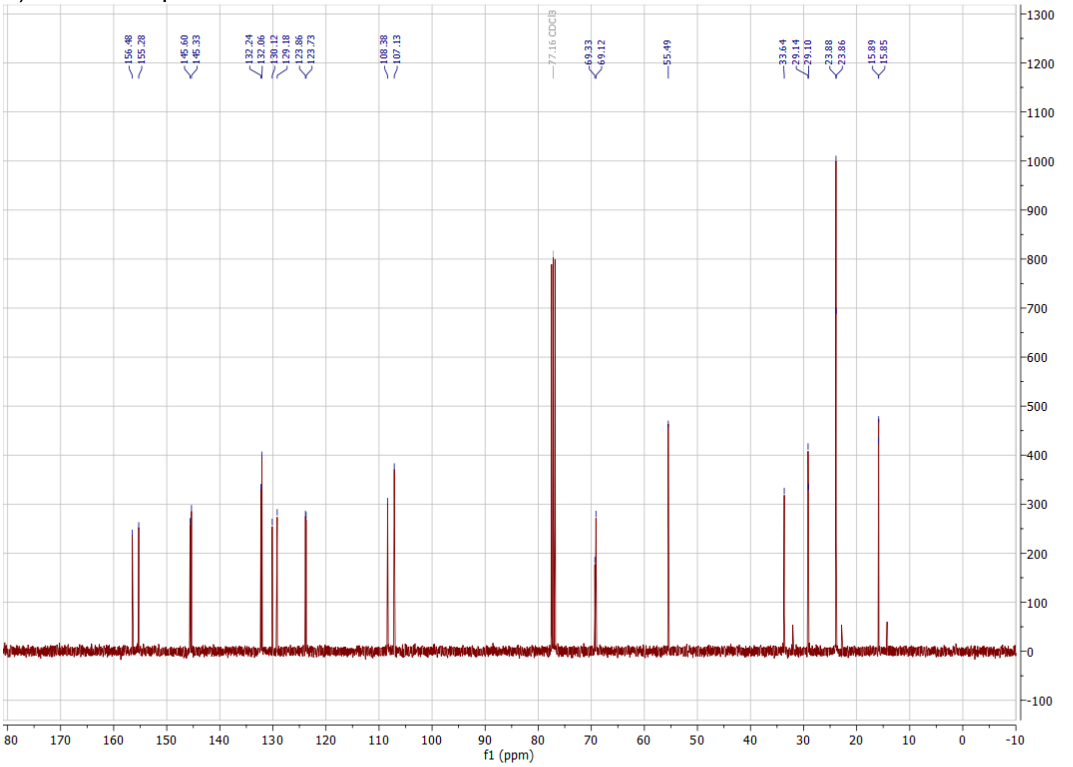

4-(3,4-dimethoxybenzyl)-2-methoxyphenol

a) ^{1}H NMR spectrum

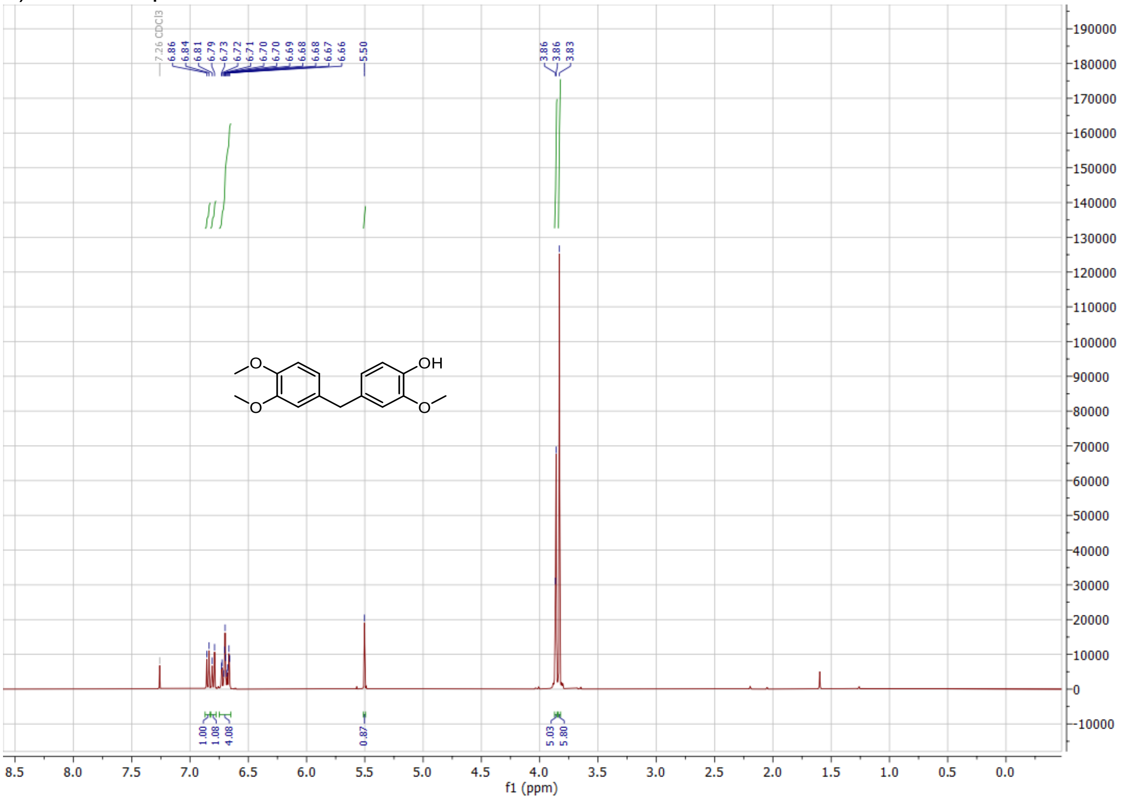

b) ^{13}C NMR spectrum

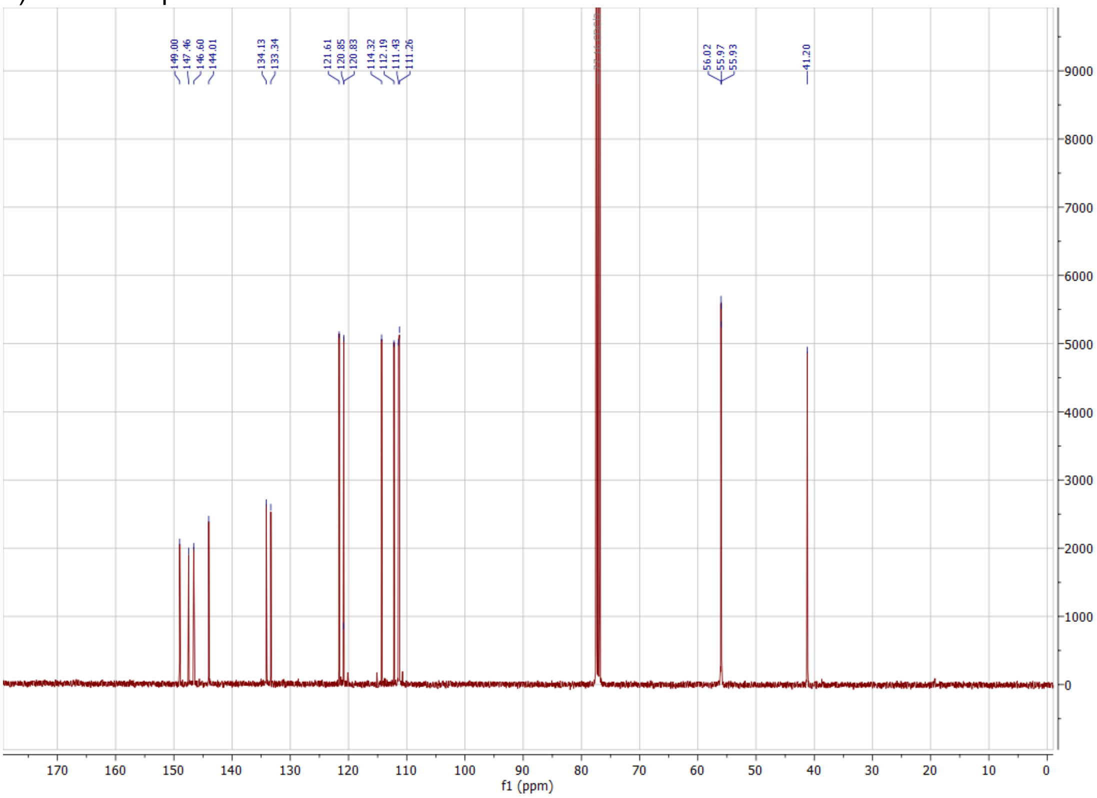

Model 8 1,3-bis(4-(3,4-dimethoxybenzyl)-2-methoxyphenoxy)propan-2-ol

a) ^{1}H NMR spectrum

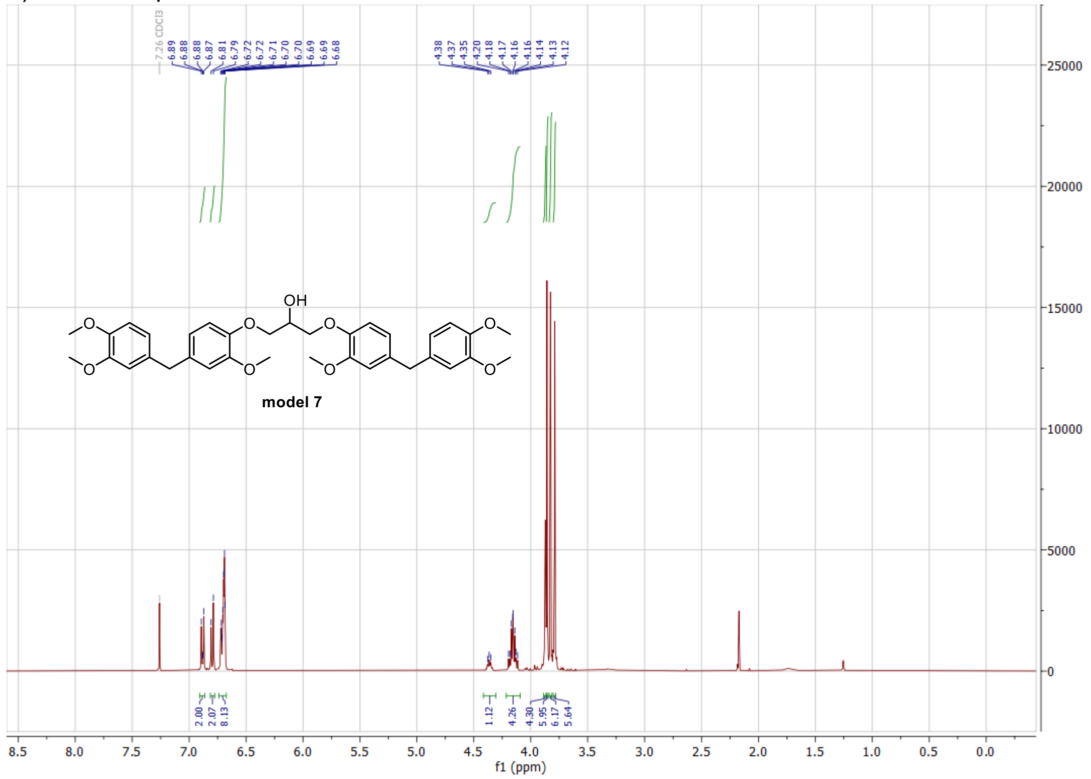

b) ^{13}C NMR spectrum

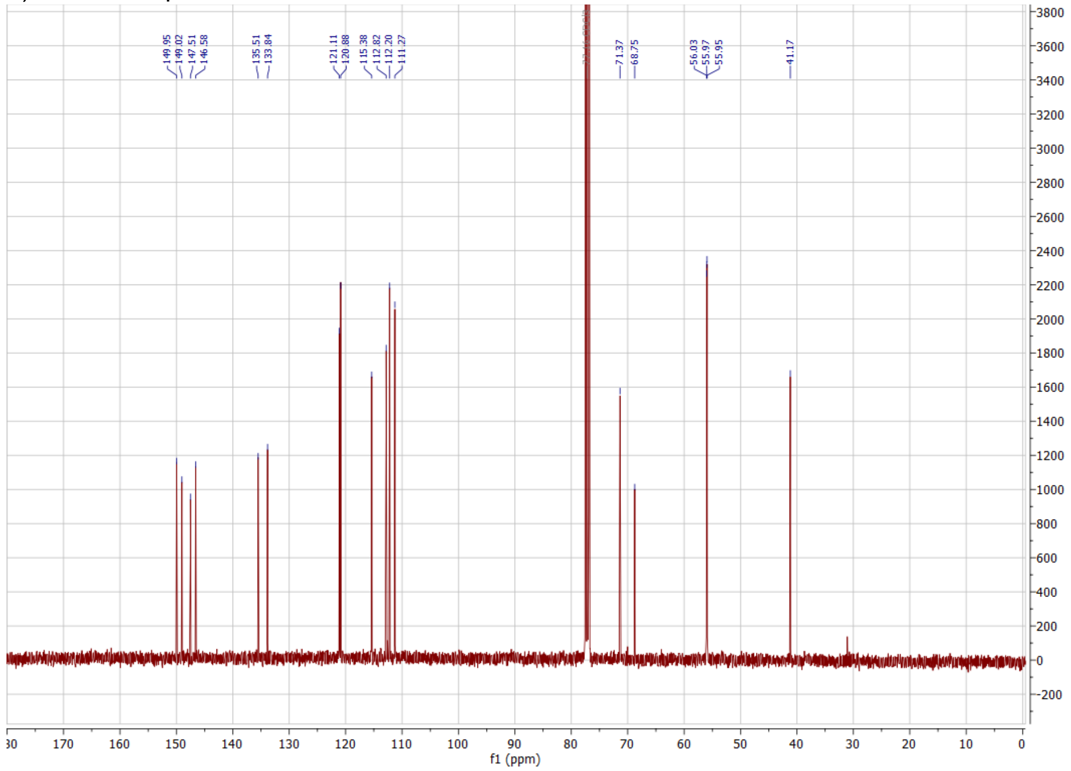

(5-(methoxymethyl)furan-2-yl)methanol

a) ^{1}H NMR spectrum

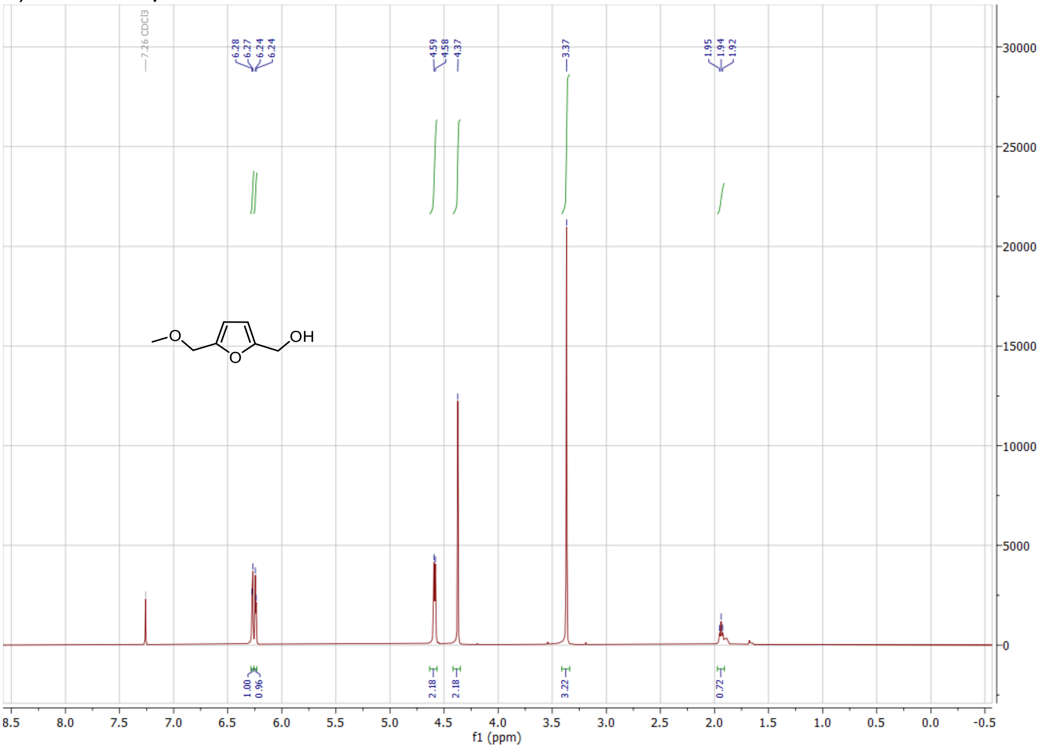

b) ^{13}C NMR spectrum

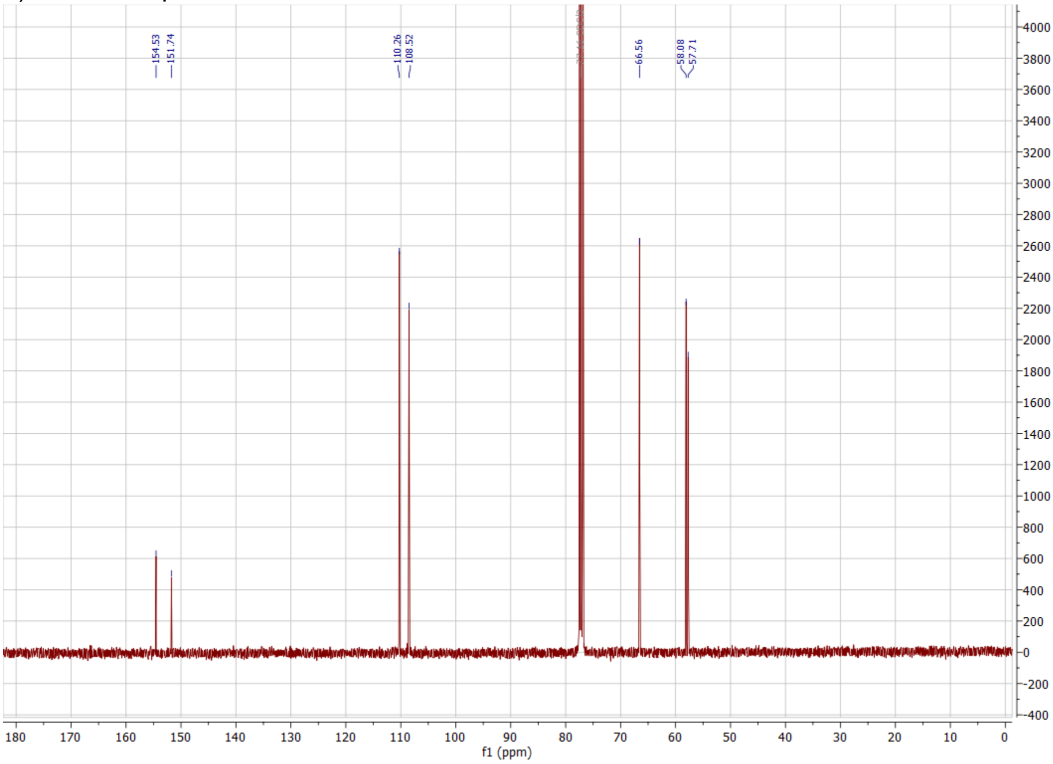

Model 9 1,3-bis((5-(methoxymethyl)furan-2-yl)methoxy)propan-2-ol

a) ^{1}H NMR spectrum

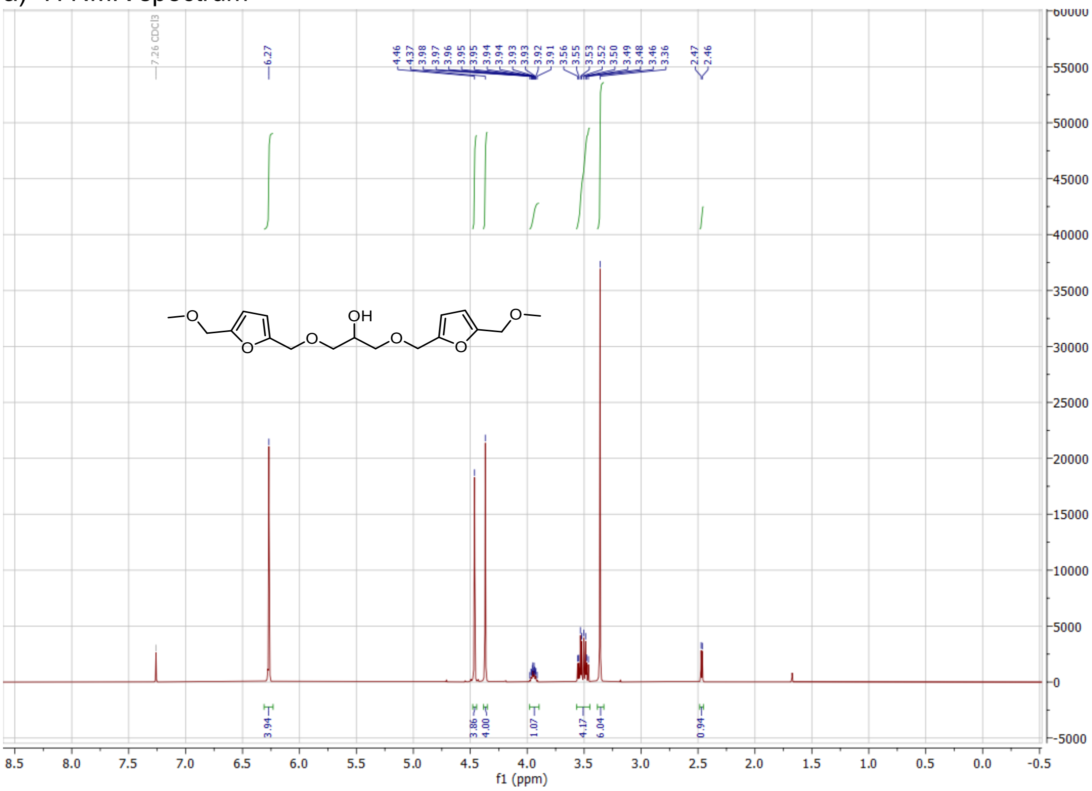

b) ^{13}C NMR spectrum

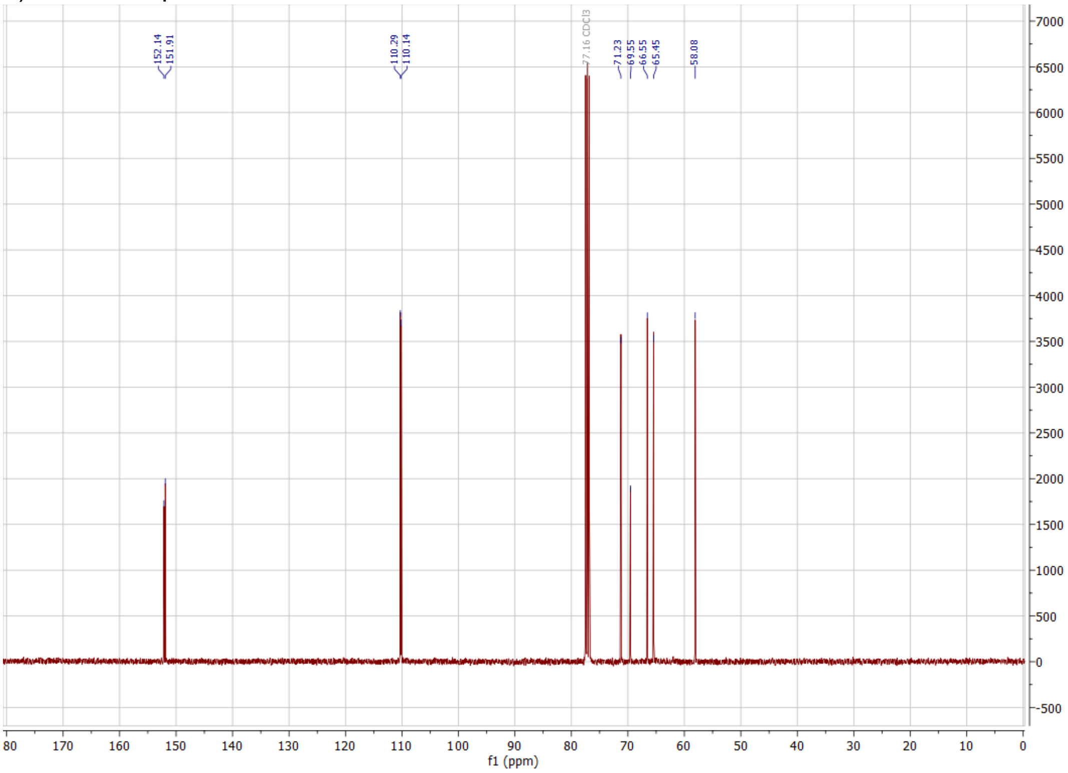

6.2 Spectra of Rest Fraction

a) ^1H NMR spectrum of deconstructed Airstone 760E/766H

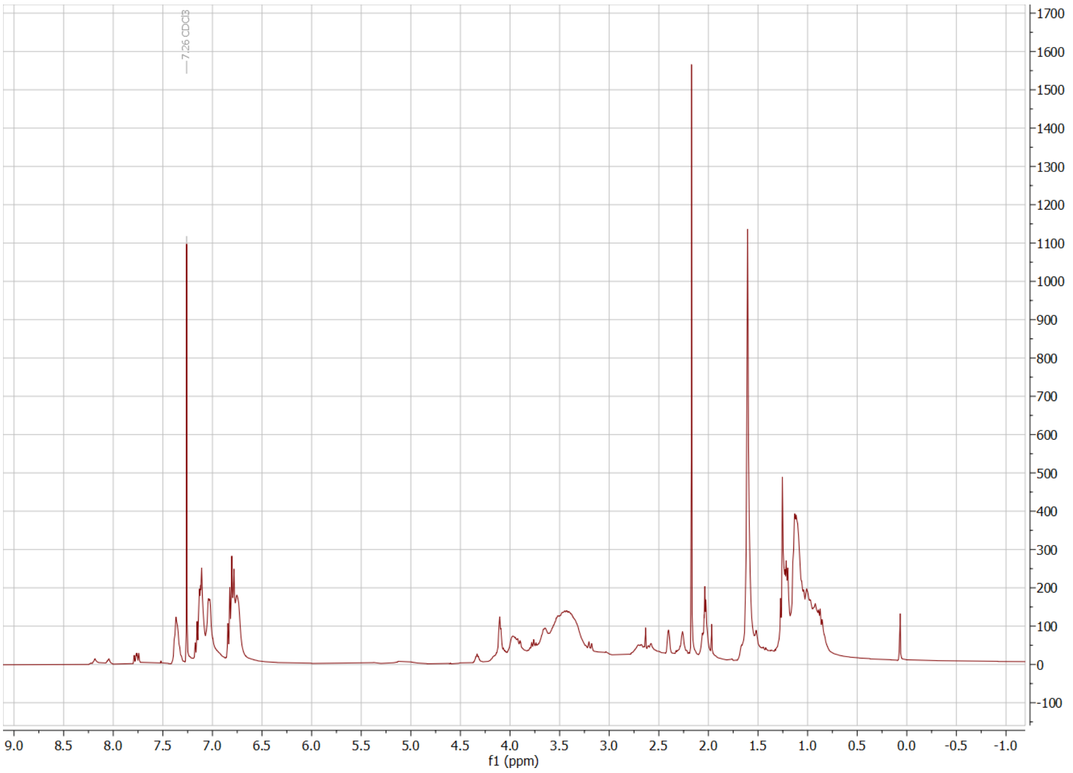

b) MALDI-TOF MS

Rest fraction of deconstructed Airstone 760E/766H

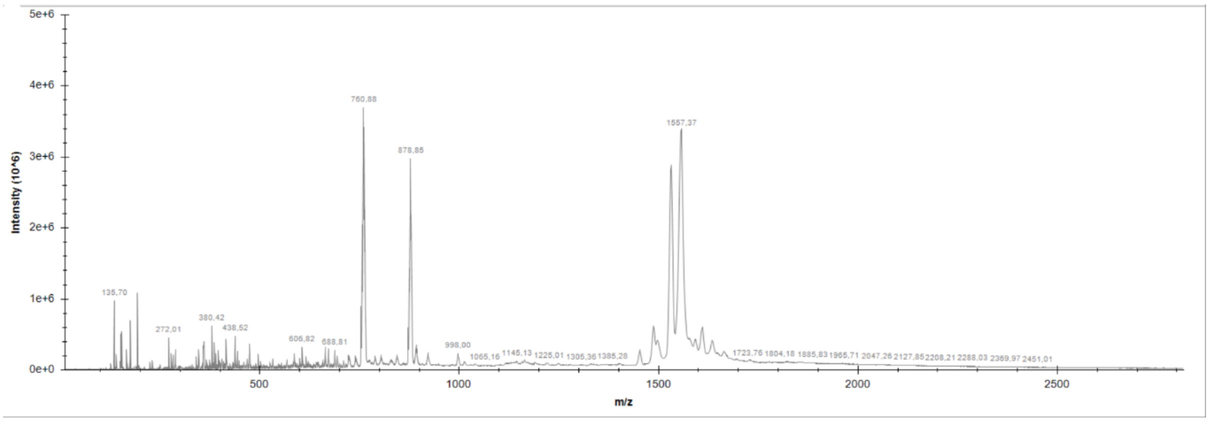

Rest fraction of deconstructed Airstone 760E/766H treated with silver triflate

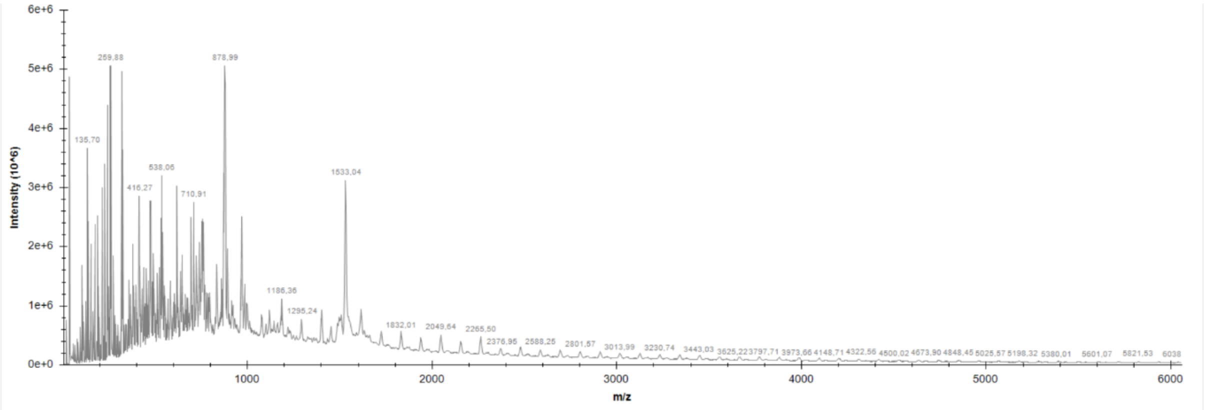

c) Infrared Spectra

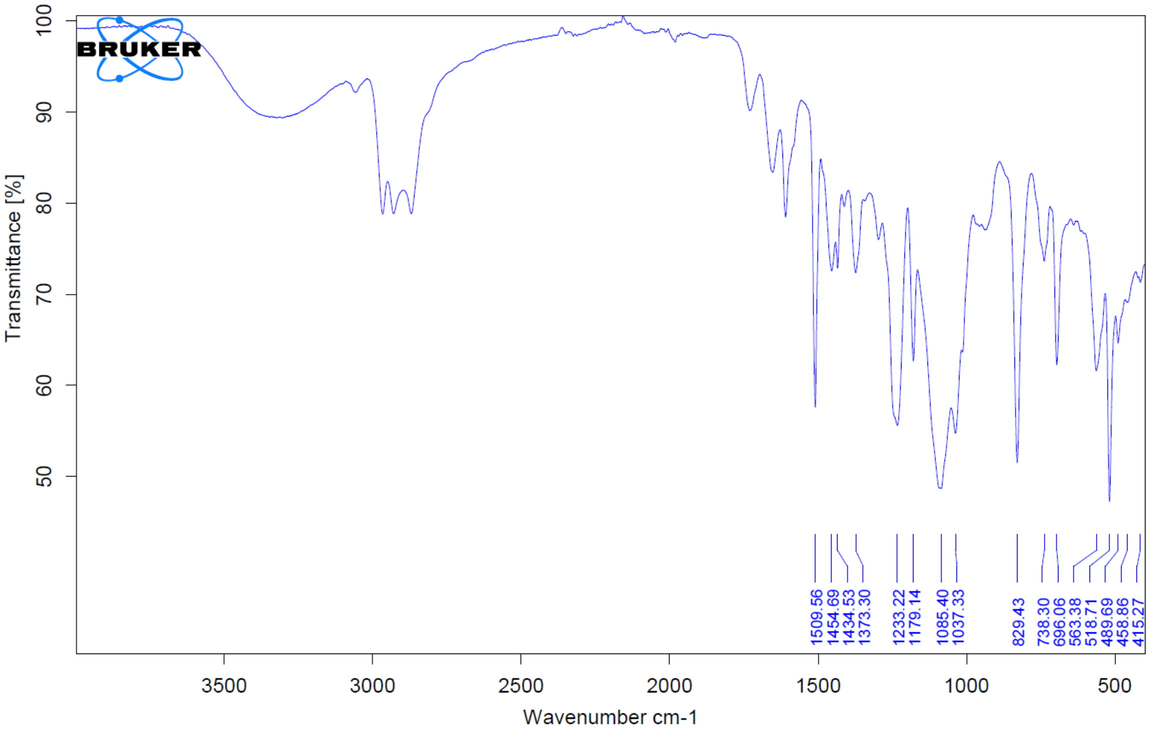

6.3 Characterisation of Recovered Fibers

6.3.1 Microscopic Images

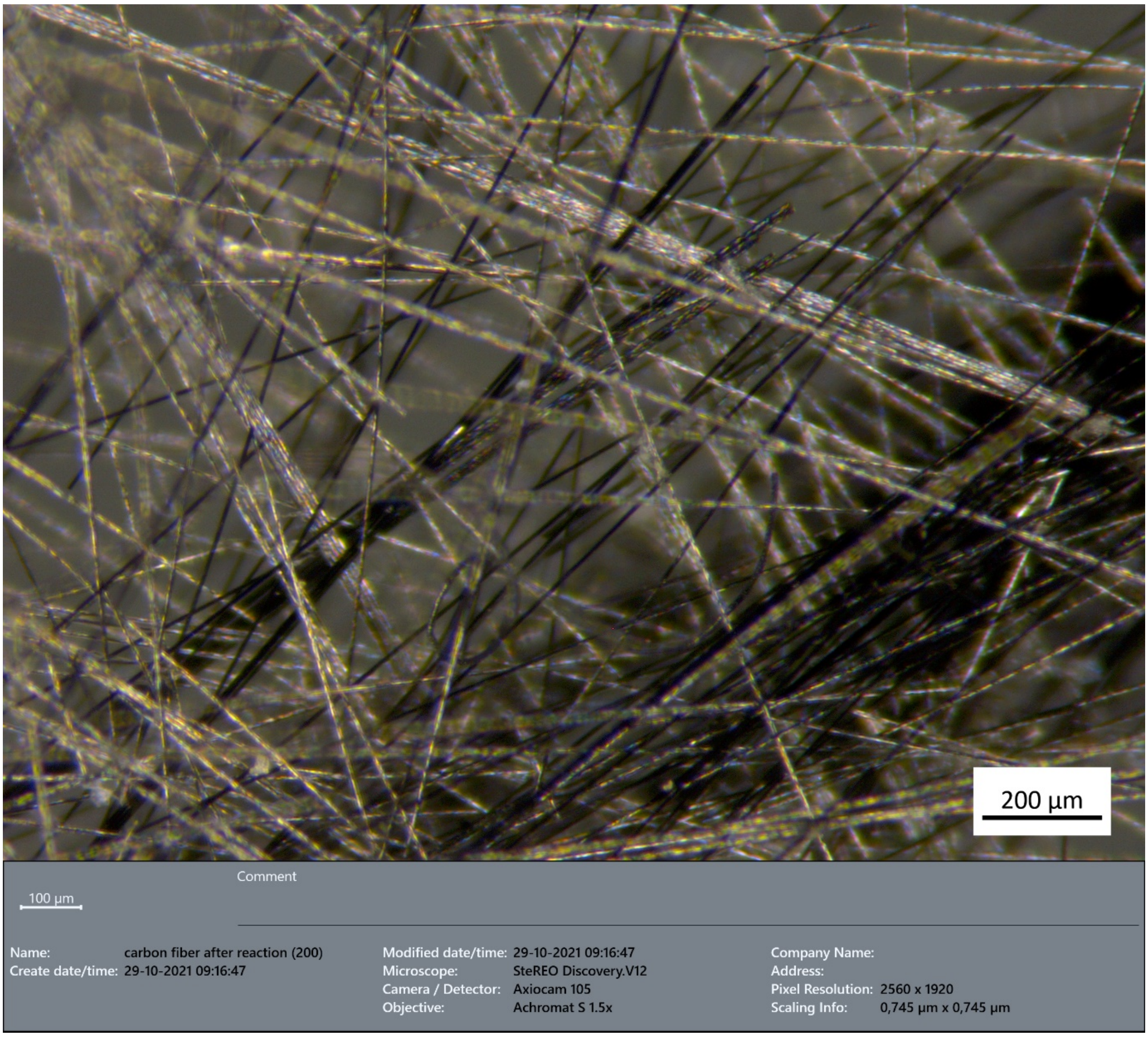

Fig. S12. Microscopic images of carbon fibers recovered from landfilled epoxy composite recovered from landfill.

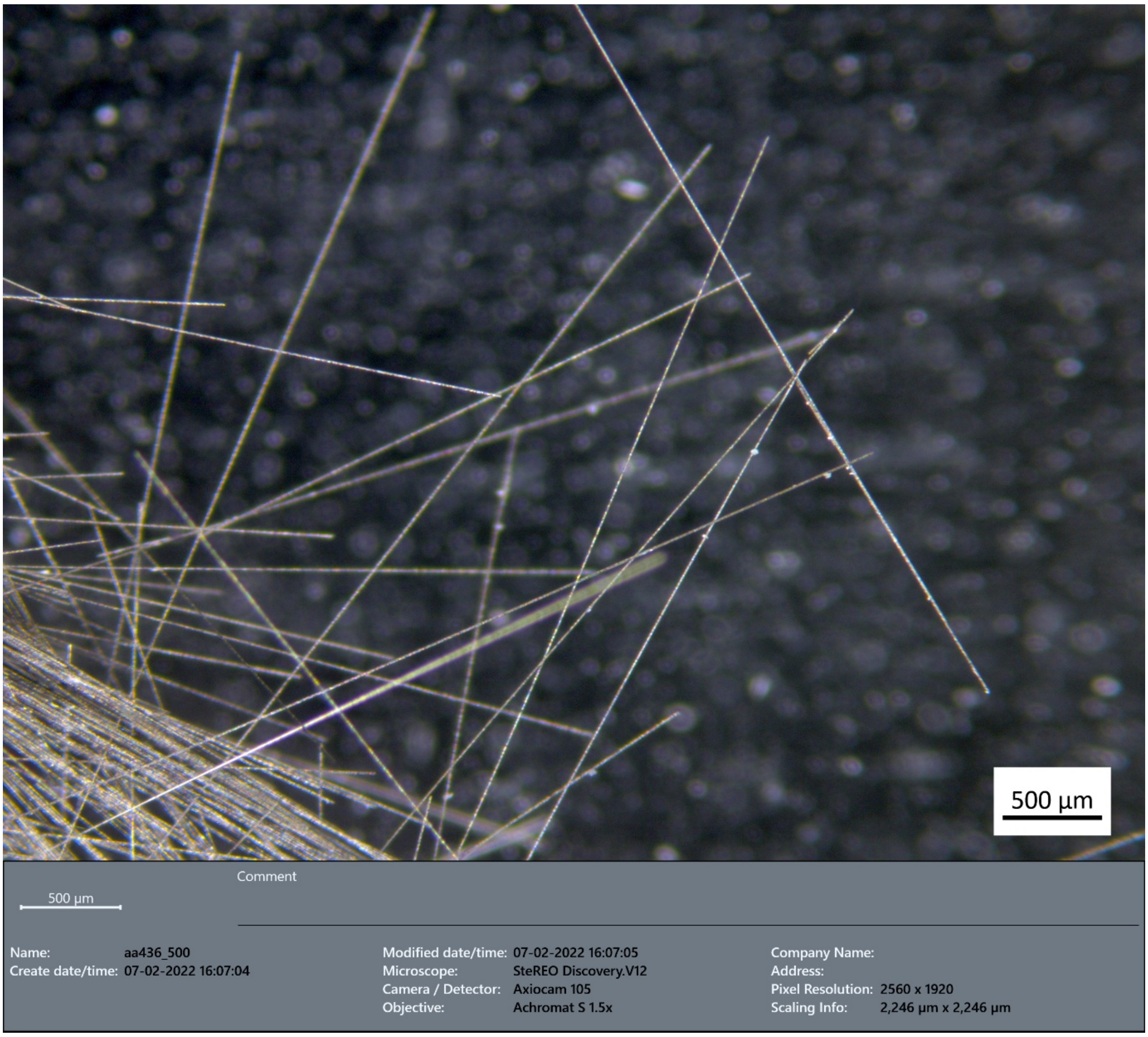

Fig. S13. Microscopic images of glass fibers recovered from fiberglass sample provided by Olin.

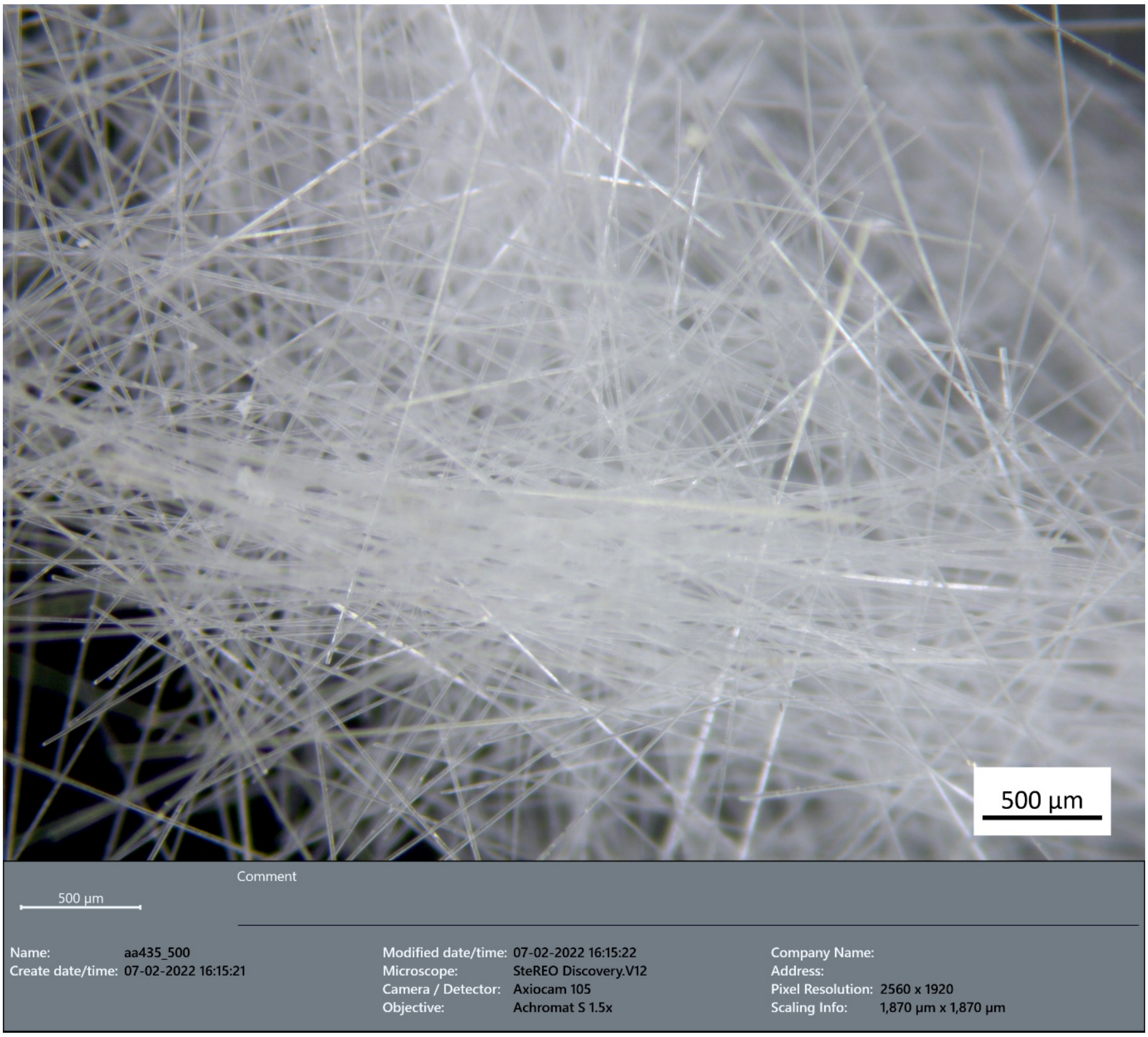

Fig. S14. Microscopic images of glass fibers recovered from the outer shell of a decommissioned wind turbine blade provided by Vestas.

6.3.2 IR spectra

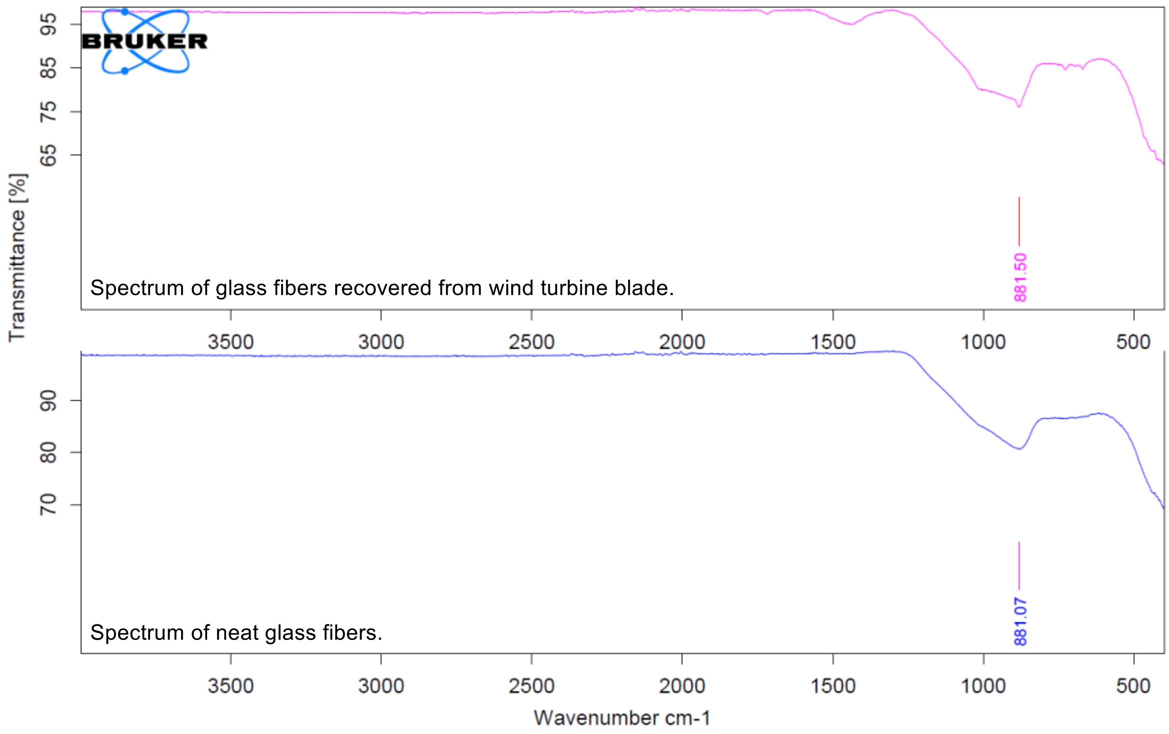

6.3.3 X-ray photoelectron spectroscopy

The chemical composition of the fibers were obtained by analysis of overview XPS spectra, Fig. S15. The atomic concentrations of Si, Ca, Al, assigned to the glass fibers are lower in the blank sample compared to the recovered fibers (Table SI-9). No residual epoxy is detected. The higher carbon content of the blank sample is assigned to removal of more carbon, both adventitious and from the priming layer, from the recovered fibers exposing the underlying glass fibers.
A single main peak centered at 284.4 eV corresponding to C–C and C–H bonds is observed. Because of the large full width at half maximum (FWHM), 2.6–2.8 eV, the presence of C–N/C–O single bonds or small charging effects cannot be excluded. Further, the π-π* type shake-up peaks typically detected for carbon in aromatic compounds (~291–292 eV) are absent, confirming the complete removal of the epoxy resin. The XPS results indicate that the process not only removes all of the epoxy polymer, but also the priming layer.

Table S9. Chemical composition of blank and recovered glass fibers derived from XPS.

Sample	O	C	N	Mg	Si	Ca	Al	Na	K
Glass Fibers (Blank)	21.0	73.1	1.1	0.1	3.3	0.1	1.0	0.0	0.0
Recovered Glass Fibers	44.2	31.9	1.7	0.2	15.5	2.3	3.9	0.4	Traces

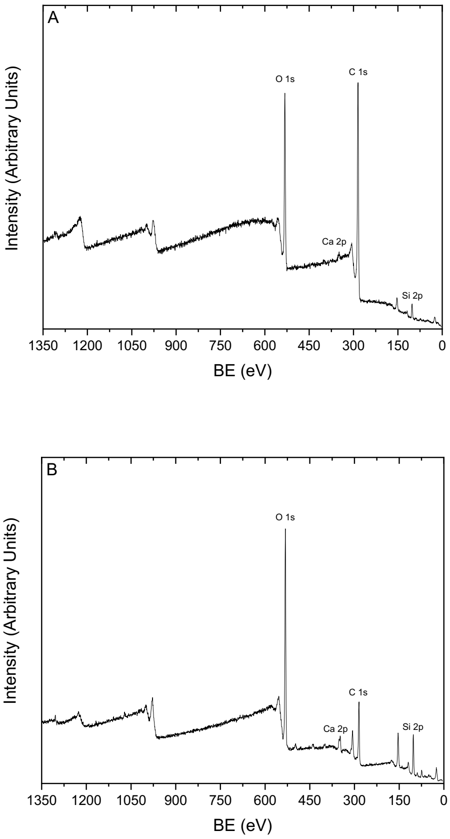

Fig. S15. XPS survey spectra of blank (A) and recovered (B) glass fibers.

6.3.4 SEM images

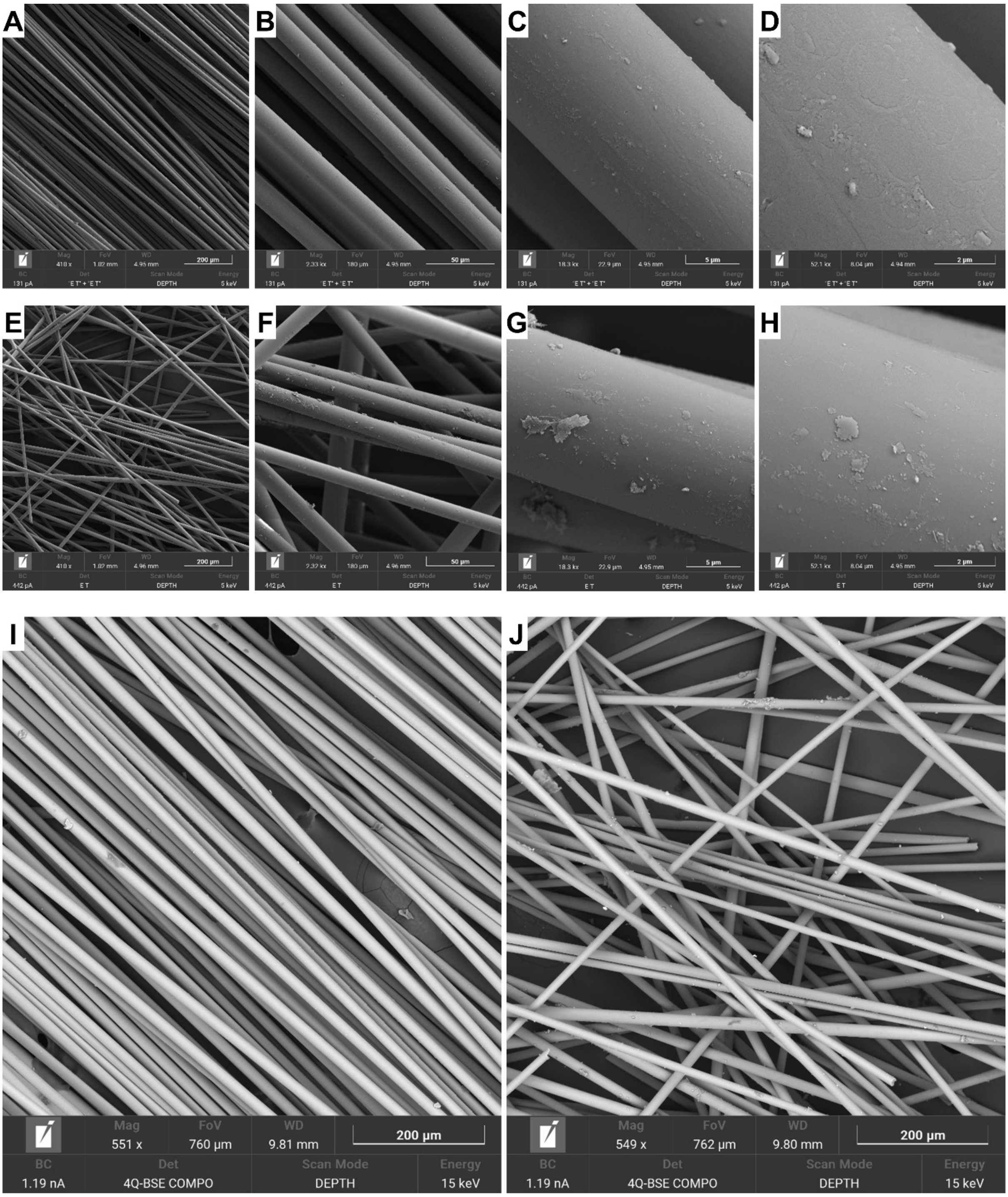

Fig S16. SEM images of neat and recovered glass fibers. SE contrast at increasing resolution shows the surface topography, revealing that the priming which coats the neat glass fibers (A-D) has been removed during the treatment, leaving the processed glass fibers bare, but intact (E-H). BSE contrast shows that the surface composition is uniform across both the neat (I) and processed glass fibers (J). There are thus no signs of left-over epoxy on processed fibers.

6.3.5 Tensile Strength Testing

Method

In order to prepare fibers for tensile strength testing, 4x8 cm pieces of white paper with a centered trapezoid window measuring 1 cm diagonally were prepared using a sterilized scalpel. The top and bottom of the paper strip was equipped with a small strip of double-sided tape (Tesa Film). Using a tweezer, a single glass fiber was picked with help of a microscope and gently lowered on to the tape in both ends in such way that the fiber string centered the two orthogonal corners of the trapezoid shape. UHU Plus Endfest 300 was used to fix the fibers in their place on both ends of the paper shape. Another piece of paper with a trapezoid window was added on top of the other as cover, so that the fiber was exposed in the center. These paper-supported fiber samples were left to cure at room temperature for 48 hours. The prepared samples were inserted into a tensile tester (Bose ElectroForce 5500, Bose Corp. Eden Prairie, Mn, USA) so that the glass fibers were vertically oriented. Once secured, the sides of the paper shape supporting the glass fiber were carefully cut with a fresh scalpel to have the singular fiber as the sole connection between the two tensile bars. Tensile tests were performed using a 0.5 mm/min rate.

8 samples of neat glass fibers and 8 samples of fibers recovered from the wind turbine blade (upscaled experiment) were prepared and measured.

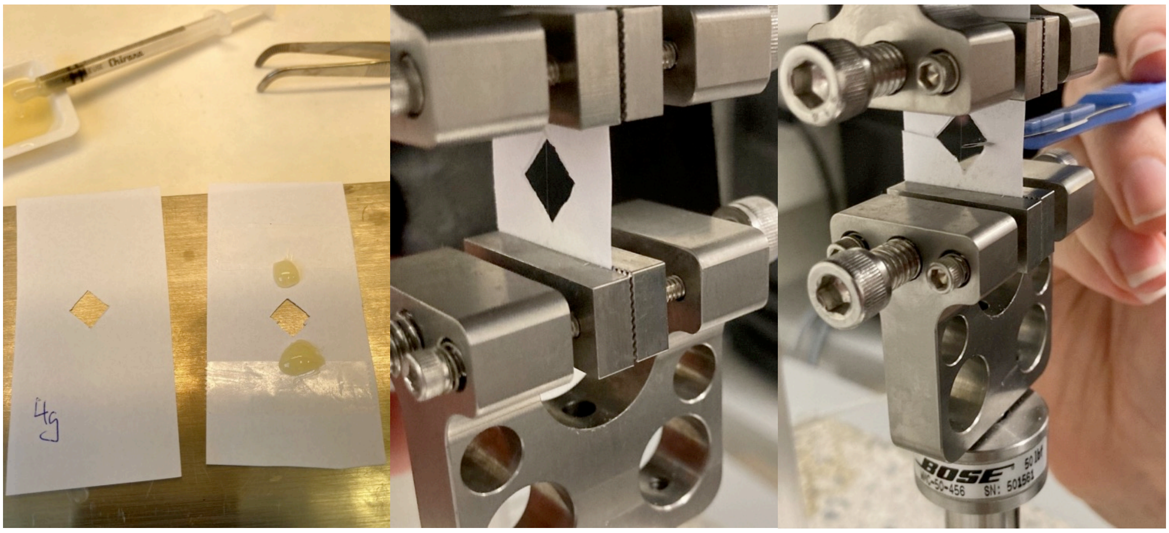

Fig S17. Preparation of paper-supported single fiber samples and insertion of single fibers in the tensile tester.

The tensile testing was applied across an approximated length of 1 cm of fiber, with the radius being approximated at 9 µm, resulting in a surface area of $2.5434 * 10^{-10}$ m^2.

The strain was calculated as:

$$strain = \frac{Disp - Disp(ini)}{10\ mm + Disp(ini)}$$

With Disp being the distance and Disp(ini) being the initial distance from which the fiber is stretched (first point where a force > 0 is observed).

The stress was calculated as:

$$stress = \frac{Force}{2.5434\ *\ 10^{-10}\ m^2}$$

The modulus was calculated as:

$$modulus = \frac{stress}{\text{strain}}$$

Modulus was calculated for each separate data point between 10% and 90% of the highest recorded stress (aka. The tensile strength @yield).

Results

Table S10. Results from tensile strength testing given as average of 8 samples each.

Sample	Force @ yield [N]	Tensile Strength @yield [GPa]	Modulus [GPa]
**Neat Fiber**	0.72 (0.12)	2.84 (0.46)	98.2 (21.1)
**Recovered Fiber**	0.63 (0.11)	2.45 (0.44)	93.8 (16.2)

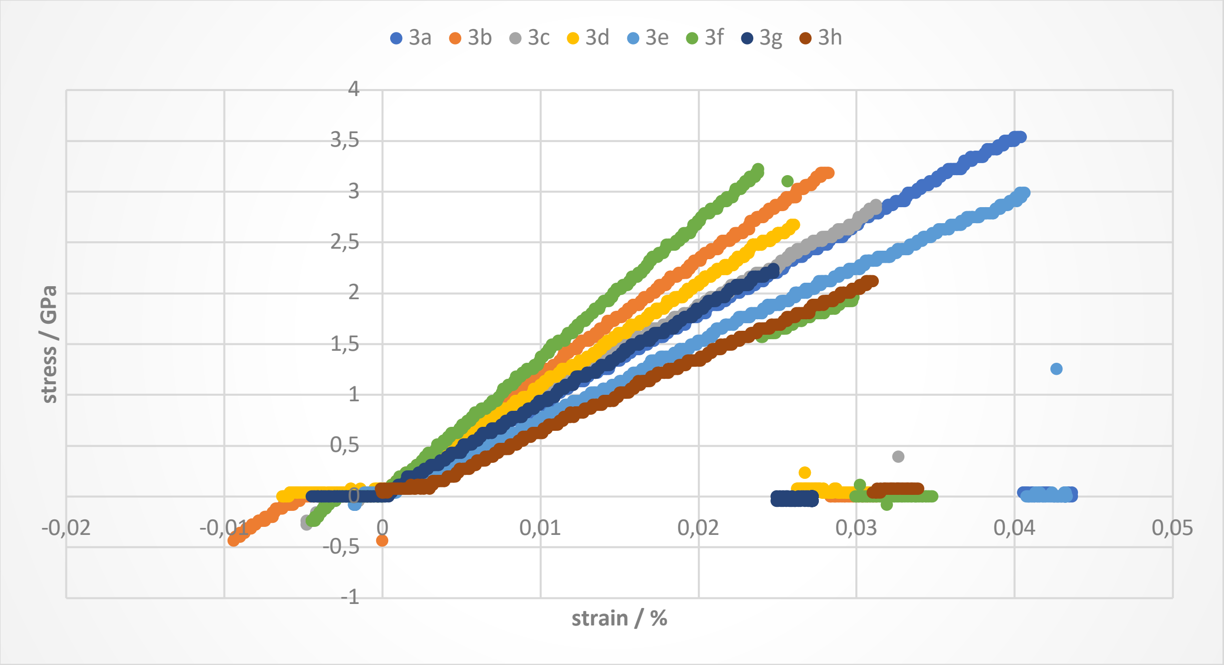

Fig. S18. Stress strain curves of virgin fibers (individual samples named 3a-3h).

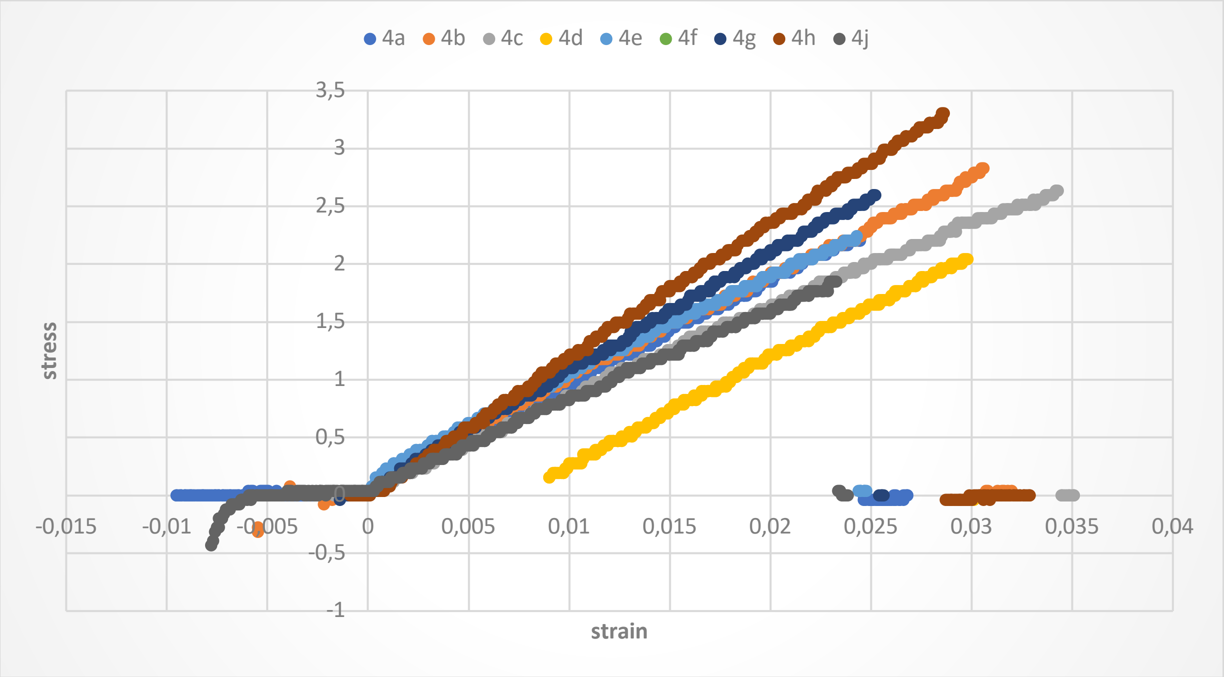

Fig. S18. Stress strain curves of recovered fibers (individual samples named 4a-4h).

6.4 X-Ray Crystallographic Data

During the attempted deconstruction of **model 9**,the ruthenium species showing a hydride signal at -6.78 ppm formed selectively from the precatalyst. Single crystals suitable for X-Ray crystallography were obtained from the reaction mixture yellow crystals precipitated from the reaction mixture after cooling to room temperature after 16 h. In an argon charged glovebox, the reaction mixture was decanted, and the precipitate dissolved in THF and then overlayered with pentane (vapour diffusion) at room temperature. Thereby, yellow crystals suitable for X-Ray Crystallography were obtained.

The intensities were empirically corrected for absorption using SCALE3 ABSPACK implemented in CrysAlisPRO[1]. (1) The unit cell parameters were determined, and the Bragg intensities were integrated using CrysAlisPRO. The structure was solved and refined with SHELXT in Olex2[2-4].

CCDC 2219777

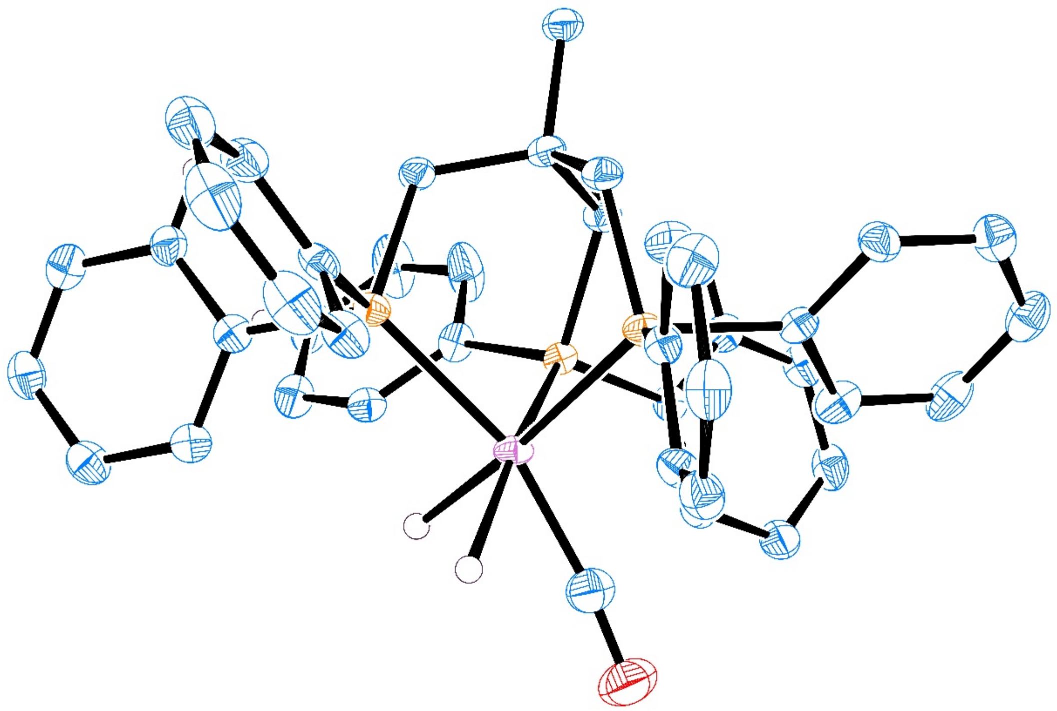

Fig. S19. Ortep representation of triphos-Ru-H_2-CO with ellipsoids at 50% probability. Blue = Carbon; Pink = Ruthenium; Orange = Phosphine; Red = Oxygen; White = Hydrogen. Non-metal hydrides have been omitted for clarity.

Table S11. Molecular Structure of triphos-Ru-H_2-CO in the Crystal.

Item	value
**Molecular formula**	$C_{42}H_{41}OP_3Ru$
**Formula weight**	755.73
**Crystal system**	orthorhombic
**Space Group**	$Pna2_1$
**a (Å)**	20.7024(6)
**b (Å)**	10.2083(2)
**c (Å)**	16.7222(5)
**α (°)**	90
**β (°)**	90
**γ (°)**	90
**Volume (Å^3)**	3534.01(16)
**Z**	4
**T (K)**	100
**ρ (g cm^{-1})**	1.420
**λ (Å)**	0.71073
**μ (mm^{-1})**	0.612
**# measured refl**	30845
**# unique refl**	11573
**R_{int}**	0.0451
**# parameters**	433
**R(F^2), all refl**	0.0535
**Rw(F^2), all refl**	0.0907
**Goodness of fit**	1.034

6.5 Density Functional Theory (DFT) Study

Computational details: All the reported DFT results were obtained with the Gaussian 16 package, at 1 atm and 298.15 K[25]. All molecules had their structure optimized at the (U)M06-2X/6-311++G(d,p) level of theory, in the gas phase[26–28]. To confirm that the structures are an energy minimum, vibrational analysis was done at the same level of theory, which also provides the energy corrections for enthalpy. Previous studies on lignin bond dissociation energy (BDE) have demonstrated that the (U)M062X/6-311++G(d,p) level of theory is sufficient for the obtention of reliable results[29,30]. All structures were treated as full models with no symmetry constraints. Conformational analysis was first manually searched, and further analysis was done with minimization of the structures using molecular dynamics (MD) with 100 steepest descent steps with 0.02 Å step size using UCSF Chimera version 1.14[31]. The enthalpy value for each structure was obtained with the sum of the total electronic energy of the optimized structure with the thermal correction to the enthalpy as described by Ph.D. Joseph Ochterski[32]. The BDE's were calculated by using the thermochemical scheme supplied by Gaussian, and are obtained as the difference of the sum of the enthalpies of the fragments and the enthalpy of the molecule[32,33].

Table S12. Energies for the molecules - BDE calculation (calculated at the UM062X/6-311++G(d,p) level of theory).

	$E_{298.15}$ kcal mol^{-1}	$G_{298.15}$ kcal mol^{-1}	$H_{298.15}$ kcal mol^{-1}
**Epoxy model 1**	-1087860.28214725	-1087480.23802725	-1087408.18784975
**Epoxy models fragment 1**	-483309.5260855	-483154.596963	-483115.725848
**Epoxy model 1 fragment 2**	-604478.6847375	-604273.5562425	-604225.7783925
**Epoxy models fragment 1 C-C**	-507952.28296225	-507781.30929225	-507740.17290225
**Epoxy model 1 fragment 2 C-C**	-579819.57345375	-579629.41899125	-579584.40590625
**Ketone 1**	-1087098.275075	-1086734.3984925	-1086661.8607475
**Ketone 1 fragment 2**	-603727.96701775	-603536.57073275	-603489.79060775
**Ketone 1 fragment 2 C-C**	-579062.88802075	-578887.36121075	-578842.79678825
**Alcohol III**	-604900.4282405	-604684.885128	-604638.1646155
**Alcohol III fragment 2**	-121516.2634145	-121474.7718595	-121453.043417
**Alcohol III fragment 2 C-C**	-96857.21751625	-96831.74101625	-96812.07642125
**Ketone III**	-604141.57172025	-603942.44902775	-603894.98743775
**Ketone III fragment 2**	-120767.86932725	-120741.18237975	-120719.65222725
**Ketone III fragment 2 C-C**	-96105.1253205	-96093.923818	-96074.723573

DFT calculations of the depicted models and intermediates were performed to obtain the bond dissociation energy (BDE) values for both C–C and C–O bonds (Fig. S20). The results unveil that the BDEs for the C–O linkage in the ketone intermediates (Ketone **I** and Ketone **III**) are smaller than the ones of the corresponding alcohols (**Model 1** and Alcohol **III**), which corroborates with our proposal that these ketone intermediates are key species that exist prior to the C–O cleavage by the catalyst. The calculations also reveal that the BDE is smaller for the C–O linkage compared to the C–C linkages, even when comparing the lowest found C–C linkage BDE (Ketone **I**, 78.9 kcal mol^{-1}) to the highest found C–O linkage BDE (Alcohol **III**, 69.4 kcal mol^{-1}), a sharp difference is observed, inferring that the C–C linkage cleavage is too energetically demanding.

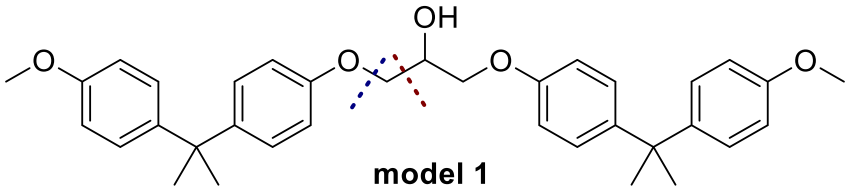

model 1

BDE (kcal mol^{-1})
C-O: 66.7
C-C: 83.6

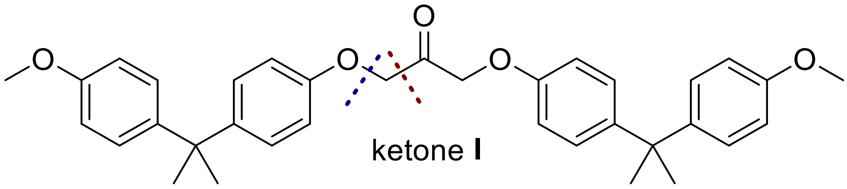

ketone **I**

BDE (kcal mol^{-1})
C-O: 56.3
C-C: 78.9

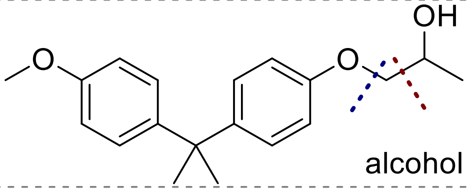

alcohol **III**

BDE (kcal mol^{-1})
C-O: 69.4
C-C: 85.9

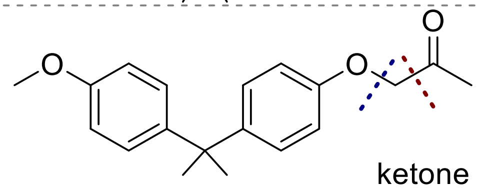

ketone **III**

BDE (kcal mol^{-1})
C-O: 59.6
C-C: 80.1

Fig. S20. BDE calculation for epoxy models and intermediates, all presented results were obtained at the (U)M06-2X/6-311++G(d,p) level of theory

7. References

1. Fulmer, G. R., Miller, A. J. M., Sherden, N. H., Gottlieb, H. E., Nudelman, A., Stoltz, B. M., Bercaw, J. E., Goldberg, K. I. NMR Chemical Shifts of Trace Impurities: Common Laboratory Solvents, Organics, and Gases in Deuterated Solvents Relevant to the Organometallic Chemist. *Organometallics* **29**, 2176-2179 (2010).
2. Wittig, N. K., Østergaard, M., Palle, J., Christensen, T. E. K., Langdahl, B. L., Rejnmark, L., Hauge, E.-M., Brüel, A., Thomsen, J. S., Birkedal, H. Opportunities for biomineralization research using multiscale computed X-ray tomography as exemplified by bone imaging. *J. Struct. Biol.* **214**, 107822 (2022).
3. Nichols, J. M., Bishop, L. M., Bergman, R. G., Ellman, J. A. Catalytic C−O Bond Cleavage of 2-Aryloxy-1-arylethanols and Its Application to the Depolymerization of Lignin-Related Polymers. *J. Am. Chem. Soc.* **132**, 12554-12555 (2010).
4. vom Stein, T., Weigand, T., Merkens, C., Klankermayer, J., Leitner, W. Trimethylenemethane-Ruthenium(II)-Triphos Complexes as Highly Active Catalysts for Catalytic C−O Bond Cleavage Reactions of Lignin Model Compounds. *ChemCatChem* **5**, 439-441 (2013).
5. Johnson, T. C., Morris, D. J., Wills, M. Hydrogen generation from formic acid and alcohols using homogeneous catalysts. *Chem. Soc. Rev*. **39**, 81–88 (2010).
6. Westhues, N., Klankermayer, J. *ChemCatChem* **11**, 3371-3375 (2019).
7. Khusnutdinova, J. R., Milstein, D. Metal–Ligand Cooperation. *Angew. Chem. Int. Ed.* **54**, 12236–12273 (2015).
8. Scherl, P., Kruckenberg, A., Mader, S., Wadepohl, H., Gade, L. H. Ruthenium η^4-Trimethylenemethane Complexes Containing Tripodal Phosphanomethylamine Ligands. *Organometallics* **31**, 7024–7027 (2012).
9. vom Stein, T., Meuresch, M., Limper, D., Schmitz, M., Hölscher, M., Coetzee, J., Cole-Hamilton, D. J., Klankermayer, J. Leitner, W. Highly Versatile Catalytic Hydrogenation of Carboxylic and Carbonic Acid Derivatives using a Ru-Triphos Complex: Molecular Control over Selectivity and Substrate Scope. *J. Am. Chem. Soc.* **136**, 13217-13225 (2014).
10. Nakagawa, N., Derrah, E. J., Schelwies, M., Rominger, F., Trapp, O., Schaub, T. Triphos derivatives and diphosphines as ligands in the ruthenium-catalysed alcohol amination with NH_3. *Dalton Trans.* **45**, 6856-6865 (2016).
11. Geilen, F. M. A., Engendahl, B., Hölscher, M., Klankermayer, J., Leitner, W. Selective Homogeneous Hydrogenation of Biogenic Carboxylic Acids with $[Ru(TriPhos)H]^+$: A Mechanistic Study. *J. Am. Chem. Soc.* **133**, 14349-14358 (2011).
12. Bakhmutov, V. I., Bakhmutova, E. V., Belkova, N. V., Bianchini, C., Epstein, L. M., Masi, D., Peruzzini, M., Shubina, E. S., Vorontsov, E. V., Zanobini, F. In-depth NMR and IR study of the proton transfer equilibrium between $[\{(MeC(CH_2PPh_2)_3\}Ru(CO)H_2]$ and hexafluoroisopropanol. *Can. J. Chem.* **79**, 479-489 (2001).
13. Westhues, N., Belleflamme, M., Klankermayer, J. Base-Free Hydrogenation of Carbon Dioxide to Methyl Formate with a Molecular Ruthenium-Phosphine Catalyst. *ChemCatChem* **11**, 5269-5274 (2019).
14. Leopold, M., Siebert, M., Siegle, A. F., Trapp, O. Reaction Network Analysis of the Ruthenium-Catalyzed Reduction of Carbon Dioxide to Dimethoxymethane. *ChemCatChem* **13**, 2807-2814 (2021).

15. Siebert, M., Seibicke, M., Siegle, A. F., Kräh, S., Trapp, O. Selective Ruthenium-Catalyzed Transformation of Carbon Dioxide: An Alternative Approach toward Formaldehyde. *J. Am. Chem. Soc.* **141**, 334-341 (2019).
16. Hanada, S., Yuasa, A., Kuroiwa, H., Motoyama, Y., Nagashima, H. Hydrosilanes Are Not Always Reducing Agents for Carbonyl Compounds, II: Ruthenium-Catalyzed Deprotection of tert-Butyl Groups in Carbamates, Carbonates, Esters, and Ethers. *Eur. J. Org. Chem.* **2010**, 1021-1025 (2010).
17. Harvey, B. G., Guenthner, A. J., Koontz, T. A., Storch, P. J., Reams, J. T., Groshens, T. J. Sustainable hydrophobic thermosetting resins and polycarbonates from turpentine. *Green Chem.* **18**, 2416-2423 (2016).
18. Huang, G. Yin, B. Palladium-Catalyzed Cross-Coupling of Furfuryl Alcohols with Arylboronic Acids via Aromatization-Driven Carbon−Carbon Bond Cleavage to Synthesize 5-Arylfurfuryl Alcohols and 2,5-Diaryl Furans. *Adv. Synth. Catal.* **361**, 5576-5586 (2019).
19. Pan, W., Li, C., Zhu, H., Li, F., Li, T., Zhao, W. A mild and practical method for deprotection of aryl methyl/benzyl/allyl ethers with $HPPh_2$ and *t*BuOK. *Org. Biomol. Chem.* **19**, 7633-7640 (2021).
20. Chiang, H.-C., Lin, L.-J. Quantitative analysis of a mixture of cresols by carbon-13 NMR spectroscopy. *Org. Magn. Reson.* **12**, 260-262 (1979).
21 CrysAlisPRO, Oxford Diffraction /Agilent Technologies UK Ltd, Yarnton, England
22. Dolomanov, O.V.; Bourhis, L.J.; Gildea, R.J.; Howard, J.A.K.; Puschmann, H. OLEX2: a complete structure solution, refinement and analysis program *J. Appl. Cryst.* **42**, 339–341 (2009).
23. Sheldrick, G.M. A short history of SHELX *Acta Cryst*. **A64**, 112–122 (2008).
24. Sheldrick, G.M. Crystal structure refinement with SHELXL *Acta Cryst.* **C71**, 3–8 (2015).
25. Frisch, M. J.; Trucks, G. W.; Schlegel, H. B.; Scuseria, G. E.; Robb, M. A.; Cheeseman, J. R.; Scalmani, G.; Barone, V.; Petersson, G. A.; Nakatsuji, H.; Li, X.; Caricato, M.; Marenich, A. V.; Bloino, J.; Janesko, B. G.; Gomperts, R.; Mennucci, B.; Hratch, D. J. Gaussian 16, Revision B.01. Gaussian, Inc.,: Wallingford CT 2016.
26. Krishnan, R.; Binkley, J. S.; Seeger, R.; Pople, J. A. Self-consistent Molecular Orbital Methods. XX. A Basis Set for Correlated Wave Functions. *J. Chem. Phys.* **72**, 650–654 (1980).
27. Clark, T.; Chandrasekhar, J.; Spitznagel, G. W.; Schleyer, P. V. R. Efficient Diffuse Function-Augmented Basis Sets for Anion Calculations. III. The 3-21+G Basis Set for First-Row Elements, Li-F. *J. Comput. Chem.* **4**, 294–301 (1983). https://doi.org/10.1002/jcc.540040303.
28. Zhao, Y.; Truhlar, D. G. The M06 Suite of Density Functionals for Main Group Thermochemistry, Thermochemical Kinetics, Noncovalent Interactions, Excited States, and Transition Elements: Two New Functionals and Systematic Testing of Four M06-Class Functionals and 12 Other Function. *Theor. Chem. Acc.* **120**, 215–241 (2008).
29. Parthasarathi, R.; Romero, R. A.; Redondo, A.; Gnanakaran, S. Theoretical Study of the Remarkably Diverse Linkages in Lignin. *J. Phys. Chem. Lett.* **2**, 2660–2666 (2011).
30. Beste, A.; Buchanan, A. C. Computational Study of Bond Dissociation Enthalpies for Lignin Model Compounds. Substituent Effects in Phenethyl Phenyl Ethers. J. *Org. Chem.* **74**, 2837–2841 (2009).

31. Pettersen, E. F.; Goddard, T. D.; Huang, C. C.; Couch, G. S.; Greenblatt, D. M.; Meng, E. C.; Ferrin, T. E. UCSF Chimera A Visualization System for Exploratory Research and Analysis. *J. Comput. Chem.* **25**, 1605–1612 (2004).
32. Ochterski, J. W. Thermochemistry in Gaussian https://gaussian.com/thermo/ (accessed Nov 10, 2022).
33. Yao, X.-Q.; Hou, X.-J.; Jiao, H.; Xiang, H.-W.; Li, Y.-W. Accurate Calculations of Bond Dissociation Enthalpies with Density Functional Methods. *J. Phys. Chem. A*, **107**, 9991–9996 (2003).
